# CAF-derived TGF-β1 activates an LBH-mediated β-catenin stabilization program to drive gastric cancer progression

**DOI:** 10.1038/s41419-026-09150-w

**Published:** 2026-08-06

**Authors:** Zhixiong Su, Guifeng Zhang, Jiangming Zhong, Lijie You, Yufang He, Jingbo Chen, Zhenhua Liu

**Affiliations:** Department of Oncology, Shengli Clinical Medical College of Fujian Medical University, Fujian Provincial Hospital, Fuzhou University Affiliated Provincial Hospital, Fuzhou, Fujian PR China

**Keywords:** Cancer microenvironment, Gastric cancer, Ubiquitylation, Tumour biomarkers

## Abstract

Aberrant Wnt/β-catenin signaling is frequently observed in gastric cancer (GC); however, the mechanisms sustaining this pathway in the absence of canonical genetic mutations remain incompletely understood. Here we show that the transcriptional cofactor LBH drives mutation-independent Wnt activation and malignant progression in GC via a tumour microenvironment-regulated post-translational stabilization mechanism. By integrating single-cell transcriptomics with multicentre clinical cohorts, we identify LBH as a principal regulator of epithelial–mesenchymal transition and peritoneal metastasis, and its elevated expression independently predicts poor patient survival. Mechanistically, fi broblast activation protein (FAP)-positive cancer-associated fi broblasts (CAFs) secrete TGF-β1, which selectively induces LBH expression in adjacent GC cells via the SMAD2/3 signaling cascade. Crucially, LBH physically interacts with β-catenin, providing steric hindrance that prevents destruction complex-mediated phosphorylation and subsequent ubiquitin-proteasomal degradation. This FAP⁺ CAF–TGF-β1–LBH–β-catenin paracrine axis continuously sustains global Wnt transcriptional output without requiring intrinsic genetic mutations. Our findings elucidate a critical tumour–stroma crosstalk mechanism and establish the targeted disruption of the LBH–β-catenin interaction interface as a clinically relevant therapeutic strategy for advanced gastric cancer.

**Figure:**
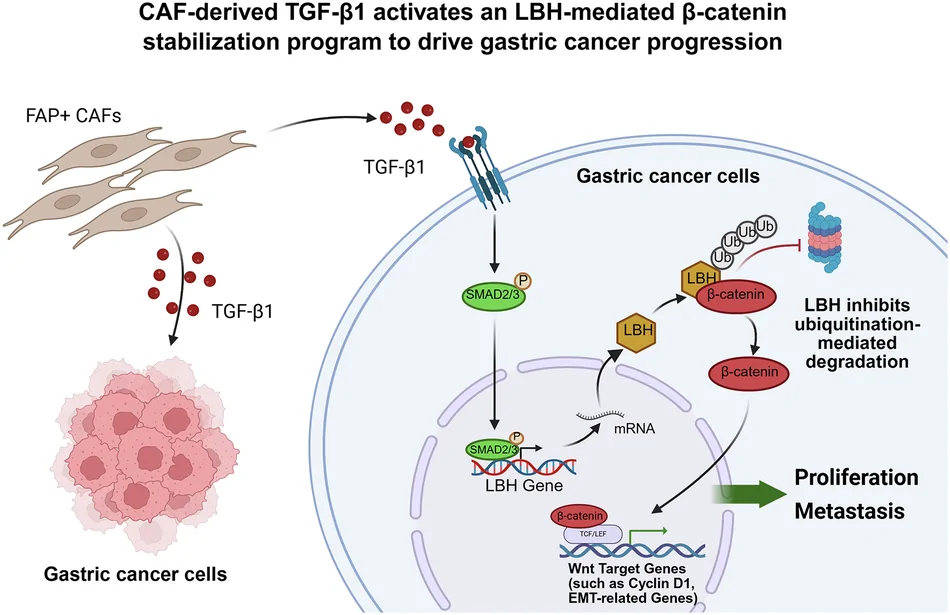
This study reveals a novel paracrine mechanism in gastric cancer whereby FAP⁺ CAF-derived TGF-β1 upregulates LBH via SMAD2/3 signaling specifically in tumor cells. By physically interacting with β-catenin in situ and stereospecifically shielding it from destruction complex-mediated phosphorylation, LBH substantially attenuates its ubiquitination and proteasomal degradation, thereby robustly sustaining the activation of downstream Wnt target genes. This stromal-tumor crosstalk axis significantly promotes cancer proliferation and metastasis, highlighting the targeted disruption of the LBH–β-catenin interface as a highly promising therapeutic strategy.

This study reveals a novel paracrine mechanism in gastric cancer whereby FAP⁺ CAF-derived TGF-β1 upregulates LBH via SMAD2/3 signaling specifically in tumor cells. By physically interacting with β-catenin in situ and stereospecifically shielding it from destruction complex-mediated phosphorylation, LBH substantially attenuates its ubiquitination and proteasomal degradation, thereby robustly sustaining the activation of downstream Wnt target genes. This stromal-tumor crosstalk axis significantly promotes cancer proliferation and metastasis, highlighting the targeted disruption of the LBH–β-catenin interface as a highly promising therapeutic strategy.

## Introduction

Gastric cancer (GC) remains one of the leading causes of cancer-related mortality worldwide and represents a major global health burden, particularly in East Asia [1, 2]. Despite advances in early diagnosis and multimodal treatment strategies, the prognosis of patients with advanced GC remains poor due to frequent metastasis and recurrence [3]. Among metastatic patterns, peritoneal dissemination is the most common and lethal form, highlighting the urgent need to elucidate the molecular mechanisms that drive GC progression and metastatic spread [4].

Accumulating evidence indicates that aberrant activation of the Wnt signaling pathway plays a pivotal role in tumor initiation, progression, and metastasis in multiple malignancies, including GC [5–7]. Canonical Wnt signaling regulates essential biological processes such as stem cell maintenance, cell proliferation, and epithelial–mesenchymal transition (EMT) [8]. In certain cancers, constitutive activation of Wnt signaling is driven by genetic alterations in key pathway components such as APC, CTNNB1, and AXIN [9]. However, genomic analyses of GC have revealed that such mutations occur only in a small subset of tumors [10]. This discrepancy indicates that non-mutational mechanisms—particularly those driven by the tumor microenvironment (TME)—must be responsible for sustaining persistent Wnt/β-catenin activity during gastric tumorigenesis. Yet, the specific regulators bridging TME cues and mutation-independent Wnt activation remain poorly defined.

LBH (Limb Bud and Heart Development) is a transcriptional cofactor originally identified as a developmental regulator involved in embryonic organogenesis and tissue patterning [11, 12]. Previous studies have suggested that LBH functions downstream of Wnt signaling during embryonic development [13]. Emerging evidence further indicates that LBH contributes to tumor progression in several malignancies by regulating cell proliferation, migration, and differentiation programs [14, 15]. Notably, whether LBH can reciprocally modulate the stability of the Wnt/β-catenin pathway in GC, and how its own expression is transcriptionally and environmentally regulated within the TME, remains largely unexplored.

Recent advances in single‑cell RNA sequencing (scRNA-seq) have enabled high-resolution characterization of tumor heterogeneity and lineage dynamics within the tumor microenvironment [16]. Single-cell studies have revealed complex epithelial differentiation trajectories and transcriptional programs that contribute to gastric tumor evolution [17–19]. However, the molecular regulators that connect epithelial lineage plasticity with oncogenic signaling pathways remain incompletely understood. Crucially, previous studies have predominantly positioned LBH as a downstream transcriptional target of embryonic development, leaving a significant gap in our understanding of how LBH expression is environmentally regulated within the TME and how it reciprocally modulates intracellular oncogenic cascades.

In the present study, we address this critical gap by integrating scRNA-seq analysis with comprehensive in vivo and in vitro models to uncover a novel, non-mutational mechanism of Wnt pathway activation in GC. Our research provides two key innovations: First, we identify FAP^+^ cancer-associated fibroblasts (CAFs) as the primary upstream source of paracrine TGF-β1, which drives the SMAD2/3-dependent transcriptional activation of LBH specifically in adjacent epithelial cells, establishing LBH as a tumor-intrinsic node rather than a stromal autocrine target. Second, we elucidate a previously unrecognized post-translational mechanism wherein LBH physically interacts with β-catenin in situ, shielding it from ubiquitin-mediated proteasomal degradation and thereby robustly rescuing downstream Wnt transcriptional output. This paracrine FAP^+^ CAFs/TGF-β1/LBH/β-catenin signaling axis not only sustains mutation-independent Wnt activation but also potently drives GC proliferation, epithelial-mesenchymal transition (EMT), and peritoneal dissemination. Ultimately, our findings establish LBH-mediated β-catenin stabilization as a highly promising therapeutic vulnerability in advanced GC.

## Results

### Single-cell transcriptomic analysis reveals the malignant evolutionary trajectory of gastric cancer epithelial cells

**Fig. 1:**
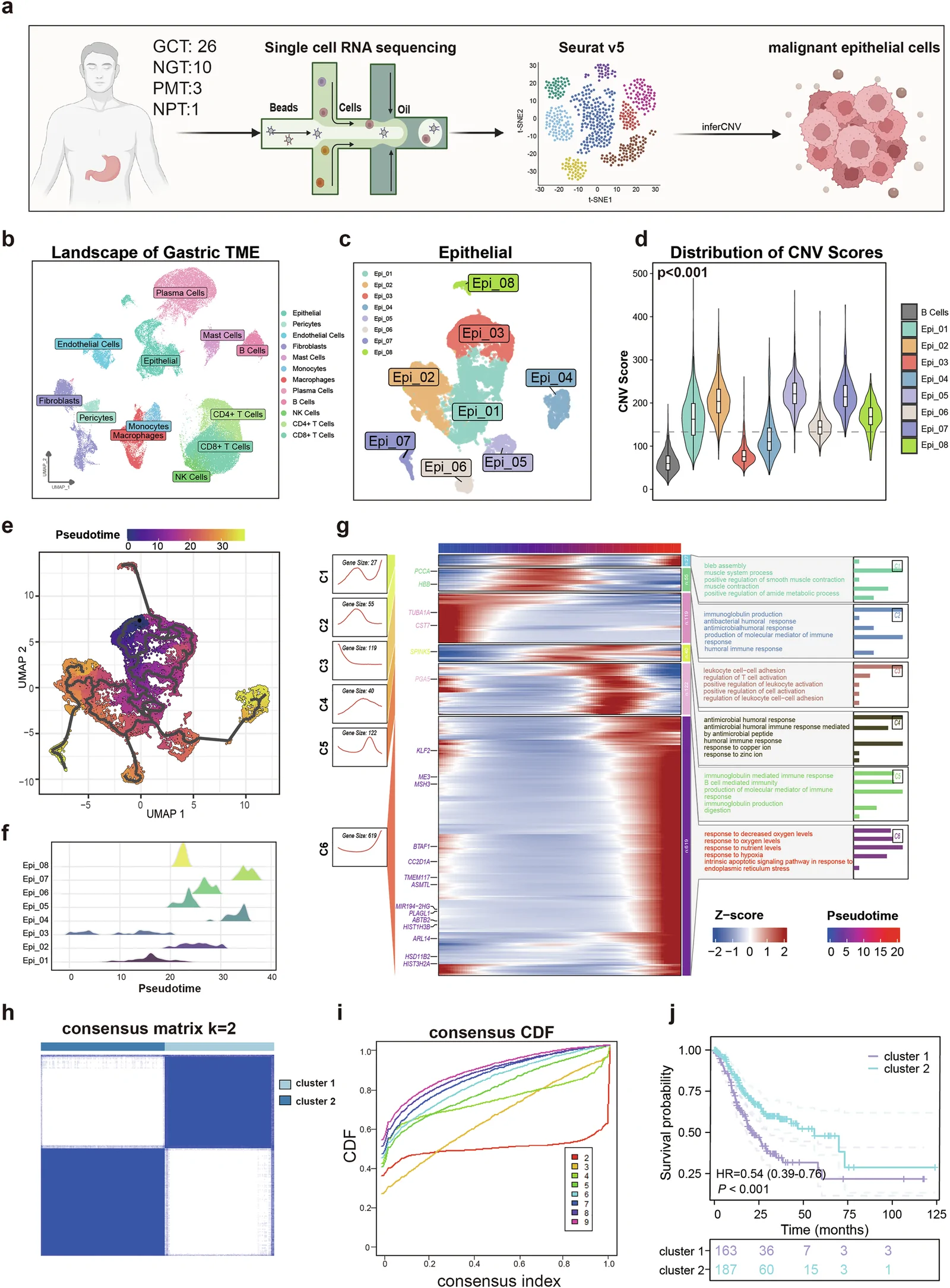
Single-cell transcriptomic profiling reveals the malignant evolutionary trajectory of gastric cancer epithelial cells. **a** Workflow of single-cell RNA sequencing (scRNA-seq) and downstream analysis. UMAP plots displaying the overall cellular landscape of the tumor microenvironment (**b**) and eight distinct epithelial sub-clusters (**c**). **d** Violin plots of chromosomal copy number variation (CNV) scores across the epithelial sub-clusters (*P* < 0.001). Pseudotime trajectory mapped onto the UMAP space (**e**) and corresponding ridge density plots (**f**) illustrating malignant evolutionary dynamics. **g** Heatmap of highly variable signature genes along the pseudotime trajectory, clustered into six functional modules (C1–C6) with associated GO enrichment terms. Non-negative matrix factorization (NMF) consensus matrix for k = 2 (**h**) and cumulative distribution function (CDF) curves (**i**). **j** Kaplan-Meier overall survival curves of patients stratified by the NMF consensus subtypes (cluster 1 vs. cluster 2). Statistical significance was determined by the Cox regression. scRNA-seq single-cell RNA sequencing, UMAP Uniform Manifold Approximation and Projection, CNV copy number variation, GO Gene Ontology, NMF non-negative matrix factorization, CDF cumulative distribution function.

To comprehensively characterize the cellular landscape of the gastric cancer (GC) tumor microenvironment (TME), we analyzed single-cell RNA sequencing (scRNA-seq) data derived from gastric cancer tissues (GCT, *n* = 26), adjacent normal tissues (NGT, *n* = 10), peritoneal metastatic tissues (PMT, *n* = 3), and normal peritoneal tissue (NPT, *n* = 1) (Figs. 1a and S1a). After stringent quality control and integration, unsupervised clustering identified multiple major cell populations within the TME, including epithelial cells, fibroblasts, endothelial cells, and diverse immune cell subsets (Figs. 1b and S1b).

As gastric cancer originates from the epithelial lineage, we next performed refined sub-clustering analysis and identified eight epithelial subpopulations (Epi_01–Epi_08) (Fig. 1c). To evaluate their malignant potential, inferCNV analysis was conducted using B cells as a reference population. Notably, epithelial clusters exhibited markedly heterogeneous chromosomal copy number variation (CNV) patterns, suggesting distinct stages of malignant transformation among tumor epithelial cells (*P* < 0.001; Figs. 1d and S1c).

To further reconstruct the dynamic process of epithelial tumor evolution, pseudotime trajectory analysis was performed. The trajectory revealed a continuous developmental progression originating from relatively low-CNV epithelial populations toward multiple highly malignant states (Figs. 1e, f and S1d). Analysis of gene expression dynamics along the pseudotime axis identified six transcriptional modules (C1–C6) with distinct expression patterns. Among them, module C6 genes displayed a progressive and pronounced increase during the late stages of the trajectory, suggesting their potential involvement in driving malignant progression (Fig. 1g).

To assess the clinical relevance of these transcriptional programs, we applied non-negative matrix factorization (NMF) specifically based on the expression profiles of C6 module genes to stratify GC patients into two molecular subtypes (cluster 1 and cluster 2) (Figs. 1h, i and S2a). Kaplan–Meier survival analysis revealed that patients in cluster 2 exhibited significantly better overall survival (OS) compared with cluster 1 (HR = 0.54, 95% CI: 0.39–0.76, *P* < 0.001; Fig. 1j). Consistent with the dynamic increase of C6 genes along the malignant trajectory, cluster 1, which was characterized by high C6 module activity, was enriched in EMT and Wnt signaling (*P* < 0.05; Fig. S2b-d). Collectively, these findings suggest that the C6 gene module represents a critical driver of malignant epithelial evolution in GC. We therefore sought to identify the specific core regulators underlying these oncogenic transcriptional programs.

### Aberrantly high expression of LBH is an independent prognostic risk factor in gastric cancer

**Fig. 2:**
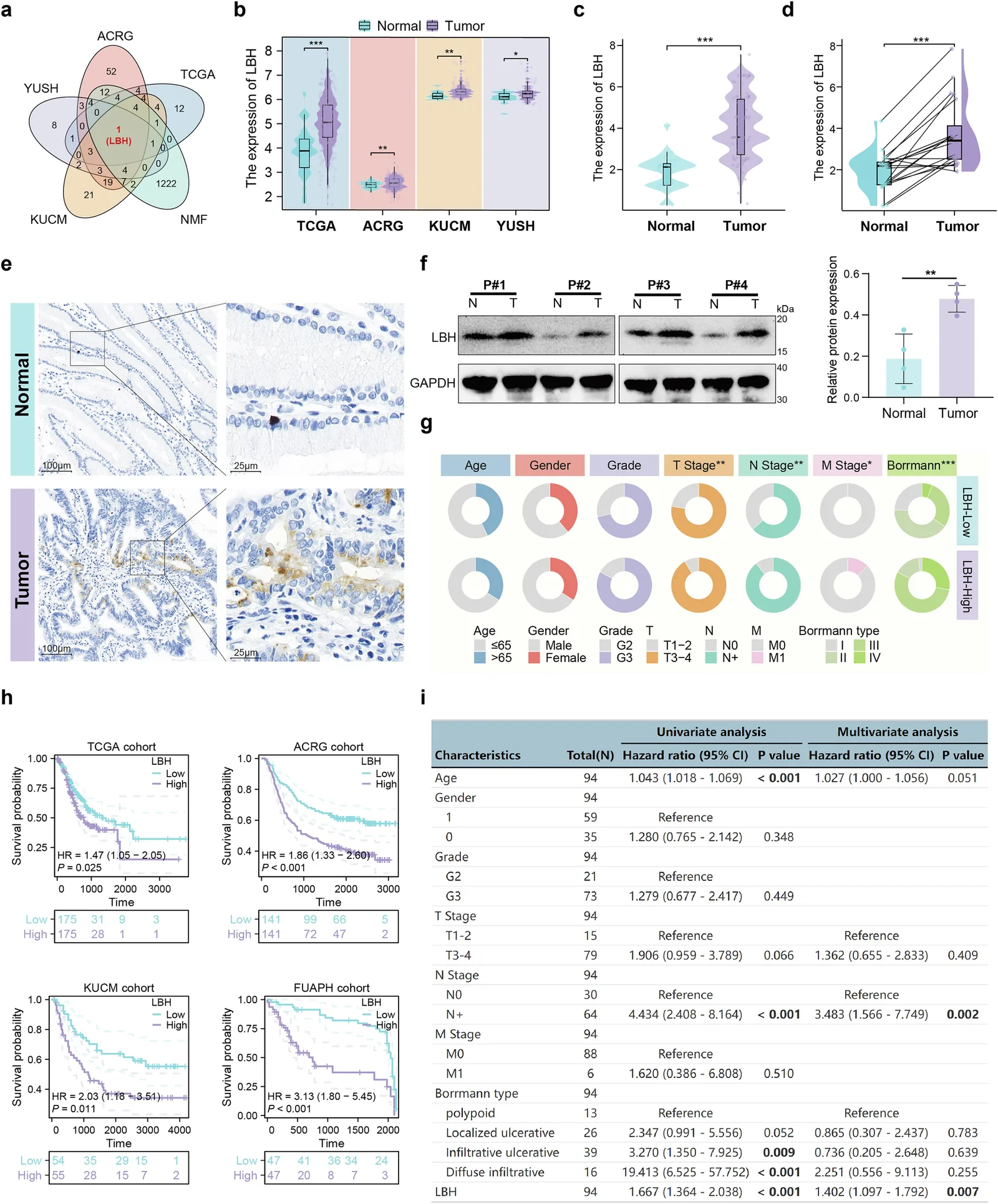
Aberrantly high expression of LBH correlates with advanced clinicopathological features and predicts poor prognosis in gastric cancer. **a** Venn diagram illustrating the intersection of non-negative matrix factorization (NMF)-derived differentially expressed genes with prognostic genes in C6 clusters across four independent public gastric cancer cohorts (TCGA, ACRG, YUSH, and KUCM). *LBH* was identified as the sole consistently enriched core target. **b** *LBH* mRNA expression in tumor versus normal tissues across the indicated public cohorts. Unpaired (**c**) and paired (**d**) qPCR analyses of *LBH* mRNA expression in the independent FUAPH cohort. **e** Representative IHC images of LBH in paired normal and gastric tumor tissues. Scale bars: 100 μm and 25 μm (magnified insets). **f** Western blot and corresponding quantification of LBH protein in representative paired normal (N) and tumor (T) tissues. GAPDH serves as the loading control. **g** Donut charts summarizing the correlation between LBH expression and clinicopathological characteristics. **h** Kaplan-Meier overall survival curves for patients stratified by LBH expression across four cohorts. **i** Univariate and multivariate Cox regression analyses identifying independent prognostic factors for overall survival in the FUAPH cohort. Data are presented as mean ± SD. Statistical significance was analyzed by Student’s *t*-test, Wilcoxon rank-sum test, or Cox regression. * *P* < 0.05, ** *P* < 0.01, *** *P* < 0.001. NMF non-negative matrix factorization, HR hazard ratio, CI confidence interval.

To pinpoint the most clinically relevant driver molecule from the complex gene sets associated with malignant evolution and poor prognosis, we intersected the differentially expressed genes (DEGs) identified via NMF subtyping with the prognostic genes from the C6 subpopulation across four large-scale, independent public GC cohorts (TCGA, ACRG, YUSH, and KUCM) using a Venn diagram. Remarkably, *limb-bud and heart (LBH)* emerged as a key candidate consistently enriched across all datasets (Fig. 2a).

We subsequently validated the expression pattern of LBH across multiple dimensions. Transcriptomic data from the public cohorts (TCGA, ACRG, KUCM, and YUSH) demonstrated a consistent and significant upregulation of *LBH* mRNA in GC tissues compared to normal tissues (TCGA, *P* < 0.001; ACRG, *P* < 0.01; KUCM, *P* < 0.01; YUSH, *P* < 0.05; Fig. 2b). This finding was further corroborated by qPCR analysis of cDNA arrays from our independent, in-house cohort (FUAPH) (FC = 2.16; *P* < 0.001; Fig. 2c, d). At the protein level, immunohistochemistry (IHC) staining and western blot (WB) assays confirmed that LBH protein expression was significantly elevated in GC tissues relative to paired adjacent normal tissues (*P* < 0.01; Fig. 2e, f).

Building upon these findings, we investigated the clinical relevance of *LBH* expression by analyzing its association with clinicopathological features and patient prognosis. Clinical correlation analyses revealed that high *LBH* expression was significantly associated with T stage (*P* < 0.01), N stage (*P* < 0.01), M stage (*P* < 0.05), and more advanced Borrmann types (*P* < 0.001; Fig. 2g), strongly implicating *LBH* in the local invasion and distant metastasis of GC. Consistently, Kaplan-Meier survival analyses across the TCGA, ACRG, KUCM, and FUAPH cohorts demonstrated that patients in the high *LBH* expression group had a significantly shorter overall survival (OS) than those in the low expression group (TCGA: *P* = 0.025; ACRG: *P* < 0.001; KUCM: *P* = 0.011; FUAPH: *P* < 0.001; Fig. 2h). Crucially, univariate and multivariate Cox regression analyses confirmed that, alongside N stage, the *LBH* expression level serves as an independent prognostic risk factor for GC patients (HR = 1.402, 95% CI: 1.097–1.792, *P* = 0.007; Fig. 2i).

Taken together, these findings identify LBH as a key regulator associated with gastric cancer progression and poor patient prognosis, prompting further investigation into its functional role in GC malignancy.

### LBH promotes gastric cancer cell proliferation in vitro and in vivo

To uncover the biological function of LBH in the malignant progression of gastric cancer (GC), we first evaluated its baseline expression across various GC cell lines. The results indicated that LBH was highly expressed in HGC27 and MKN-45 cells, but relatively lowly expressed in AGS cells (Fig. S3). Accordingly, we established LBH knockdown models in HGC27 and MKN-45 cells using short hairpin RNAs (sh-LBH) and generated an LBH overexpression (oe-LBH) model in AGS cells. The knockdown and overexpression efficiencies were confirmed at both the mRNA and protein levels (all *P* < 0.001; Fig. S4a, b). Given their superior silencing efficacy, sh-LBH#2 and sh-LBH#3 were selected for all subsequent experiments.

**Fig. 3:**
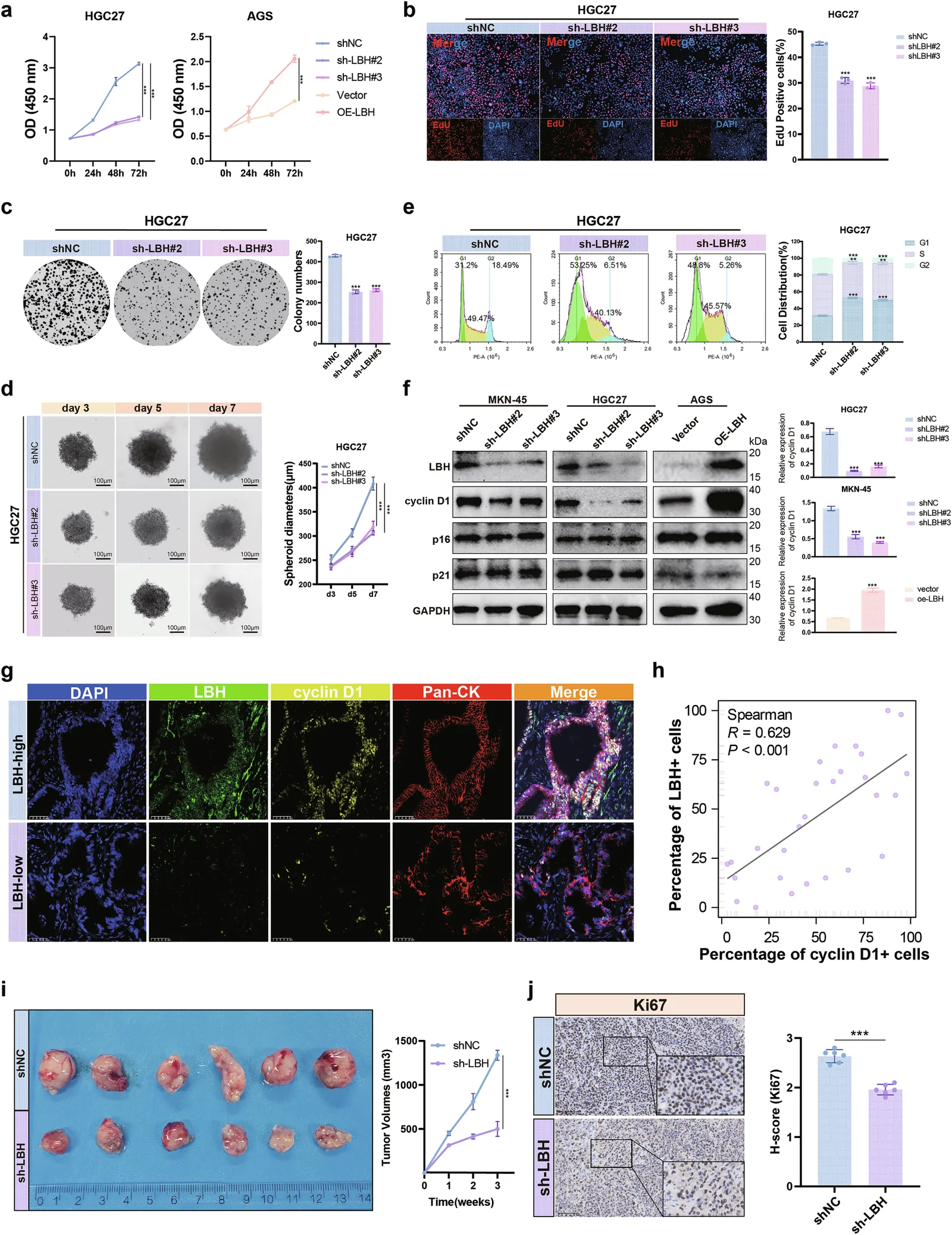
LBH promotes gastric cancer cell proliferation in vitro and in vivo by driving the G1/S transition. **a** Cell Counting Kit-8 (CCK-8) assays evaluating the in vitro proliferation of LBH-knockdown (HGC27) and LBH-overexpressing (AGS) cells. EdU incorporation (**b**) and colony formation (**c**) assays assessing the proliferative capacity of HGC27 cells following LBH knockdown. **d** Representative images and quantitative growth curves of 3D tumor spheroids derived from HGC27 cells. **e** Flow cytometry analysis of cell cycle distribution (G1, S, and G2 phases) in HGC27 cells with or without LBH knockdown. **f** Western blot analysis and corresponding quantification of cell cycle-associated proteins (LBH, Cyclin D1, p16, and p21) in LBH-knockdown (HGC27, MKN-45) and LBH-overexpressing (AGS) cells. GAPDH serves as the internal loading control. Representative multiplex immunofluorescence (mIF) images (**g**) and Spearman correlation analysis (**h**) showing the co-expression of LBH and Cyclin D1 in clinical gastric cancer tissues (*R* = 0.629, *P* < 0.001). Macroscopic images and tumor growth curves of subcutaneous xenografts (**i**), alongside representative Ki67 immunohistochemistry (IHC) staining and H-score quantification (**j**) from the shNC and sh-LBH groups. Data are presented as mean ± SD from at least three independent experiments. **P < 0.01, ***P < 0.001. CCK-8 Cell Counting Kit-8, EdU 5-ethynyl-2’-deoxyuridine, shNC short hairpin RNA negative control, mIF multiplex immunofluorescence, IHC immunohistochemistry, SD standard deviation.

Through in vitro functional assays, CCK-8, EdU incorporation, and colony formation assays consistently demonstrated that silencing LBH significantly impaired the proliferative capacity and clonogenicity of HGC27 cells (all *P* < 0.001; Fig. 3a–c). Conversely, LBH overexpression markedly augmented the proliferation of AGS cells (all *P* < 0.001; Fig. S4c–e). Furthermore, using a 3D tumor spheroid formation assay to mimic the in vivo microenvironment, we found that LBH knockdown notably reduced the maximum diameter of HGC27 spheroids, whereas LBH overexpression accelerated AGS spheroid growth (all *P* < 0.001; Figs. 3d and S4f).

Given the profound impact of LBH on cell proliferation, we investigated its effect on cell cycle distribution using flow cytometry. The results revealed that LBH knockdown induced a significant G1-phase arrest in HGC27 cells, coupled with a decreased proportion of cells entering the S phase (all *P* < 0.01; Fig. 3e). In contrast, LBH overexpression facilitated the G1/S transition in AGS cells, leading to a higher percentage of cells in the S phase (all *P* < 0.001; Fig, S4g). Mechanistically, WB analysis demonstrated that LBH knockdown dramatically downregulated the expression of cyclin D1, a core cell cycle regulator, whereas LBH overexpression upregulated cyclin D1 (all *P* < 0.001; Fig. 3f). Notably, the expression levels of the classic cell cycle inhibitors p16 and p21 remained largely unaltered (all *P* > 0.05; Figs. 3f and S4h–j). To validate this regulatory relationship in clinical settings, we performed multiplex immunofluorescence (mIF) staining on GC tissue microarrays. This confirmed a strong positive spatial correlation between LBH and cyclin D1 expression in human GC tissues (Spearman *R* = 0.629, *P* < 0.001; Fig. 3g, h).

Finally, to evaluate the oncogenic role of LBH in vivo, we established a subcutaneous xenograft mouse model (*n* = 6 per group). Compared to the shNC group, tumors derived from the LBH-knockdown group exhibited significantly reduced volumes (*P* < 0.001; Fig. 3i). IHC staining of the xenograft tissues further revealed that LBH silencing substantially decreased the expression of the proliferation marker Ki67 (*P* < 0.001; Fig. 3j). Collectively, these findings provide compelling evidence that LBH promotes GC cell proliferation both in vitro and in vivo by upregulating cyclin D1 and driving the G1/S cell cycle transition.

### LBH promotes gastric cancer cell migration, invasion, and peritoneal metastasis in vivo and in vitro by driving epithelial-mesenchymal transition

**Fig. 4:**
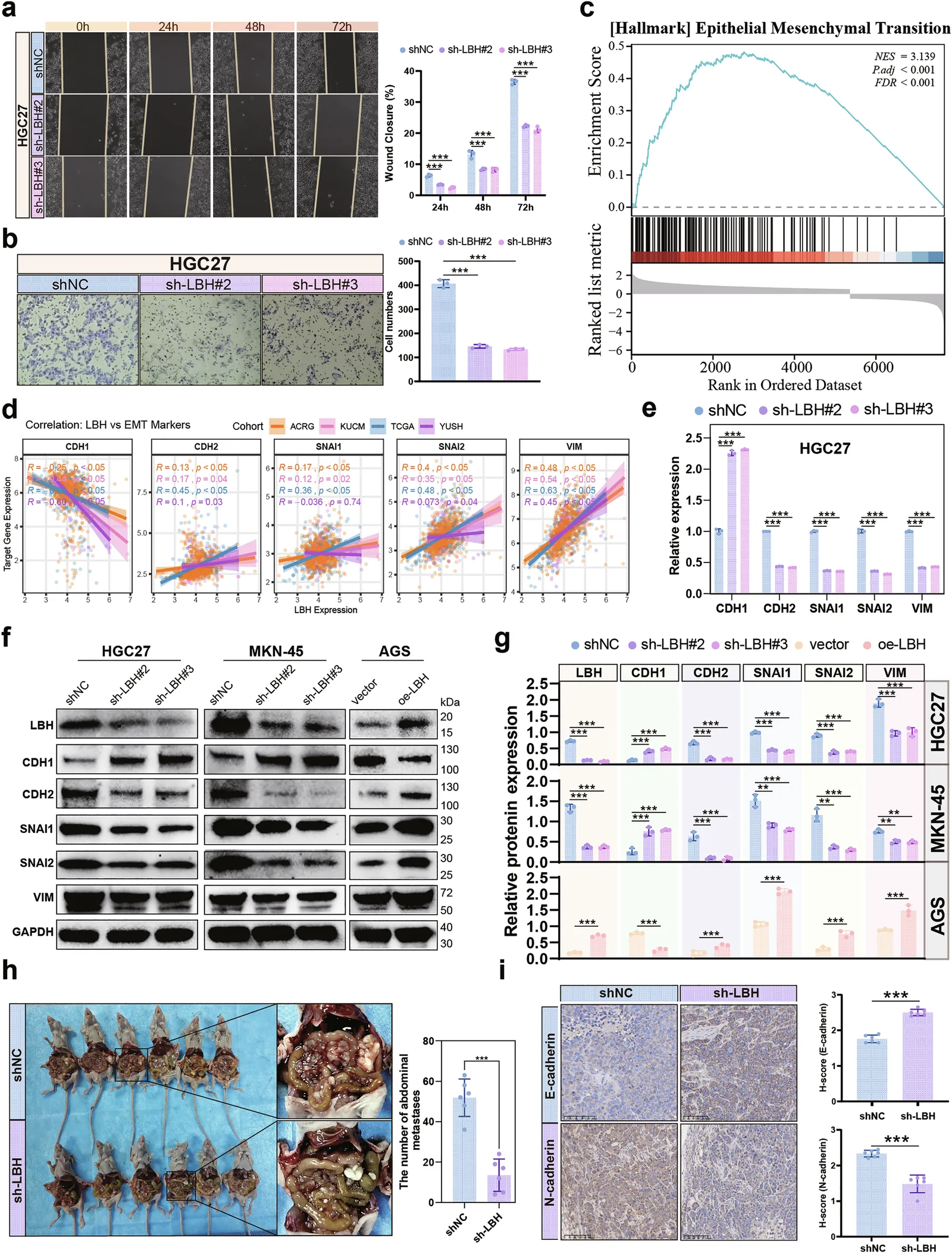
LBH promotes the migration, invasion, and epithelial-mesenchymal transition (EMT) of gastric cancer cells in vitro and tumor metastasis in vivo. **a** Representative images and wound closure quantification of HGC27 cells following LBH knockdown (sh-LBH) versus control (shNC). **b** Transwell assays and relative cell quantification evaluating HGC27 cell invasion upon LBH knockdown. **c** GSEA demonstrating positive enrichment of the hallmark EMT gene signature associated with LBH expression. **d** Correlation between LBH expression and EMT markers (*CDH1, CDH2, SNAI1, SNAI2, VIM*) across multiple gastric cancer cohorts (ACRG, KUCM, TCGA, YUSH). **e** qPCR analysis of relative mRNA levels of EMT markers in LBH-knockdown HGC27 cells. Western blot (**f**) and quantification (**g**) of LBH and EMT-related proteins (CDH1, CDH2, SNAI1, SNAI2, VIM) in LBH-knockdown (HGC27, MKN-45) and LBH-overexpressing (AGS) cells. GAPDH serves as the loading control. **h** Representative macroscopic images and statistical quantification of abdominal metastatic nodules in nude mice injected with shNC or sh-LBH cells. **i** Representative immunohistochemistry (IHC) staining and H-score quantification of E-cadherin and N-cadherin in abdominal metastatic tumors. Data are presented as mean ± SD. Statistical significance was analyzed by Student’s t-test or one-way ANOVA followed by Tukey’s multiple comparisons test. **P < 0.01, ***P < 0.001. EMT epithelial-mesenchymal transition, GSEA Gene Set Enrichment Analysis, IHC immunohistochemistry, SD standard deviation.

Given our previous observation that elevated LBH expression is closely associated with advanced clinical stages and metastatic features in GC patients, we sought to investigate its biological function in the metastatic cascade. In vitro wound healing and Transwell invasion assays demonstrated that silencing LBH significantly attenuated the migratory and invasive capacities of HGC27 cells (all *P* < 0.001; Fig. 4a, b). Conversely, exogenous overexpression of LBH substantially augmented the migration and invasion potential of AGS cells (all *P* < 0.001; Fig. S5a, b).

To elucidate the underlying molecular mechanisms by which LBH facilitates GC metastasis, we performed Gene Set Enrichment Analysis (GSEA). The results revealed a striking enrichment of the epithelial-mesenchymal transition (EMT) hallmark pathway in the *LBH*-high group (NES = 3.19; FDR < 0.001; *P* < 0.001; Fig. 4c). Further correlation analysis utilizing transcriptomic data from multiple public GC cohorts (ACRG, KUCM, TCGA, and YUSH) revealed that *LBH* expression was negatively correlated with the epithelial marker *CDH1*, but positively correlated with mesenchymal markers and core EMT transcription factors (*CDH2*, *SNAI1*, *SNAI2*, and *VIM*) (all *P* < 0.05, except for the correlation between *LBH* and *SNAI1* in the YUSH cohort, *P* = 0.74; Fig. 4d). We subsequently validated these findings in vitro. Both qPCR and WB assays consistently indicated that LBH knockdown in HGC27 and MKN-45 cells markedly upregulated the expression of the epithelial marker *CDH1* (E-cadherin), while suppressing the expression of mesenchymal markers *CDH2* (N-cadherin) and VIM, as well as the transcription factors SNAI1 and SNAI2 (all *P* < 0.001; Figs. 4e–g and S5c). Expectedly, LBH overexpression in AGS cells induced an opposite shift towards the EMT phenotype (all *P* < 0.001; Figs. 4f, g and S5d).

To corroborate the metastasis-driving role of LBH in vivo, we established a murine model of peritoneal dissemination, which closely mimics the most common route of GC metastasis. Gross anatomical examination showed that, compared to the control group (shNC), mice in the LBH-knockdown group (sh-LBH) developed significantly fewer abdominal metastatic nodules, indicating a dramatically reduced metastatic burden (*P* < 0.001; *n* = 6 per group; Fig. 4h). Moreover, IHC staining of the in vivo metastatic tissues further confirmed that LBH depletion effectively reversed the EMT process in vivo, as evidenced by the restoration of E-cadherin expression and the concurrent downregulation of N-cadherin (all *P* < 0.001; Fig. 4i). Collectively, these data convincingly demonstrate that LBH robustly promotes GC cell invasion and peritoneal metastasis both in vitro and in vivo by orchestrating the EMT program.

### LBH promotes gastric cancer cell proliferation and metastasis by activating the canonical Wnt signaling pathway

**Fig. 5:**
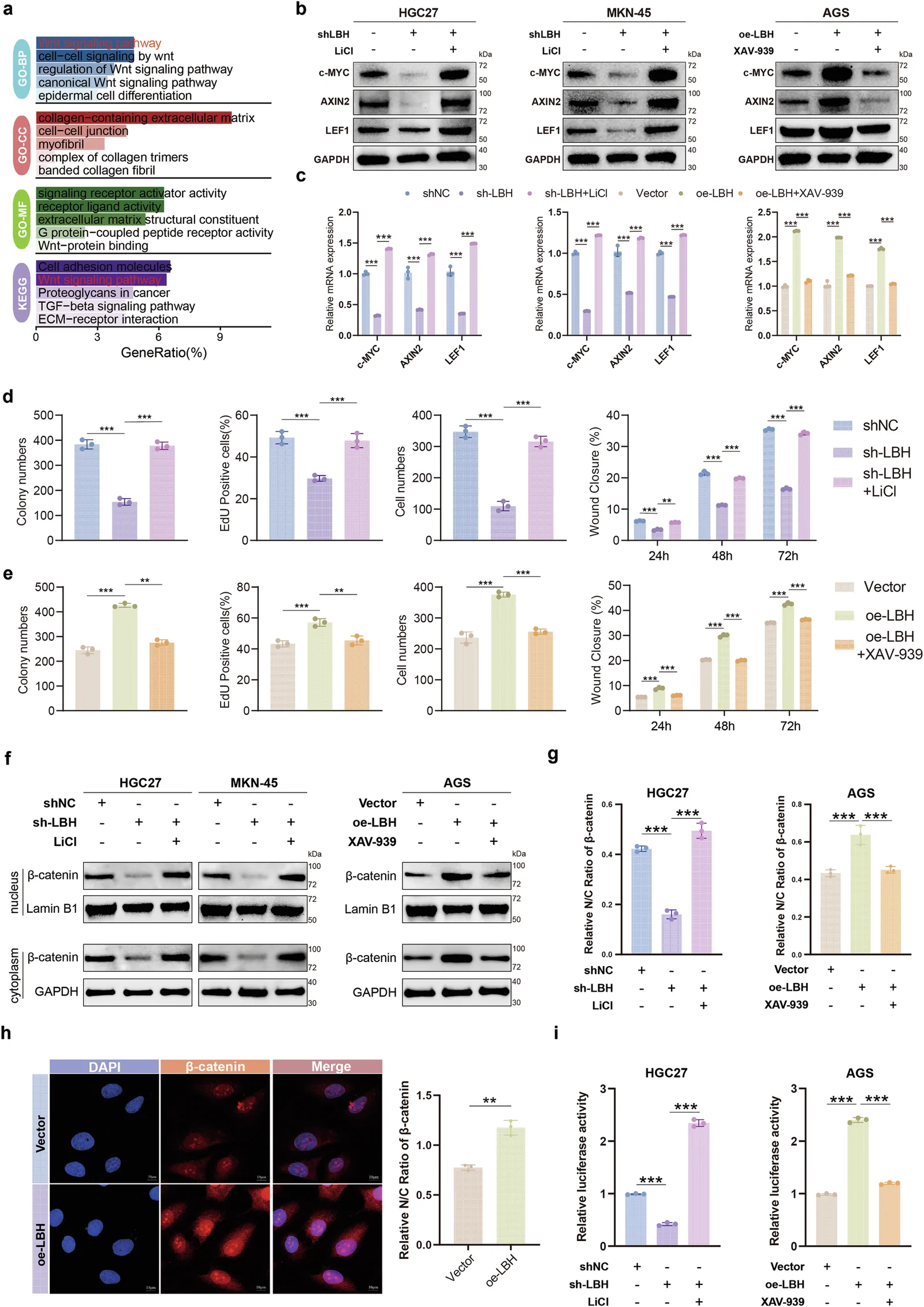
LBH promotes gastric cancer progression via the activation of the Wnt signaling pathway. **a** Gene Ontology (GO) and Kyoto Encyclopedia of Genes and Genomes (KEGG) pathway enrichment analyses based on LBH-associated differentially expressed genes. Western blot (**b**) and qPCR (**c**) analyses of canonical Wnt downstream target genes (*c-MYC, AXIN2, LEF1*) in LBH-knockdown cells (HGC27, MKN-45) treated with the Wnt agonist LiCl (10 mM, 24 h), and LBH-overexpressing cells (AGS) treated with the Wnt antagonist XAV-939 (10 μM, 24 h). GAPDH serves as the internal control. Functional rescue assays evaluating cell proliferation (colony formation and EdU), invasion (Transwell cell numbers), and migration (wound closure) in LBH-knockdown HGC27 cells treated with LiCl (10 mM, 24 h) (**d**), and LBH-overexpressing AGS cells treated with XAV-939 (10 μM, 24 h) (**e**). Western blot analysis (**f**) and corresponding quantitative analysis of the nuclear-to-cytoplasmic (N/C) ratio (**g**) demonstrating the subcellular distribution of β-catenin in the indicated cells. Lamin B1 and GAPDH served as internal loading controls for the nuclear and cytoplasmic protein fractions, respectively. **h** Representative immunofluorescence images and corresponding quantification of the N/C fluorescence ratio showing the subcellular localization of β-catenin in vector control and oe-LBH AGS cells. **i** TCF/LEF dual-luciferase reporter assays evaluating Wnt transcriptional activity in HGC27 and AGS cells under the indicated genetic and pharmacological interventions. Data are presented as mean ± SD from at least three independent experiments. Statistical significance was evaluated using one-way ANOVA followed by Tukey’s multiple comparisons test. **P < 0.01, ***P < 0.001. GO Gene Ontology, KEGG Kyoto Encyclopedia of Genes and Genomes, SD standard deviation.

To delve into the underlying molecular mechanisms by which LBH drives the malignant phenotypes of GC, we performed Gene Ontology (GO) and Kyoto Encyclopedia of Genes and Genomes (KEGG) enrichment analyses on LBH-associated differentially expressed genes. The results revealed a striking enrichment of pathways related to the canonical Wnt signaling pathway and Wnt-protein binding in the *LBH*-high phenotype (*P* < 0.001; Fig. 5a). This critical clue implicated the Wnt signaling cascade in mediating the oncogenic functions of LBH.

To validate this hypothesis, we first examined the expression dynamics of core downstream targets of the Wnt/β-catenin pathway. Consistent with the bioinformatics prediction, both WB and qPCR analyses demonstrated that LBH knockdown in HGC27 and MKN-45 cells markedly suppressed the mRNA and protein expression of target genes, including c-MYC, AXIN2, and LEF1. Conversely, LBH overexpression in AGS cells dramatically upregulated these targets (all *P* < 0.001; *n* = 3; Figs. 5b, c and S6a-c). Crucially, treatment with the Wnt pathway agonist LiCl (10 mM, 24 h) effectively reversed the downregulation of these targets induced by LBH knockdown, whereas the Wnt inhibitor XAV-939 (10 μM, 24 h) abrogated the target activation triggered by LBH overexpression (all *P* < 0.001; *n* = 3; Figs. 5b, c and S6a-c).

Building upon these molecular alterations, we subsequently conducted in vitro functional rescue assays. Phenotypically, LiCl treatment successfully rescued the impaired proliferative, migratory, and invasive capacities of LBH-depleted HGC27 cells (all *P* < 0.001; *n* = 3; Fig. 5d). Correspondingly, treatment with XAV-939 substantially abolished the proliferative and metastatic advantages conferred by LBH overexpression in AGS cells (all *P* < 0.001; *n* = 3; Fig. 5e). These compelling data indicate that the oncogenic drive of LBH in GC cells is highly dependent on the activation of the Wnt signaling pathway.

The hallmark of canonical Wnt pathway activation is the cytoplasmic stabilization and subsequent nuclear accumulation of its central transducer, β-catenin, which sequentially drives downstream transcriptional activation. Therefore, we performed subcellular fractionation followed by western blot analysis. The results revealed that LBH depletion reduced the cytoplasmic pool of β-catenin and consequently decreased its nuclear entry. Quantitative analysis confirmed a significant reduction in the nuclear-to-cytoplasmic (N/C) protein ratio, an effect that was efficiently restored by LiCl (all *P* < 0.001; Figs. 5f, g and S6d). Expectedly, LBH overexpression induced an accumulation of β-catenin in the cytoplasm, sequentially driving its increased nuclear localization and elevating the N/C ratio, which was robustly suppressed by XAV-939 (*P* < 0.001; Fig. 5f, g). Immunofluorescence (IF) staining provided complementary morphological evidence, clearly depicting enhanced nuclear accumulation in LBH-overexpressing AGS cells, accompanied by a significantly elevated N/C fluorescence intensity ratio (*P* < 0.01; Fig. 5h). To directly evaluate the impact of this accumulation on Wnt transcriptional output, we performed TCF/LEF luciferase reporter assays. Consistent with the protein dynamics, LBH knockdown substantially suppressed TCF/LEF promoter activity, whereas LBH overexpression triggered an approximately 2.4-fold increase in reporter activity (*P* < 0.001; Fig. 5i). Crucially, these transcriptional alterations were effectively reversed by LiCl and XAV-939 treatments, respectively (*P* < 0.001; Figs. 5i and S6e). Collectively, these findings explicitly demonstrate that LBH stabilizes β-catenin in the cytoplasm, facilitating its nuclear accumulation to transcriptionally activate the canonical Wnt signaling cascade.

### LBH interacts directly with β-catenin and prevents its ubiquitin-proteasomal degradation to maintain protein stability

**Fig. 6:**
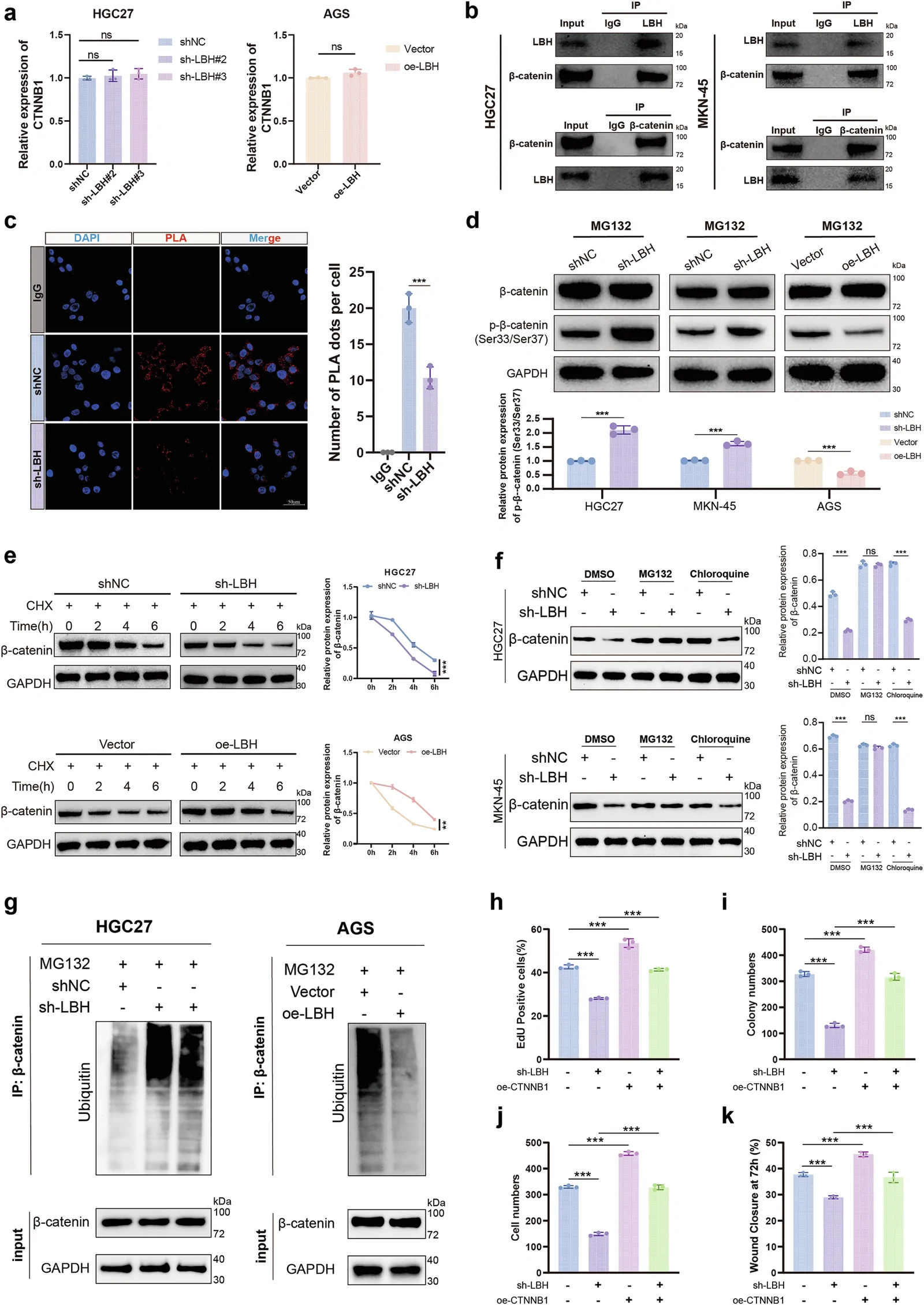
LBH physically interacts with β-catenin and prevents its ubiquitin-proteasomal degradation. **a** qPCR analysis of *CTNNB1* mRNA levels in LBH-knockdown (HGC27) and LBH-overexpressing (AGS) cells. **b** Co-immunoprecipitation (Co-IP) assays demonstrating the endogenous interaction between LBH and β-catenin in HGC27 and MKN-45 cells. **c** Representative Proximity Ligation Assay (PLA) images and corresponding quantification of PLA dots per cell, demonstrating the direct in situ physical interaction between LBH and β-catenin. IgG served as a negative control. **d** Western blot and quantitative analysis of phosphorylated β-catenin (p-β-catenin Ser33/Ser37) levels in the indicated cells pre-treated with the proteasome inhibitor MG132 (20 μM, 24 h). GAPDH serves as the internal control. **e** Cycloheximide (CHX, 50 μg/mL) chase assay and corresponding quantification assessing β-catenin protein stability in the indicated cells over 0–6 h. **f** Western blot and quantification of β-catenin protein levels in LBH-knockdown cells (HGC27, MKN-45) treated with DMSO, MG132 (proteasome inhibitor, 20 μM, 24 h), or chloroquine (lysosome inhibitor, 25 μM, 24 h). **g** Ubiquitination assays evaluating the polyubiquitination levels of β-catenin in HGC27 and AGS cells pre-treated with MG132 (20 μM, 24 h). Functional rescue assays including EdU incorporation (**h**), colony formation (**i**), Transwell invasion (**j**), and wound healing (**k**), confirming that ectopic β-catenin overexpression (oe-CTNNB1) functionally rescues the impaired proliferation, invasion, and migration induced by LBH knockdown (sh-LBH). Data are presented as mean ± SD from three independent experiments (*n* = 3). Statistical significance was determined using Student’s t-test or one-way ANOVA followed by Tukey’s multiple comparisons test. ns, not significant; **P < 0.01, ***P < 0.001. PLA Proximity Ligation Assay, Co-IP co-immunoprecipitation, CHX cycloheximide, SD standard deviation.

To elucidate the precise molecular mechanisms by which LBH modulates the canonical Wnt signaling pathway, we first investigated whether LBH regulates β-catenin at the transcriptional level. qPCR assays revealed that altering LBH expression had no significant impact on the mRNA abundance of *CTNNB1* in GC cells (all *P* > 0.05; Figs. 6a and S7a). Similarly, correlation analyses across four public GC cohorts (TCGA, ACRG, KUCM, and YUSH) demonstrated no significant correlation between the transcript levels of *LBH* and *CTNNB1* (all *P* > 0.05; Fig. S7b). Paradoxically, WB analyses clearly showed that LBH knockdown or overexpression significantly decreased or increased β-catenin protein levels, respectively (all *P* < 0.001; Fig. S7c). This discrepancy indicated that LBH regulates β-catenin via post-translational modifications rather than transcriptional control.

Consequently, we hypothesized that LBH might physically interact with β-catenin. Endogenous Co-IP assays confirmed this speculation: in both HGC27 and MKN-45 cells, specific LBH antibodies successfully pulled down β-catenin, and reciprocally, β-catenin antibodies enriched LBH (Fig. 6b). Furthermore, IF staining vividly illustrated a spatial colocalization of LBH and β-catenin at the subcellular level (Fig. S7d). To obtain definitive evidence of a direct physical interaction in situ, we performed a Proximity Ligation Assay (PLA). The PLA revealed distinct, robust red fluorescent puncta in GC cells. Crucially, the PLA signal was markedly diminished upon LBH knockdown, with no specific signal observed in the IgG negative control, collectively demonstrating the high specificity of this direct interaction (*P* < 0.001; Fig. 6c).

To further dissect the functional consequence of this physical interaction, we evaluated the impact of LBH on the primary enzymatic readout of the β-catenin destruction complex—the phosphorylation of β-catenin at Ser33/Ser37. To capture the transient phosphorylated forms, cells were pre-treated with the proteasome inhibitor MG132 (20 μM, 24 h). Strikingly, western blot and subsequent quantitative analyses demonstrated that LBH depletion significantly enhanced the specific phosphorylation of β-catenin (p-β-catenin Ser33/Ser37) whereas LBH overexpression conspicuously suppressed it (all *P* < 0.001; Fig. 6d).

Consistent with these findings, we evaluated the impact of LBH on the protein stability of β-catenin using a cycloheximide (CHX, 50 μg/mL) chase assay. The results demonstrated that LBH depletion markedly shortened the protein half-life of β-catenin in HGC27 and MKN-45 cells (*P* < 0.001; Figs. 6e and S7e), whereas LBH overexpression significantly delayed its degradation in AGS cells (*P* < 0.01; Figs. 6e and S7e). To pinpoint the specific degradation pathway, cells were treated with either a proteasome inhibitor (MG132, 20 μM, 24 h) or a lysosome inhibitor (Chloroquine, 25 μM, 24 h). Strikingly, MG132 completely reversed the downregulation of β-catenin induced by LBH knockdown (*P* > 0.05; Fig. 6f), while chloroquine failed to exert any rescue effect (all *P* < 0.001; Fig. 6f), indicating that β-catenin degradation is dependent on the ubiquitin-proteasome system (UPS). Subsequent ubiquitination assays provided definitive evidence: LBH depletion enhanced the polyubiquitination levels of β-catenin, whereas LBH overexpression attenuated its ubiquitin modifications (Figs. 6g and S7f).

Finally, to functionally validate whether β-catenin is the key downstream mediator of LBH-driven oncogenicity, we performed genetic rescue experiments. Solitary LBH knockdown significantly suppressed GC cell proliferation, clonogenicity, invasion, and migration, whereas ectopic β-catenin overexpression alone markedly enhanced these malignant phenotypes (all *P* < 0.001; Fig. 6h–k). Crucially, the reintroduction of exogenous β-catenin successfully reversed the functional inhibition caused by LBH depletion, demonstrating a definitive rescue effect (all *P* < 0.001 compared to the sh-LBH group; Fig. 6h–k). These findings definitively demonstrate that β-catenin is the primary downstream effector functionally dictated by LBH, which maintains Wnt signaling activation and drives GC progression by shielding β-catenin from ubiquitin-mediated proteasomal degradation.

### TGF-β1-induced SMAD2/3 transcriptionally activates LBH by directly binding to its promoter

**Fig. 7:**
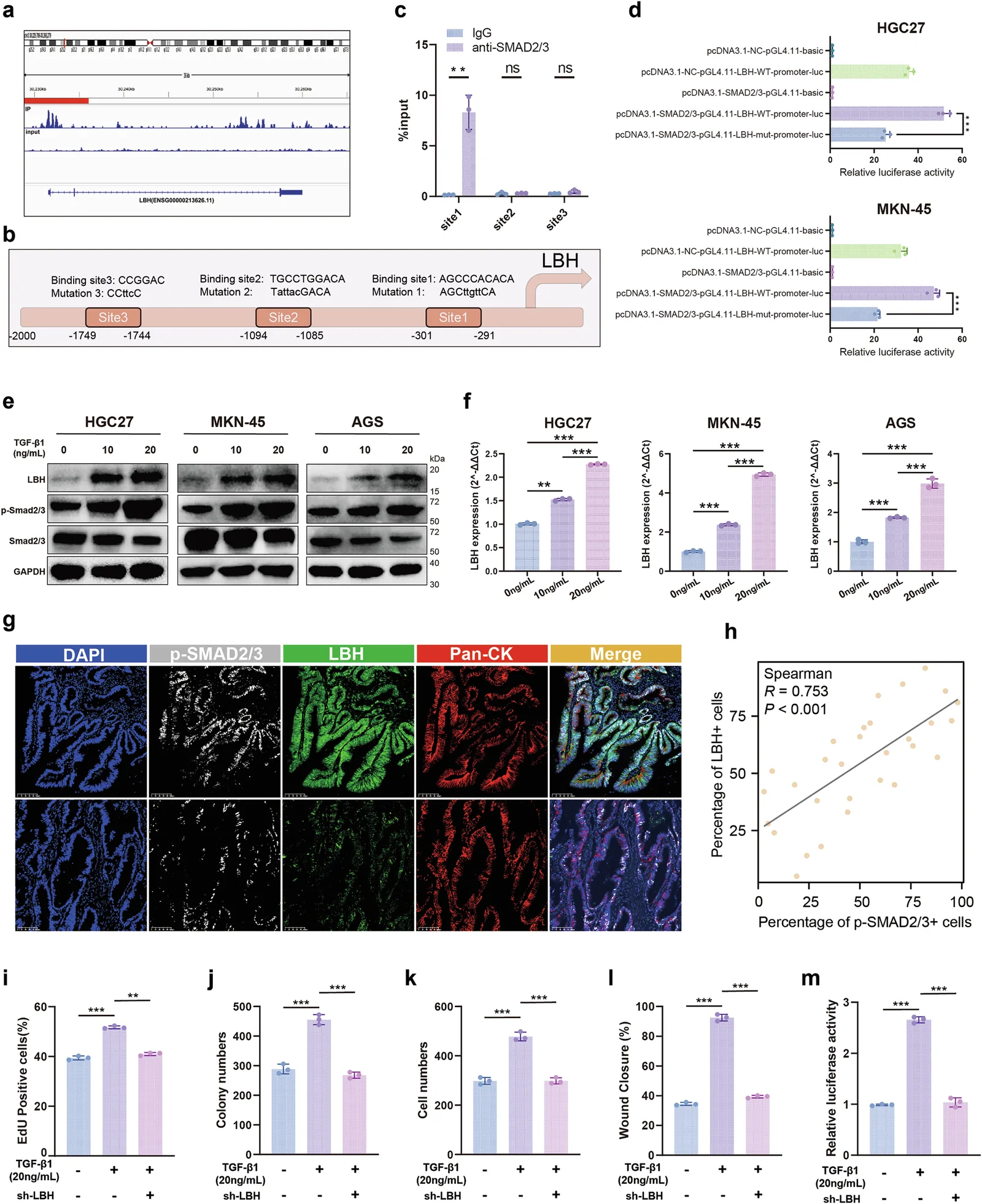
The TGF-β1/SMAD2/3 signaling pathway transcriptionally upregulates LBH to promote gastric cancer progression. Genome browser tracks of ChIP-seq data (**a**) and a schematic diagram (**b**) illustrating the predicted SMAD2/3 binding sites (and their corresponding mutant sequences) within the LBH promoter region. **c** ChIP-qPCR assays evaluating the enrichment of SMAD2/3 at the predicted binding sites (sites 1–3) of the LBH promoter. IgG served as a negative control. **d** Dual-luciferase reporter assays assessing the transcriptional activity of wild-type (WT) or mutant (mut) LBH promoters in HGC27 and MKN-45 cells co-transfected with SMAD2/3 overexpression plasmids. Western blot (**e**) and RT-qPCR (**f**) analyses of LBH and p-SMAD2/3 expression in the indicated cells (HGC27, MKN-45, AGS) treated with recombinant TGF-β1 at varied concentrations (0, 10, 20 ng/mL). Total SMAD2/3 and GAPDH serve as internal controls. Representative multiplex immunofluorescence (mIF) images (**g**) and Spearman correlation analysis (**h**) showing the co-expression of p-SMAD2/3 and LBH in clinical gastric cancer tissues. Pan-CK marks epithelial tumor cells. Functional rescue assays including EdU incorporation (**i**), colony formation (**j**), Transwell invasion (**k**), and wound healing (**l**), demonstrating that LBH knockdown (sh-LBH) attenuates the enhanced proliferation, invasion, and migration induced by TGF-β1 (20 ng/mL) treatment. **m** TCF/LEF dual-luciferase reporter assays evaluating downstream Wnt transcriptional activity following TGF-β1 (20 ng/mL) stimulation, with or without LBH knockdown. Data are presented as mean ± SD from three independent experiments (*n* = 3). Statistical significance was analyzed using Student’s t-test or one-way ANOVA followed by Tukey’s multiple comparisons test. ns, not significant; **P < 0.01, ***P < 0.001. ChIP chromatin immunoprecipitation, mIF multiplex immunofluorescence, SD standard deviation.

To comprehensively delineate the molecular mechanisms underlying the aberrant upregulation of LBH in GC, we sought to map its upstream transcriptional regulatory network. By intersecting six major transcription factor prediction databases (KnockTF, JASPAR, ChIP_Atlas, ENCODE, CHEA, and GTRD), we initially identified SMAD2 and SMAD3 as candidate upstream transcription factors (Fig. S8a). To empirically validate this prediction, our ChIP-seq data visually demonstrated substantial enrichment peaks of SMAD2/3 within the promoter region of the *LBH* gene (Fig. 7a). Subsequently, we identified three putative SMAD2/3 binding sites (Site 1–3) within the *LBH* promoter and designed corresponding mutant sequences (Fig. 7b). ChIP-qPCR analysis further pinpointed that SMAD2/3 specifically and significantly binds to site 1 (-301 to -291 bp), but not to site 2 or site 3 (site1: *P* < 0.01; site 2 and site 3: *P* > 0.05; Fig. 7c). To functionally verify the transcriptional activating effect of this direct binding, dual-luciferase reporter assays were performed. The results revealed that co-transfection with SMAD2/3 plasmids dramatically enhanced the luciferase activity of the wild-type *LBH* promoter (WT-promoter) in GC cells (HGC27, MKN-45, and AGS). Strikingly, mutation of Site 1 (mut-promoter) substantially attenuated this SMAD2/3-driven transcriptional activation (all *P* < 0.001; Figs. 7d and S8b). These data convincingly establish SMAD2/3 as the direct upstream transcriptional activators of *LBH*.

Given that SMAD2/3 are the central nuclear effectors of the TGF-β signaling pathway [20–23], we evaluated the impact of TGF-β1 stimulation on LBH expression. Correlation analyses of the TCGA and ACRG cohorts revealed a significant positive correlation between the mRNA levels of *TGFB1* and *LBH* (TCGA cohort: Spearman R = 0.658, *P* < 0.001; ACRG cohort: Spearman R = 0.304, *P* < 0.001; Fig. S8c). Crucially, in vitro assays demonstrated that treatment with recombinant TGF-β1 significantly increased p-SMAD2/3 levels and concomitantly upregulated LBH expression at both the protein and mRNA levels in a dose-dependent manner (all *P* < 0.001; Fig. 7e, f). To corroborate these findings clinically, multiplex immunofluorescence (mIF) staining of GC tissue microarrays was performed, unveiling a robust positive spatial correlation between p-SMAD2/3 and LBH expression in human GC tissues (Spearman R = 0.753, *P* < 0.001; Fig. 7g, h).

Moreover, to functionally validate that LBH serves as a critical downstream effector mediating the oncogenic effects of the TGF-β1 pathway, we performed phenotypic rescue assays. As expected, TGF-β1 stimulation (20 ng/mL) significantly enhanced the proliferative, clonogenic, invasive, and migratory capacities of HGC27 cells (all *P* < 0.01; Fig. 7i–l). Importantly, LBH silencing effectively reversed these TGF-β1-induced malignant phenotypes (all *P* < 0.01; Figs. 7i–l and S8d). Reciprocally, treatment with the specific TGF-β1 inhibitor TGF-β1-IN-1 (20 μM) significantly impaired the malignant biological behaviors of AGS cells, a suppressive effect that was robustly rescued by ectopic LBH overexpression (all *P* < 0.001; Fig. S8d–h).

To further confirm whether the TGF-β1/LBH axis ultimately drives the activation of Wnt/β-catenin signaling, we evaluated TCF/LEF promoter activity and the expression of classic Wnt target genes (c-MYC, AXIN2, LEF1). TCF/LEF luciferase activity was enhanced by exogenous TGF-β1 but substantially inhibited by LBH depletion (all *P* < 0.001; Fig. 7m). Conversely, ectopic LBH overexpression (oe-LBH) effectively bypassed upstream TGF-β inhibition (TGF-β1-IN-1) to restore this transcriptional activity (all *P* < 0.01; Fig. S8i). Consistent with these promoter activity changes, target gene expression was similarly upregulated by TGF-β1 in an LBH-dependent manner and restored by oe-LBH despite TGF-β inhibition (all *P* < 0.01; Fig. S8j). Collectively, these findings establish that elevated TGF-β1 in the tumor microenvironment drives the phosphorylation and nuclear translocation of SMAD2/3, which subsequently transactivates LBH to hyperactivate the downstream Wnt signaling axis, thereby empowering GC cells with enhanced proliferative and metastatic potential.

### FAP^+^ CAFs in the tumor microenvironment secrete TGF-β1 to drive LBH-mediated malignant progression of GC

**Fig. 8:**
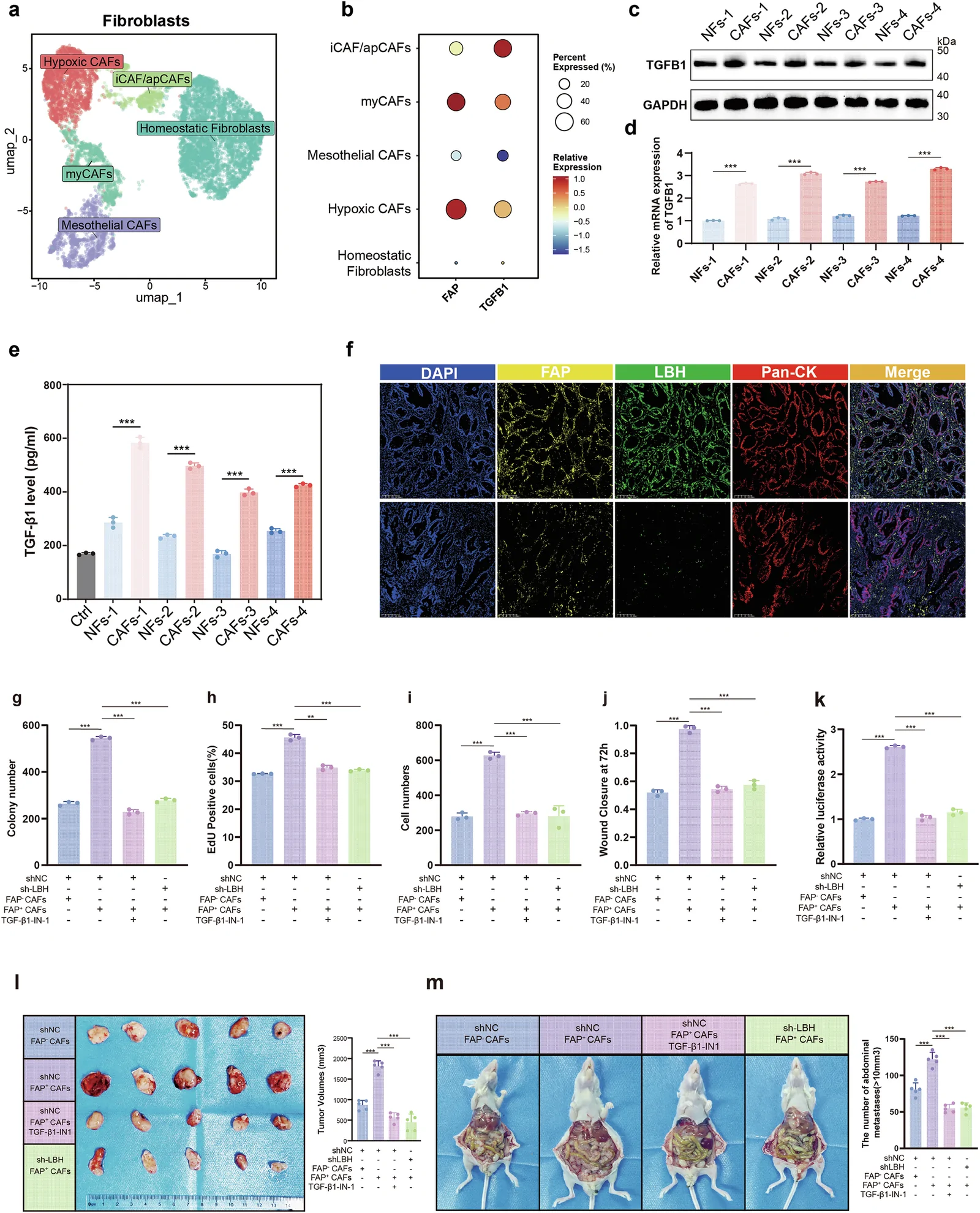
FAP^+^ CAFs secrete TGF-β1 to promote gastric cancer progression via the upregulation of LBH. UMAP embeddings (**a**) and corresponding dot plot (**b**) illustrating the identified fibroblast subpopulations and the relative expression of *FAP* and *TGFB1* genes across these subpopulations, based on single-cell RNA sequencing (scRNA-seq) data. Western blot (**c**), RT-qPCR (**d**), and ELISA (**e**) analyses evaluating TGF-β1 protein expression, *TGFB1* mRNA levels, and secreted TGF-β1 levels, respectively, in paired normal fibroblasts (NFs) and cancer-associated fibroblasts (CAFs) derived from clinical gastric cancer patients. GAPDH serves as the internal loading control for the Western blot. **f** Representative multiplex immunofluorescence (mIF) images showing the spatial distribution and proximity of FAP (CAF marker), LBH, and Pan-CK (epithelial tumor cell marker) in clinical gastric cancer tissues. In vitro functional assays including colony formation (**g**), EdU incorporation (**h**), Transwell invasion (**i**), and wound healing (**j**) evaluating the malignant phenotypes of HGC27 cells under the indicated co-culture conditions (FAP⁻ CAFs, FAP⁺ CAFs, combined with LBH knockdown or the TGF-β receptor inhibitor TGF-β1-IN-1 (20 μM)). **k** TCF/LEF dual-luciferase reporter assays evaluating downstream Wnt transcriptional activity in gastric cancer cells under the indicated co-culture and pharmacological treatment conditions. Representative macroscopic images and corresponding statistical quantification of subcutaneous tumor volumes (**l**) and abdominal metastatic nodules (**m**) in nude mice across the indicated in vivo treatment groups (TGF-β1-IN-1, 15 mg/kg/day). Data are presented as mean ± SD from three independent experiments (*n* = 3) or animal cohorts (*n* = 5). Statistical significance was analyzed using Student’s t-test or one-way ANOVA followed by Tukey’s multiple comparisons test. **P < 0.01, ***P < 0.001. UMAP Uniform Manifold Approximation and Projection, scRNA-seq single-cell RNA sequencing, mIF multiplex immunofluorescence, SD standard deviation.

To elucidate the cellular source of the elevated TGF-β1 within the GC microenvironment, we revisited scRNA-seq dataset. High-resolution sub-clustering of the fibroblast population identified five distinct functional subsets (Figs. 8a and S9a). Among the identified cancer-associated fibroblasts (CAFs), with the exception of mesothelial CAFs, the other three CAF subpopulations (namely Hypoxic CAFs, myCAFs, and iCAF/apCAFs) uniformly exhibited high co-expression of the canonical marker *FAP* and *TGFB1* (Figs. 8b and S9b). Furthermore, correlation analysis based on the fibroblast population confirmed a significant positive correlation between *FAP* and *TGFB1* expression (Spearman *R* = 0.44, *P* < 0.001; Fig. S9c). Notably, gene expression and receptor-ligand communication network (CellChat) analyses revealed that CAFs act as the primary senders of TGF-β signaling within the TME (Fig. S9d–f).

To experimentally validate these bioinformatic predictions, we isolated primary CAFs and matched normal fibroblasts (NFs) from four pairs of clinical GC and adjacent normal tissues. Subsequent qPCR, WB, and ELISA assays consistently demonstrated that both intracellular and secreted levels of TGF-β1 were significantly higher in CAFs compared to paired NFs (all *P* < 0.001; Fig. 8c–e). Importantly, mIF staining of clinical GC tissues revealed a striking spatial proximity, showing that FAP-high CAFs tightly surround LBH-high tumor epithelial cells (Pan-CK^+^) (Fig. 8f).

To functionally substantiate the FAP^+^ CAFs/TGF-β1/LBH regulatory axis, we isolated primary FAP^+^ CAFs and FAP^-^ CAFs using magnetic bead sorting, with the purity validated by flow cytometry (Fig. S9g). In vitro phenotypic assays utilizing a Transwell co-culture system demonstrated that, compared to FAP^-^ CAFs, FAP^+^ CAFs more robustly promoted the proliferation (all *P* < 0.01; Fig. 8g, h) and migration (all *P* < 0.001; Fig. 8i, j) of co-cultured GC cells. However, these FAP^+^ CAFs-conferred malignant advantages were significantly abrogated either by supplementing the system with a TGF-β1-IN-1 or by knocking down LBH in the GC cells (all *P* < 0.01; Fig. 8g–j). Furthermore, dual-luciferase reporter assays demonstrated that co-culture with FAP^+^ CAFs significantly enhanced TCF/LEF transcriptional activity in HGC27 cells, an effect that was effectively reversed by TGF-β1-IN-1 (20 μM) treatment or LBH knockdown (all *P* < 0.001; Fig. 8k). Consistent with these transcriptional changes, Western blot and subsequent densitometric analyses confirmed that FAP⁺ CAFs markedly upregulated the protein expression of classical Wnt downstream targets, including c-MYC, AXIN2, and LEF1, in the co-cultured GC cells. Notably, this stroma-induced accumulation of oncogenic targets was almost entirely abolished by either pharmacological TGF-β receptor inhibition or tumor-intrinsic LBH depletion (all *P* < 0.001; Fig. S10a, b).

Moreover, we validated this microenvironmental interaction network in vivo. In the subcutaneous xenograft model, co-injection of MKN-45 cells with FAP^+^ CAFs significantly accelerated tumor growth, which was effectively restrained by either TGF-β1-IN-1 treatment (15 mg/kg, via oral gavage, daily) or LBH knockdown (all *P* < 0.001; *n* = 5; Fig. 8l). Similarly, FAP^+^ CAFs markedly increased abdominal metastatic nodules in the peritoneal dissemination model, an effect blocked by TGF-β1-IN-1 or LBH knockdown (all *P* < 0.001; *n* = 5; Fig. 8m). IHC analysis of the xenograft tissues further elucidated the molecular basis of these phenotypic shifts (Fig. S11a–d). Specifically, FAP^+^ CAFs-driven tumors exhibited significantly higher Ki67 expression (proliferation), increased N-cadherin and β-catenin accumulation (indicating EMT induction and Wnt pathway activation), and decreased E-cadherin expression (epithelial characteristics) (all *P* < 0.01; Fig. S11a–d). Crucially, these FAP^+^ CAFs-induced molecular alterations were potently reversed by either TGF-β1-IN-1 treatment or LBH knockdown, confirming the critical role of the TGF-β1/LBH/Wnt axis in driving GC progression in vivo (all *P* < 0.01; Fig. S11a–d). Taken together, these results demonstrate that FAP^+^ CAFs in the TME continuously release TGF-β1, which activates the LBH/Wnt signaling axis in tumor cells to drive EMT-mediated metastasis and malignant proliferation.

Having established the critical role of the stromal TGF-β1/tumoral LBH axis, we next asked whether this signaling cascade operates as an autocrine loop within the CAFs themselves. Re-analysis of our scRNA-seq data revealed that while *FAP* and *CTNNB1* were highly expressed in CAF subpopulations, LBH expression was barely detectable, showing no significant correlation with FAP levels (Fig. S12a, b). In vitro validation further demonstrated that recombinant TGF-β1 stimulation failed to induce LBH expression at either the mRNA or protein level in isolated FAP^+^ CAFs (*P* > 0.05; Fig. S12c, d). Interestingly, while TGF-β1 did significantly upregulate β-catenin protein in FAP^+^ CAFs (*P* < 0.001; Fig. S12d), this upregulation occurred independently of LBH, given its inherent lack of expression in these FAP^+^ CAFs (Fig. S12d). These findings definitively demonstrate that the LBH-mediated stabilization of β-catenin is a tumor-cell-specific response to stromal TGF-β1, rather than a generalized autocrine mechanism within the tumor microenvironment.

## Discussion

GC remains a leading cause of cancer-related mortality worldwide, with peritoneal dissemination presenting a formidable clinical challenge that significantly complicates treatment and prognosis [24–26]. Despite considerable advances in understanding the molecular pathogenesis of GC, the mechanisms sustaining the persistent activation of the Wnt signaling pathway in the absence of canonical mutations remain poorly understood [27–30]. In this study, we elucidate a novel stromal–tumor crosstalk network where FAP⁺ CAFs drive the malignant progression of GC via a TGF-β1/LBH/β-catenin signaling cascade. Our findings position the transcriptional cofactor LBH as a pivotal mediator of mutation-independent Wnt activation and a promising therapeutic target.

Through integrative scRNA-seq and large-scale clinical cohort analyses, we identified *LBH* as a key gene associated with the malignant evolution of GC epithelial cells. Although a preliminary correlation between *LBH* and cancer progression has been observed in other malignancies [15, 31, 32], what distinguishes our finding is the dynamic nature of this upregulation. Notably, *LBH* expression was not universally or statically elevated across all tumor cells; instead, it was progressively activated along the pseudotime trajectory, closely correlating with EMT and Wnt signaling enrichment. This suggests that LBH actively regulates lineage plasticity, cell proliferation, and migration during malignant transformation rather than serving merely as a passive downstream biomarker. Furthermore, our extended transcriptional and in vivo profiling provided a highly concordant bridge connecting these microenvironmental dynamics to global oncogenic outputs. By employing TCF/LEF luciferase reporter assays alongside parallel expression analyses of canonical Wnt target genes (c-MYC, AXIN2, and LEF1), we demonstrated a tight coupling where both exogenous TGF-β1 and FAP⁺ CAF-driven paracrine signaling directly dictate the global Wnt transcriptional program in an LBH-dependent manner. This robust molecular activation was further substantiated in vivo by immunohistochemical H-score quantifications of xenograft tissues, which validated that the stromal-derived TGF-β1/LBH axis systematically governs tumor cell proliferation (Ki67), epithelial-mesenchymal transition (E-cadherin/N-cadherin shift), and nuclear β-catenin accumulation.

The expression and regulation of oncogenes are profoundly influenced by the TME [33, 34]. We identified FAP⁺ CAFs as a critical source of paracrine TGF-β1 [21, 35, 36], which induces SMAD2/3 phosphorylation and directly promotes the transcriptional activation of *LBH*. Intriguingly, our extended single-cell and experimental evaluations elucidated that while stromal TGF-β1 can upregulate β-catenin in FAP⁺ CAFs, this process occurs independently of LBH due to the inherent absence of LBH expression within the stromal compartment. This cell-type-specific divergence underscores that the LBH-mediated stabilization of β-catenin is strictly a tumor-intrinsic response to microenvironmental cues, rather than a generalized autocrine feedback loop within the stroma. This establishes a continuous CAF–TGF-β1–LBH–β-catenin paracrine axis that fuels GC progression. While TGF-β1 is a potent inducer of EMT and metastasis, its excessive activation in the TME contrasts with its essential role in normal tissue homeostasis. Although global pan-TGF-β or canonical Wnt inhibitors frequently trigger severe systemic toxicities due to their disruption of normal tissue homeostasis, targeting the LBH–β-catenin interface offers a dual advantage: it selectively uncouples the oncogenic paracrine communication from the stroma to adjacent epithelial tumor cells while leaving the normal stromal architecture and homeostasis untouched, thereby significantly widening the therapeutic window.

A central mechanistic breakthrough of our study addresses the paradox of persistent Wnt signaling in GC by unveiling a novel post-translational protective mechanism [37–39]. While canonical Wnt pathway activation typically relies on mutations in components such as *APC* or *CTNNB1*, these genetic alterations are infrequent in GC [38–41]. Our data resolve this discrepancy by demonstrating that LBH does not alter β-catenin at the transcriptional level; rather, it physically modulates its protein stability through direct scaffolding. In this study, we provided definitive in situ evidence using Proximity Ligation Assays (PLA) to confirm the direct physical proximity between endogenous LBH and β-catenin within the gastric cancer cell compartments. Crucially, our functional biochemistry data demonstrated that this physical interaction directly impairs the primary enzymatic readout of the β-catenin destruction complex—specifically suppressing the phosphorylation of β-catenin at Ser33/Ser37. These findings strongly support a stereospecific shielding model, wherein LBH occupies critical binding domains on β-catenin (potentially its Armadillo (ARM) repeats or N-terminal regulatory domain), creating a physical barrier that prevents GSK-3β from gaining access to and phosphorylating these key serine residues (Ser33/Ser37). Consequently, this process likely interferes with the subsequent recognition by the β-TrCP E3 ubiquitin ligase and disrupts the polyubiquitination cascade, thereby shielding β-catenin from proteasomal degradation. These findings shed new light on the mechanisms underlying mutation-independent Wnt hyperactivation in GC.

Clinically, we validated that elevated LBH expression is significantly associated with advanced clinicopathological features, including higher tumor stage, node metastasis, and peritoneal dissemination. Furthermore, multi-cohort analyses confirmed that LBH expression serves as a robust, independent prognostic risk factor for overall survival in GC patients. This clinical evidence strongly corroborates our in vitro and in vivo functional assays, providing compelling proof that targeting the LBH axis could offer a highly relevant therapeutic strategy to inhibit GC growth and metastasis. Given its central role in sustaining Wnt signaling and mediating TME crosstalk, LBH emerges as a highly actionable vulnerability in advanced GC. We have shown that the Wnt pathway inhibitor XAV-939 can counteract the oncogenic effects of LBH overexpression, supporting the premise that LBH-mediated β-catenin stabilization is indispensable for GC progression. Evaluating LBH expression levels via tumor biopsies, particularly in FAP⁺ CAF-rich microenvironments, could therefore facilitate precise patient stratification, identifying a distinct subset of advanced GC patients who are highly dependent on TME-mediated Wnt activation and would benefit most from emerging combination regimes.

Regarding the feasibility of exploiting this newly elucidated mechanism therapeutically, we acknowledge that targeting intracellular, non-enzymatic scaffold proteins has historically been challenging. However, our detailed biochemical characterization of the LBH–β-catenin interface provides viable entry points for modern drug design. The development of small-molecule inhibitors or specific blocking peptides designed to selectively disrupt this stereospecific binding interface could efficiently restore β-catenin destruction complex accessibility. Furthermore, the advent of targeted protein degradation technologies, such as proteolysis-targeting chimeras (PROTACs) [42–44], presents a highly promising frontier to completely abrogate the oncogenic scaffolding function of LBH.

We acknowledge several limitations in our current study. First, while we utilized comprehensive in vitro cell line models and in vivo mouse cohorts, the lack of patient-derived organoid (PDO) validation limits the direct extrapolation of our findings to highly complex clinical microenvironments. Second, although our PLA and biochemical assays demonstrated a direct physical interaction between LBH and β-catenin, the precise structural and mechanistic details—such as the exact binding domains—remain to be fully elucidated through future crystallography or site-directed mutagenesis studies. Finally, given the profound spatial and genetic heterogeneity of gastric cancer, the activation of the CAF-TGF-β1-LBH axis may exhibit potential variability across different GC molecular subtypes, which warrants further extensive investigation. Ultimately, evaluating the efficacy of LBH-targeted therapies, potentially in combination with localized TME modulators, in clinical trials will be critical to improving outcomes for patients with advanced GC.

## Materials and methods

### Data acquisition

A total of 114 tissue samples, comprising 94 gastric cancer (GC) tissues and 20 paired adjacent normal tissues, were collected from patients undergoing surgical resection at the Fuzhou University Affiliated Provincial Hospital (FUAPH cohort) between 2018 and 2023. This study was conducted in accordance with the Declaration of Helsinki and approved by the Ethics Committee of FUAPH; written informed consent was obtained from all participating patients. Comprehensive clinicopathological data are summarized in Supplementary Table 1.

The scRNA-seq dataset (GSE183904), generated utilizing the 10x Genomics Single Cell 5’ Library kit, was retrieved from the Gene Expression Omnibus (GEO) database [16, 45]. This cohort encompasses 40 samples: 10 normal gastric tissues (NGT), 26 GC primary tumors (GCT), 3 peritoneal metastatic tissues (PMT), and 1 normal peritoneal tissue (NPT). Detailed clinical characteristics are provided in Supplementary Table 2.

Large-scale transcriptomic and clinical profiles were integrated from The Cancer Genome Atlas (TCGA) and GEO. For the TCGA-STAD cohort, 407 initial cases (375 GC and 32 normal tissues) were screened; after excluding patients with incomplete survival data (OS ≤ 30 days), 350 GC and 32 paired normal samples were retained for downstream analysis. Furthermore, three independent GEO cohorts were included: GSE66229 (Asian Cancer Research Group, ACRG), GSE26901 (Kosin University College of Medicine, KUCM), and GSE13861 (Yonsei University Severance Hospital, YUSH) [46]. Detailed information for these cohorts is available in Supplementary Table 3.

### Data processing

For scRNA-seq data, raw data comprising an initial total of 158,641 cells were integrated using the “Seurat V5.4.0” R package, and batch effects were corrected via the “harmony” R package. Low-quality cells and putative doublets were filtered based on the following criteria: nFeature_RNA > 200, mitochondrial gene percentage <10%, and the removal of doublets using the “DoubletFinder” R package; this yielded a final cohort of 119,512 high-quality cells for downstream analysis. Dimensionality reduction and clustering analyses were performed using Seurat, with the clustering resolution parameter set to resolution = 0.5 to identify distinct subpopulations. Differentially expressed genes (DEGs) were identified using the FindAllMarkers function ( | logFC | > 1 and adjusted *P*-value < 0.05). Cell subpopulation annotation was conducted utilizing published literature, the CellMarker2.0 database, and the Cell Taxonomy database. To distinguish malignant cells from benign counterparts, chromosomal copy number variations (CNVs) were inferred using the “inferCNV” R package, with B cells serving as the reference baseline. Pseudotime, trajectory, and differentiation dynamics were inferred using the “Monocle3” R package, while intercellular communication networks were characterized via “CellChat” [47].

For bulk transcriptomic cohorts, Fragments Per Kilobase Million (FPKM) data from the TCGA-STAD cohort were log2-transformed for downstream analysis. To enable cross-cohort comparisons, the TCGA, YUSH, KUCM, and ACRG datasets were integrated. The ComBat algorithm (sva R package) was applied to eliminate inherent batch effects across different platforms.

### Cells and cell culture

The human GC cell lines (HGC27, AGS, and MKN-45) were purchased from Procell Life Science & Technology Co., Ltd. (Wuhan, China). All cell lines were authenticated via Short Tandem Repeat (STR) profiling and confirmed to be mycoplasma-free. Cancer-associated fibroblasts (CAFs) and paired normal fibroblasts (NFs) were isolated from surgical specimens of four patients diagnosed with diffuse-type GC at FUAPH. This procedure was approved by the Institutional Review Board (IRB) of FUAPH, and written informed consent was obtained prior to tissue collection. MKN-45 and HGC27 cells were maintained in RPMI-1640 medium (Gibco, USA), whereas AGS, primary CAFs, and NFs were cultured in Ham’s F-12K medium. All culture media were supplemented with 10% fetal bovine serum (FBS, Gibco) and 1% penicillin/streptomycin. Cells were incubated in a humidified atmosphere with 5% CO2 at 37 °C.

### Reagents and cell treatments

Recombinant human TGF-β1 (HY-P70543), the TGF-β receptor I kinase inhibitor TGF-β1-IN-1 (HY-151427), the Wnt/β-catenin pathway activator LiCl (HY-Y0649), the Wnt inhibitor XAV-939 (HY-15147), the proteasome inhibitor MG132 (HY-13259), and the lysosome inhibitor Chloroquine (CQ; HY-17589A) were all obtained from MedChemExpress (MCE, USA). Cells were treated with these reagents at the specified concentrations and durations as detailed in the respective figure legends prior to functional or molecular evaluations.

### Cell transfection

To establish cell lines with stable LBH knockdown or overexpression, lentiviral vectors encoding short hairpin RNA (sh-LBH) or the LBH coding sequence (oe-LBH) were synthesized by Hanheng Biotechnology (Shanghai, China). Cells were transduced with lentiviruses at an optimal multiplicity of infection (MOI) in the presence of polybrene (5 μg/mL). Stable transformants were selected using puromycin (2 μg/mL) for 14 days. Transfection efficiency was validated via RT-qPCR and Western blotting. Scrambled shRNA (shNC) and empty vectors served as negative controls. For transient CTNNB1 (β-catenin) modulation, cells were transfected with CTNNB1 overexpression plasmids (Zaigene, Fuzhou, China) utilizing Lipofectamine 3000 (Invitrogen, USA) according to the manufacturer’s protocol. Cells were harvested 48–72 h post-transfection for subsequent assays.

### qPCR

Total RNA was extracted using TRIzol reagent (Invitrogen, USA) according to the manufacturer’s protocol. RNA concentration and purity were measured using a NanoDrop 2000 spectrophotometer (Thermo Fisher Scientific). First-strand cDNA was synthesized using a reverse transcription kit (Takara, Japan). Quantitative PCR was performed using SYBR Green Master Mix on an ABI 7500 real-time PCR system (Applied Biosystems). Relative gene expression levels were calculated using the 2^−ΔΔCt^ method and normalized to GAPDH. Primer sequences are listed in Supplementary Table 4.

### Western blotting

For total protein analysis, cells were lysed in RIPA buffer supplemented with protease and phosphatase inhibitors. Nuclear and cytoplasmic proteins were isolated using the Nuclear and Cytoplasmic Protein Extraction Kit (Beyotime, Shanghai, China) following the manufacturer’s instructions. Equal amounts of protein samples were separated by SDS–PAGE and electro-transferred onto polyvinylidene fluoride (PVDF) membranes (Millipore, USA), which were pre-activated with methanol. The membranes were blocked with 5% bovine serum albumin (BSA) in Tris-buffered saline with Tween-20 (TBST; 120 mM Tris-HCl [pH 7.4], 150 mM NaCl, and 0.05% Tween-20) for 1 h at room temperature. Subsequently, the membranes were incubated overnight at 4 °C with specific primary antibodies. GAPDH was used as the loading control for total and cytoplasmic fractions, while Lamin B1 served as the internal control for the nuclear fraction. After three washes with TBST, membranes were incubated with HRP-conjugated secondary antibodies for 1 h. Protein bands were visualized using a chemiluminescent HRP substrate (Millipore, USA) and quantified via ImageJ software. Detailed information regarding the antibodies is provided in Supplementary Table 5.

### Assessment of cellular phenotypes

Cell viability and proliferation were evaluated using the CCK-8 assay and EdU incorporation assay (Beyotime, Shanghai, China) following the manufacturer’s protocols. For colony formation assays, 500 cells/well were seeded in 6-well plates and cultured for 10–14 days. Visible colonies were fixed, stained with crystal violet, and counted.

For 3D tumor sphere formation, exponentially growing cells were dissociated, centrifuged (1000 rpm, 5 min), and resuspended in complete medium. A total of 1000 cells (200 μL suspension) were seeded into ultra-low-attachment 96-well plates (Corning). Plates were centrifuged at 500 × g for 3 min to pellet the cells. Spheroids were cultured for 7-10 days and photographed daily.

Cell motility was assessed via wound-healing assays. At 90% confluence, monolayers were scratched using a sterile 200 μL pipette tip. Images were captured at 0, 24, 48, and 72 h, and wound closure rates were analyzed using ImageJ software. Transwell invasion assays were performed using 8 μm-pore inserts pre-coated with Matrigel (Corning, USA). Cells in serum-free medium were seeded in the upper chamber, while medium with 10% FBS was added to the lower chamber. After 24–48 h, invaded cells were fixed, stained, and quantified.

### Cell cycle detection

The cell cycle distribution was analyzed using flow cytometry. Cells in the logarithmic growth phase were stained with propidium iodide according to the manufacturer’s protocol and detected using a flow cytometer (Accuri C6 Plus; BD Pharmingen, Shanghai, China). Data were analyzed using the FlowJo-V10 software (Tree Star Inc., Oregon, USA).

### Isolation of primary fibroblasts, magnetic-activated cell sorting (MACS)

Fresh GC and normal tissues were minced into 1–3 mm^3^ fragments and digested with Collagenase I and hyaluronidase at 37 °C for 1–2 h. After filtration and centrifugation, cells were cultured in DMEM supplemented with 20% FBS. CAFs and NFs were initially enriched via differential adherence.

To isolate FAP^+^ subpopulations, primary CAFs were labeled with biotinylated anti-FAP antibodies and anti-biotin magnetic microbeads, then processed through MACS LS columns (Miltenyi Biotec, Germany). The purity of sorted FAP^+^ CAFs was confirmed to be >95% via flow cytometry.

### Non-contact cell co-culture

A Transwell co-culture system was utilized to evaluate intercellular crosstalk. CAFs or NFs (1 × 10⁴ cells in 1 mL complete medium) were seeded into the upper Transwell chamber, while GC cells (1–3 × 10⁵ cells) were plated in the lower chamber. For transwell assays, the setup was inverted: 1–3 × 10⁵ CAFs/NFs were seeded in the lower chamber, and 1 × 10⁴ GC cells were placed in the upper chamber. Co-cultured cells were subsequently harvested for downstream phenotypic and molecular analyses.

### Co-immunoprecipitation (Co-IP)

Total protein was extracted from cells using a specialized Co-IP lysis buffer supplemented with protease and phosphatase inhibitors. A small fraction of the cleared lysate was reserved as the “Input” control, while the remaining supernatant was incubated with 5 μg of specific primary antibodies or an equal amount of non-specific IgG (negative control) at 4 °C with gentle rotation for 4 h. Subsequently, Protein A/G magnetic beads (Thermo Fisher Scientific, USA) were added to the mixture and incubated overnight at 4 °C. The immune complexes were collected using a magnetic stand and washed three to five times with cold lysis buffer to eliminate non-specific binding. The precipitated proteins were then eluted by boiling the beads in 1× SDS loading buffer for 10 min at 100 °C, followed by Western blot analysis to detect the targeted interacting proteins.

### Ubiquitination and protein stability assays

Protein stability was assessed via a cycloheximide (CHX) chase assay. Cells were treated with 50 μg/mL CHX (Sigma-Aldrich, USA) to arrest de novo protein synthesis and harvested at 0, 2, 4, and 6 h. Target protein half-life was quantified by Western blotting. Cellular ubiquitination assays, cells were pre-treated with the proteasome inhibitor MG132 (20 μM) for 6 h. β-catenin was immunoprecipitated from lysates utilizing an anti-β-catenin antibody and Protein A/G beads. Polyubiquitination levels were subsequently evaluated by immunoblotting with an anti-ubiquitin antibody.

### Chromatin immunoprecipitation sequencing (ChIP-seq)

HGC27 cells were cross-linked with formaldehyde and sonicated to obtain chromatin with sizes ranging from 200 bp to 300 bp. Chromatin from 2 × 10⁷ cells was used in each ChIP experiment. ChIP DNA was purified using the QIAquick PCR Purification Kit (cat. no. 28104; QIAGEN) according to the manufacturer’s protocol. A ChIP DNA library was prepared using the NEBNext® Ultra™ II DNA Library Prep Kit for Illumina (cat. E7645S; NEB). Paired-end sequencing was performed using the Illumina NovaSeq platform. The sequencing results were visualized using IGV_2.19.2 software.

### Dual-luciferase reporter assay

LBH promoter activity assay: To evaluate the transcriptional regulation of LBH by SMAD2/3, the wild-type (WT) promoter sequence of LBH containing the predicted SMAD2/3 binding sites, as well as its corresponding mutant (Mut) sequence, were cloned into the pGL4.11-basic luciferase reporter vector (Promega, USA). GC cells were co-transfected with WT or Mut pGL4.11-LBH-promoter plasmids, pcDNA3.1-SMAD2/3 (or vector control), and the pRL-TK Renilla luciferase vector using Lipofectamine 3000 (Invitrogen, USA).

Wnt/β-catenin transcriptional activity assay: To assess the downstream TCF/LEF transcriptional activity, GC cells were co-transfected with the TOP-flash (containing wild-type TCF binding sites) or FOP-flash (containing mutant TCF binding sites) reporter plasmids (Millipore, USA), alongside the pRL-TK vector. After 24 h of transfection, cells were subjected to designated treatments, including recombinant TGF-β1 (20 ng/mL), TGF-β1-IN-1 (20 μM), or co-culture with CAFs.

For both assays, cells were lysed 48 h post-transfection, and luciferase activities were measured using the Dual-Luciferase Reporter Assay System (Promega, USA). Firefly luciferase activity was normalized to Renilla luciferase activity.

### Proximity ligation assay (PLA)

To visualize the direct in situ protein-protein interaction between LBH and β-catenin, the Proximity Ligation Assay was performed using the Duolink® In Situ Red Starter Kit Mouse/Rabbit (Sigma-Aldrich, USA) according to the manufacturer’s protocol. Briefly, GC cells grown on glass coverslips were fixed with 4% paraformaldehyde for 15 min and permeabilized with 0.1% Triton X-100 for 10 min. After blocking with the Duolink Blocking Solution for 1 h at 37 °C, cells were incubated overnight at 4 °C with a mixture of primary antibodies: rabbit anti-LBH and mouse anti-β-catenin. Isotype-matched IgG was used as a negative control. Next, the cells were incubated with species-specific PLA probes (PLUS and MINUS) for 1 h at 37 °C. Subsequently, ligation and amplification reactions were performed using the provided reagents to generate red fluorescent signals at the sites of interaction (distance <40 nm). Finally, the nuclei were counterstained with DAPI, and the coverslips were mounted. Images were captured using a confocal laser scanning microscope, and the number of PLA puncta per cell was quantified using ImageJ software.

### Multiplex immunofluorescent staining

Multiplex immunofluorescence staining was performed using the Opal Multiplex Staining Kit (Akoya Biosciences), according to the manufacturer’s instructions. The tissue microarrays were dewaxed, rehydrated, and fixed with 10% paraformaldehyde for 20 min. After washing with TBST, cells were treated with AR6 buffer (Akoya Biosciences) under microwave irradiation. After cooling to 25 °C, the arrays were washed with TBST and blocked with the Opal antibody diluent for 10 min. The microarrays were probed overnight with primary antibodies at 4 °C and washed. After washing, the microarrays were incubated with horseradish peroxidase-conjugated secondary antibodies at room temperature for 10 min and washed. After washing, the microarrays were incubated with Opal dye (1:100 dilution) for 10 min. This was followed by washing and a microwave treatment. Finally, the nuclei were stained with DAPI (Akoya Biosciences), and the slides were mounted with Abcam mounting media.

### Immunohistochemistry

Immunohistochemistry was performed on 4-μm-thick paraffin-embedded tumor sections. Sections were baked at 60 °C for 1 h, dewaxed in xylene, and rehydrated through a graded ethanol series. Endogenous peroxidase activity was quenched with 3% (v/v) hydrogen peroxide for 12 min. Antigen retrieval was performed by immersing the sections in 0.01 mol/L citrate buffer (pH 6.0) under microwave irradiation for 30 min. After blocking with 5% BSA for 1 h, the sections were incubated overnight at 4 °C in a humidified chamber with specific primary antibodies against Ki67, E-cadherin, N-cadherin, and β-catenin. Following incubation with HRP-conjugated secondary antibodies, the signals were visualized using a DAB substrate kit, and nuclei were counterstained with hematoxylin.

The staining expression was semi-quantitatively evaluated using the Histochemistry Score (H-score) method. The H-score was calculated based on the proportion of positively stained cells and the staining intensity. The intensity was graded as 0 (negative), 1 (weak), 2 (moderate), and 3 (strong). The H-score was calculated using the following formula: H-score = ∑ (PI × I) = (percentage of cells of weak intensity × 1) + (percentage of cells of moderate intensity × 2) + (percentage of cells of strong intensity × 3), yielding a score ranging from 0 to 300. Three randomly selected high-power fields were evaluated per section by two independent pathologists blinded to the experimental groups.

### Animal experiments

Specific-pathogen-free Balb/C nude mice (4-6 weeks old) were utilized for in vivo models. Mice were randomly allocated to different experimental groups. All procedures complied with the Fujian Provincial Regulations on Animal Welfare and were reported according to ARRIVE guidelines. For subcutaneous xenografts, 5 × 10⁶ MKN-45 cells (alone or mixed with FAP^+^ CAFs at a 2:1 ratio) were resuspended in 100 μL PBS/Matrigel (1:1) and injected into the right dorsal flank. Once tumors reached 100 mm³, mice received either TGF-β1-IN-1 (15 mg/kg, via oral gavage, daily) or vehicle control. Tumor dimensions were measured every 3 days, and volume was calculated as: *V* = *(length×width*^*2*^*) / 2*.

For the peritoneal metastasis model, 2 × 10⁶ MKN-45 cells and CAFs (2:1 ratio) in 100 μL PBS were injected intraperitoneally. TGF-β1-IN-1 treatment commenced one week post-injection. Humane endpoints were enforced; mice were euthanized if subcutaneous tumors exceeded 2000 mm³, or if severe ascites/ulceration occurred. At experimental endpoints (day 28 for xenografts, week 4 for peritoneal models), tissues were harvested, weighed, and macroscopic metastatic nodules ( > 3 mm) were quantified. Tissues were snap-frozen or fixed in 4% paraformaldehyde for downstream analysis.

### Statistical analysis

Data from at least three independent biological replicates are presented as mean ± standard deviation (SD). Statistical analyses were performed using R software (version 4.5.0) and GraphPad Prism (version 9.0). Two-group comparisons were evaluated via two-tailed Student’s *t*-tests, while multi-group comparisons utilized one-way ANOVA followed by Tukey’s post-hoc test. For survival analysis, patients were stratified by median LBH expression; Kaplan-Meier curves were compared via the log-rank test. Univariate and multivariate Cox proportional hazards models identified independent prognostic factors. Correlations between tissue markers were assessed using Spearman’s rank correlation. Statistical significance was defined as *P* < 0.05 (ns: *P* > 0.05; *P < 0.05; **P < 0.01; ***P < 0.001).

## Conclusion

In conclusion, our study identifies LBH as a critical oncogenic driver, an independent prognostic factor, and a distinct therapeutic vulnerability in gastric cancer. Mechanistically, we elucidate a novel post-translational protective mechanism whereby LBH physically interacts with β-catenin in situ, stereospecifically shielding it from destruction complex-mediated phosphorylation and subsequent ubiquitin-proteasomal degradation. This scaffolding function sustains mutation-independent Wnt/β-catenin pathway activation to promote tumor proliferation and EMT-mediated metastasis. Furthermore, we demonstrate that this LBH-dependent stabilization program is selectively activated in tumor cells by FAP⁺ CAFs-derived TGF-β1 via the SMAD2/3 cascade. Collectively, these findings uncover a novel paracrine FAP⁺ CAF/TGF-β1/LBH/β-catenin signaling axis in the tumor microenvironment, highlighting the targeted disruption of the LBH–β-catenin interface as a highly promising therapeutic strategy to uncouple tumor-stroma crosstalk and suppress advanced GC.

## Supplementary information

Supplementary Materials

Supplementary Tables

Original Data

## Data Availability

All the data used and analysed in this study are available within the article and its supplementary sources. The publicly available datasets can be found here: TCGA (http://portal.gdc.cancer.gov/) and GEO (accession numbers: GSE183904, GSE66229, GSE26899, and GSE13861). The underlying custom code for this study is provided as part of the Supplementary Material.
