# Supplementary material for: CAF-derived TGF-β1 activates an LBH-mediated β-catenin stabilization program to drive gastric cancer progression
Source: Cell Death Dis. 2026 Aug 6;17(1):935. doi: 10.1038/s41419-026-09150-w (PMC13731647; doi:10.1038/s41419-026-09150-w)

Figure 2 f

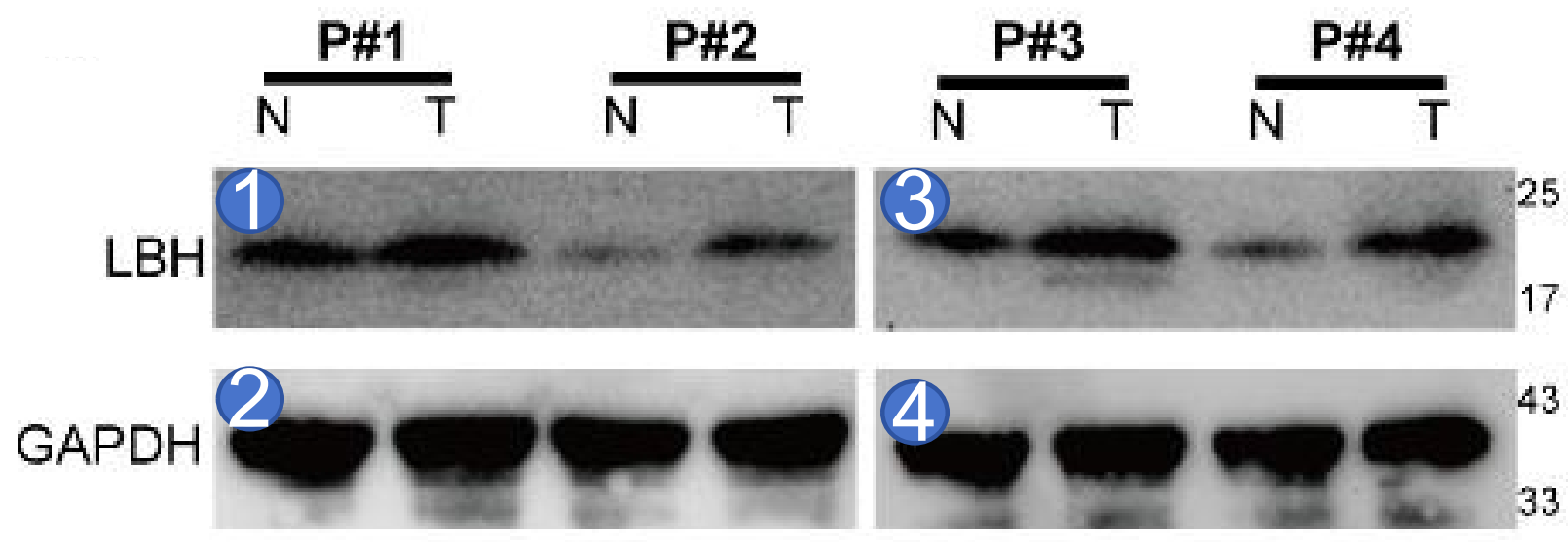

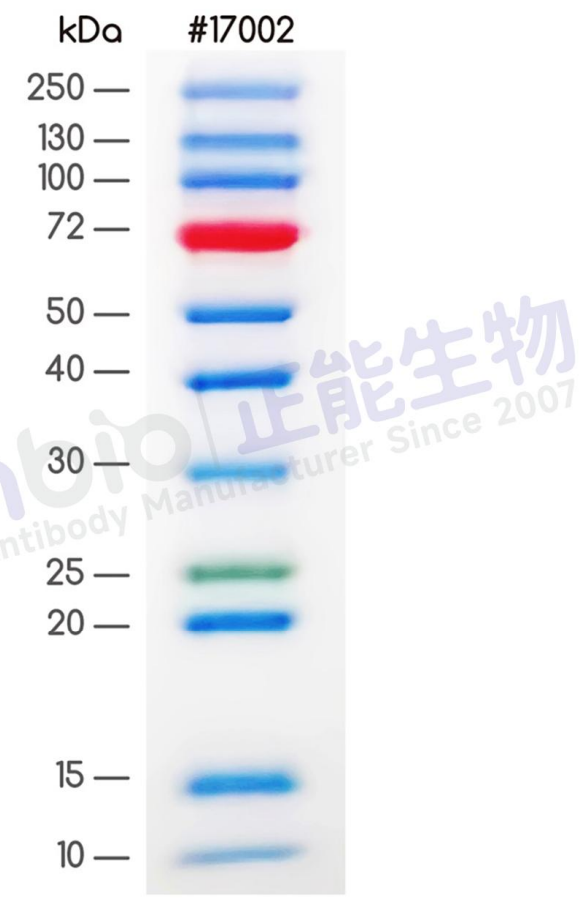

Copyright© 2025 zen-bioscience Limited.

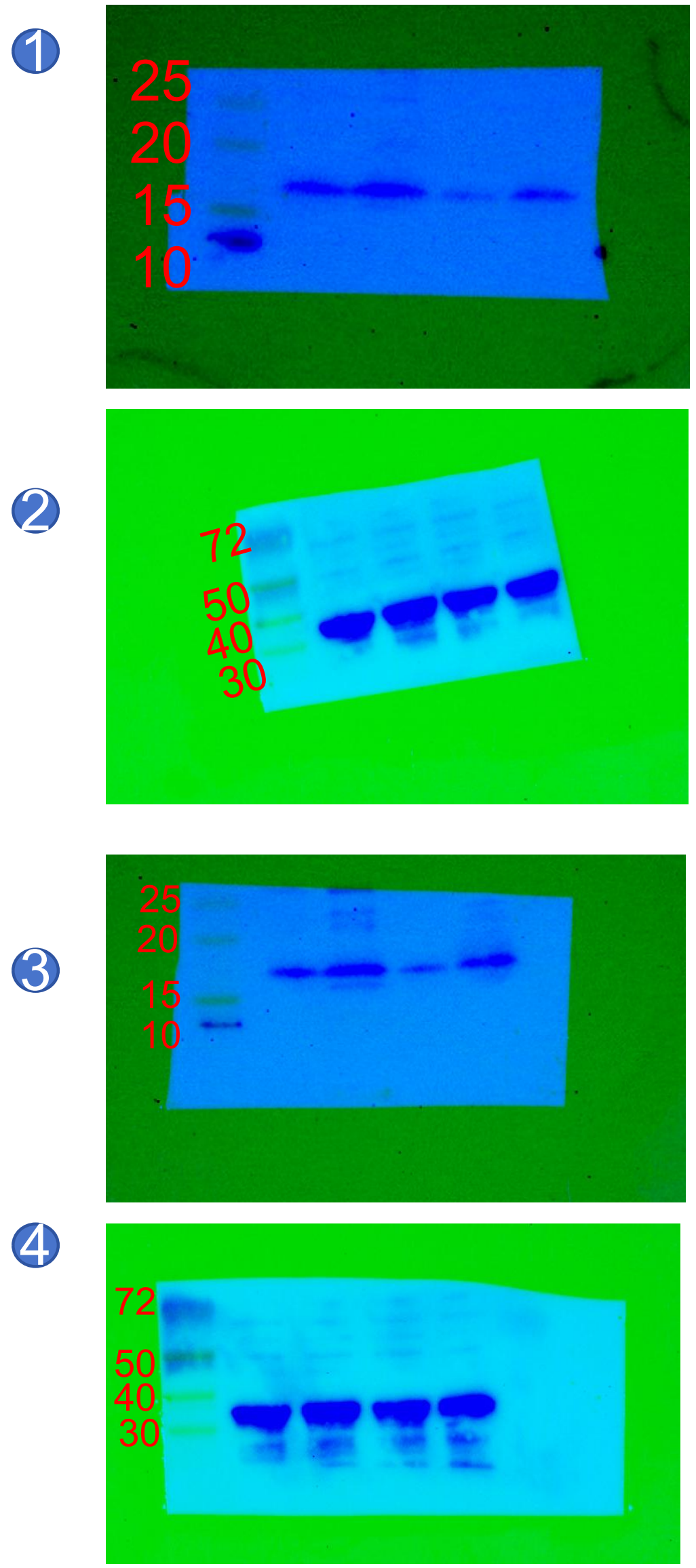

Figure 3f

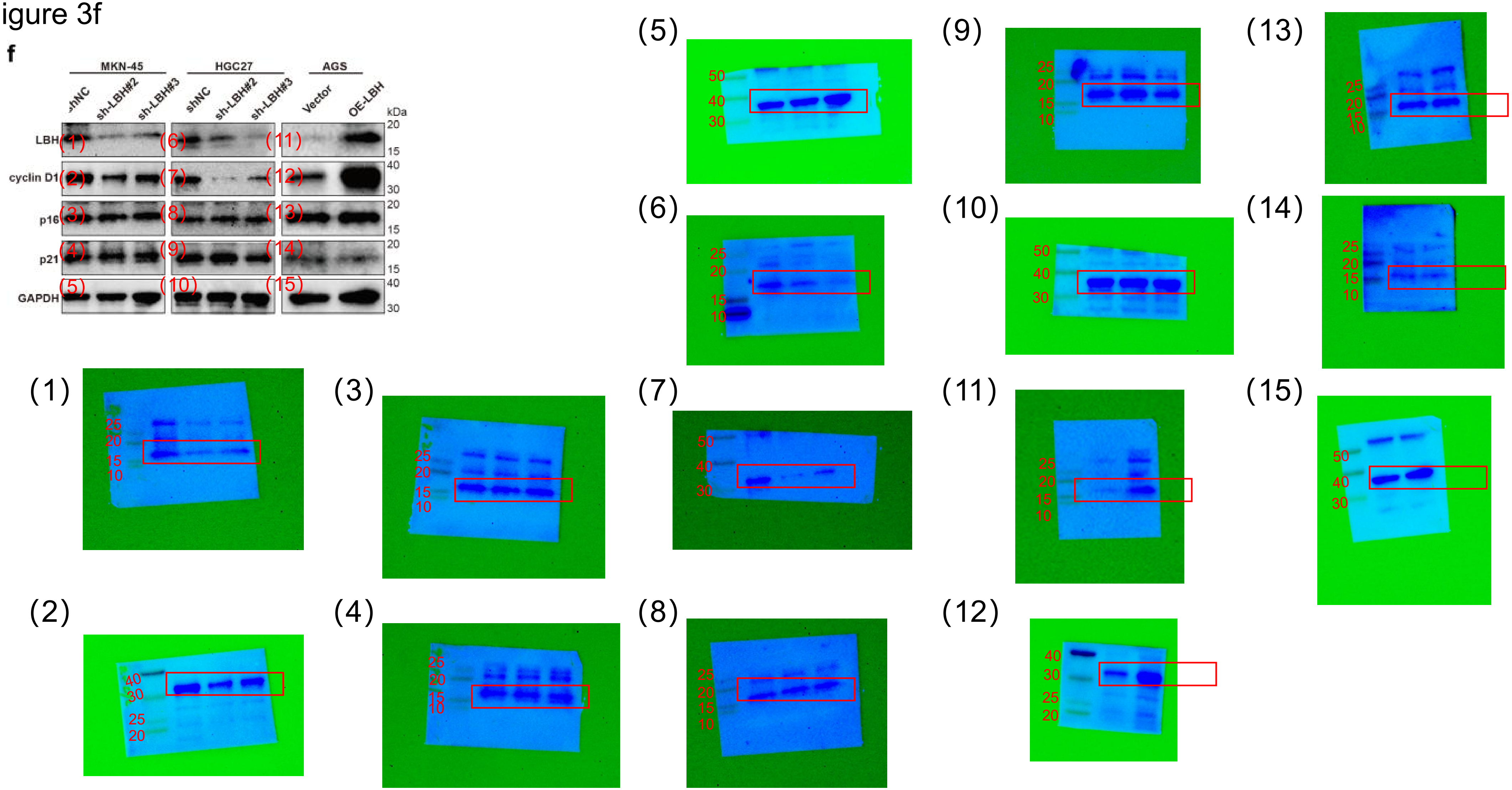

f
MKN-45
HGC27
AGS
shNC
sh-LBH#2
sh-LBH#3
shNC
sh-LBH#2
sh-LBH#3
Vector
OE-LBH
kDa
LBH
cyclin D1
p16
p21
GAPDH
(1)
(2)
(3)
(4)
(5)
(6)
(7)
(8)
(9)
(10)
(11)
(12)
(13)
(14)
(15)

Figure 4f-1

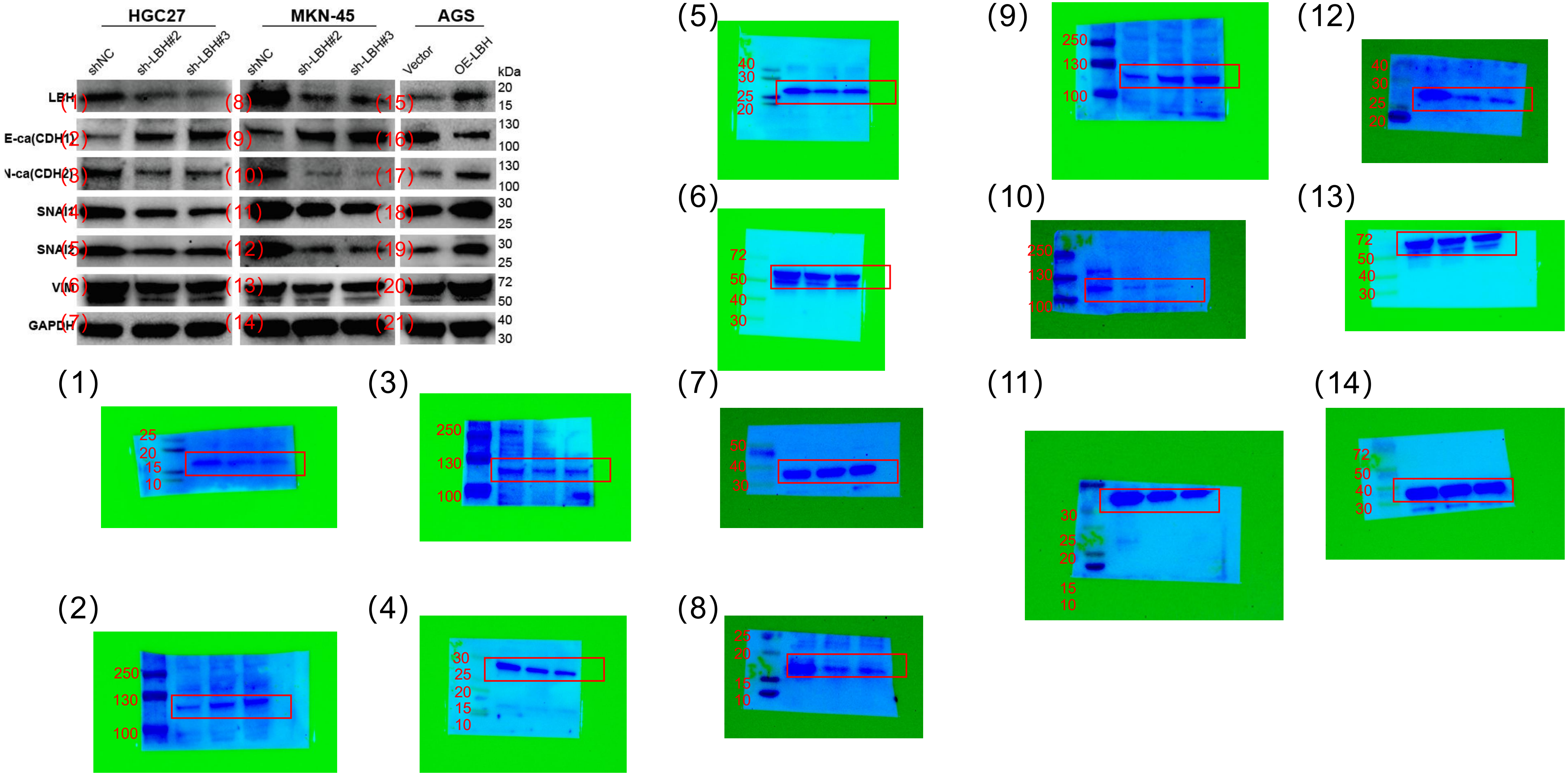
HGC27
MKN-45
AGS
shNC
sh-LBH#2
sh-LBH#3
Vector
OE-LBH
kDa
LBH
E-ca(CDH1)
N-ca(CDH2)
SNAI1
SNAI2
VIM
GAPDH
(1)
(2)
(3)
(4)
(5)
(6)
(7)
(8)
(9)
(10)
(11)
(12)
(13)
(14)

Figure 4f-2

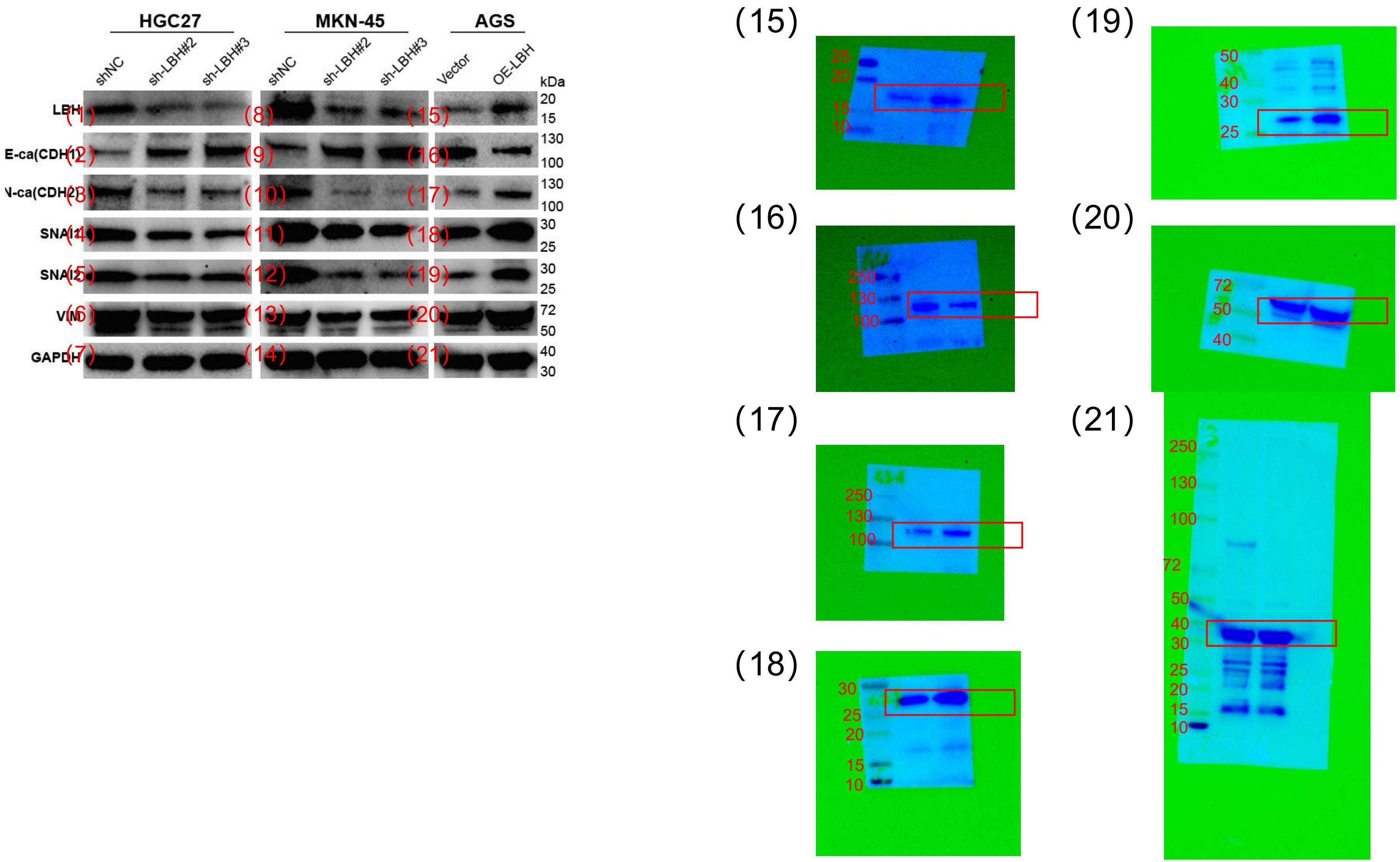
HGC27
MKN-45
AGS
shNC
sh-LBH#2
sh-LBH#3
shNC
sh-LBH#2
sh-LBH#3
Vector
OE-LBH
kDa
LBH
E-ca(CDH1)
N-ca(CDH2)
SNAI1
SNAI2
VIM
GAPDH
(15)
(16)
(17)
(18)
(19)
(20)
(21)

Figure 5 b

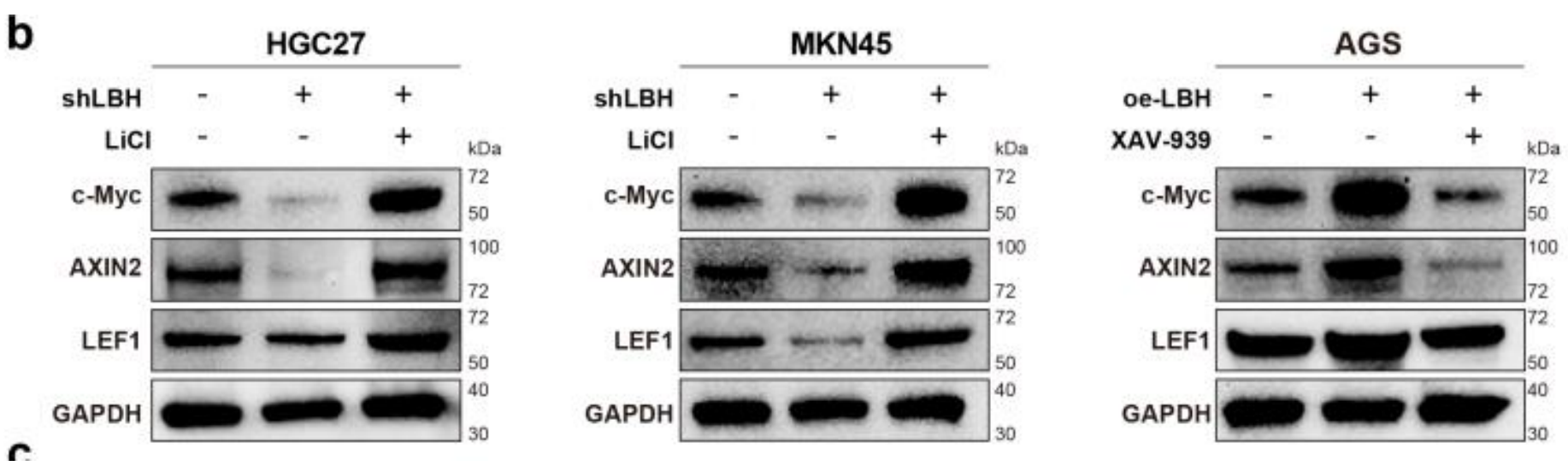
b
HGC27
shLBH - + +
LiCl - - +
MKN45
shLBH - + +
LiCl - - +
AGS
oe-LBH - + +
XAV-939 - - +
kDa
c-Myc
AXIN2
LEF1
GAPDH
72
50
100
72
72
50
40
30

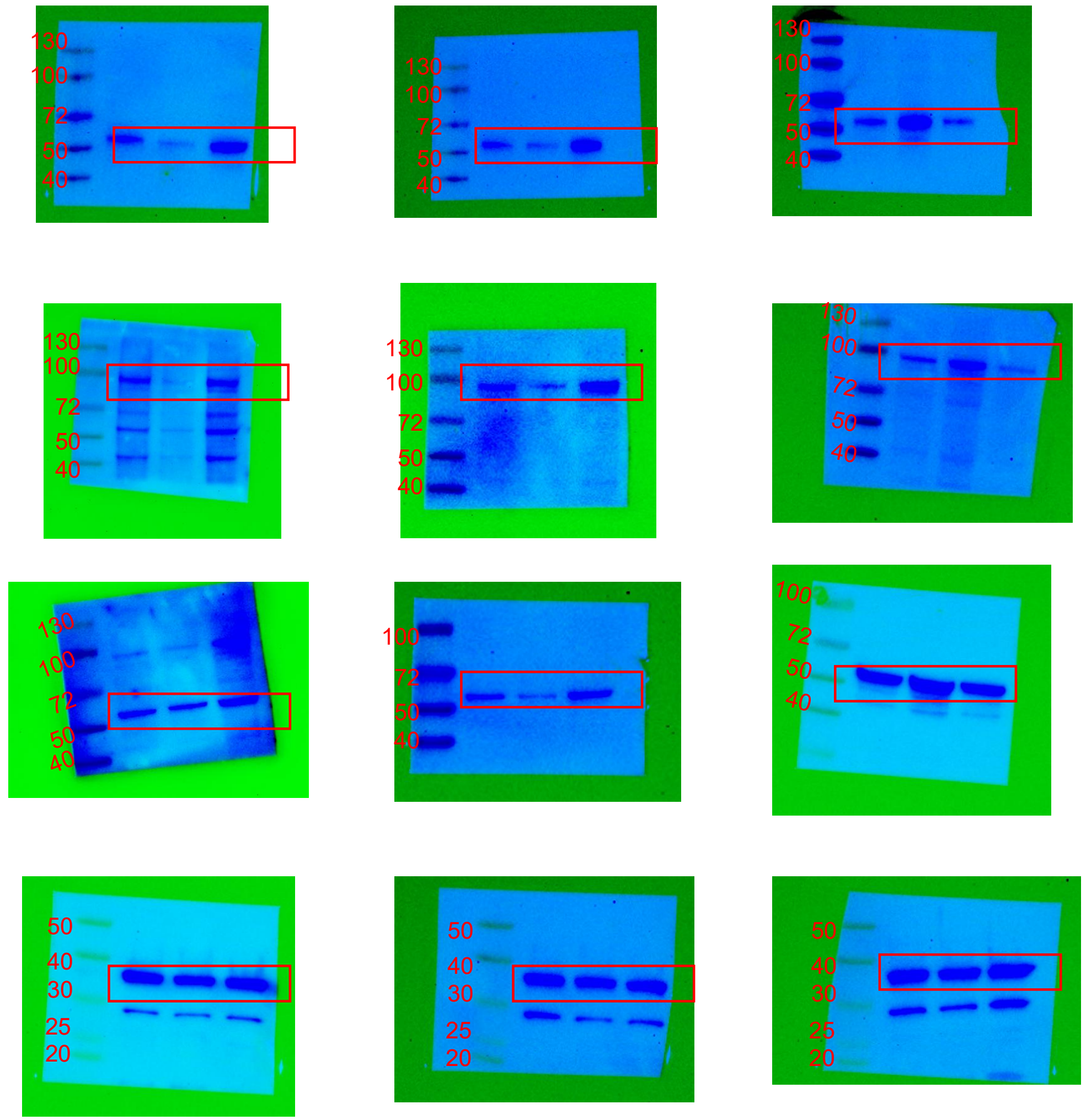
130
100
72
50
40
25
30
20

Figure 5 f

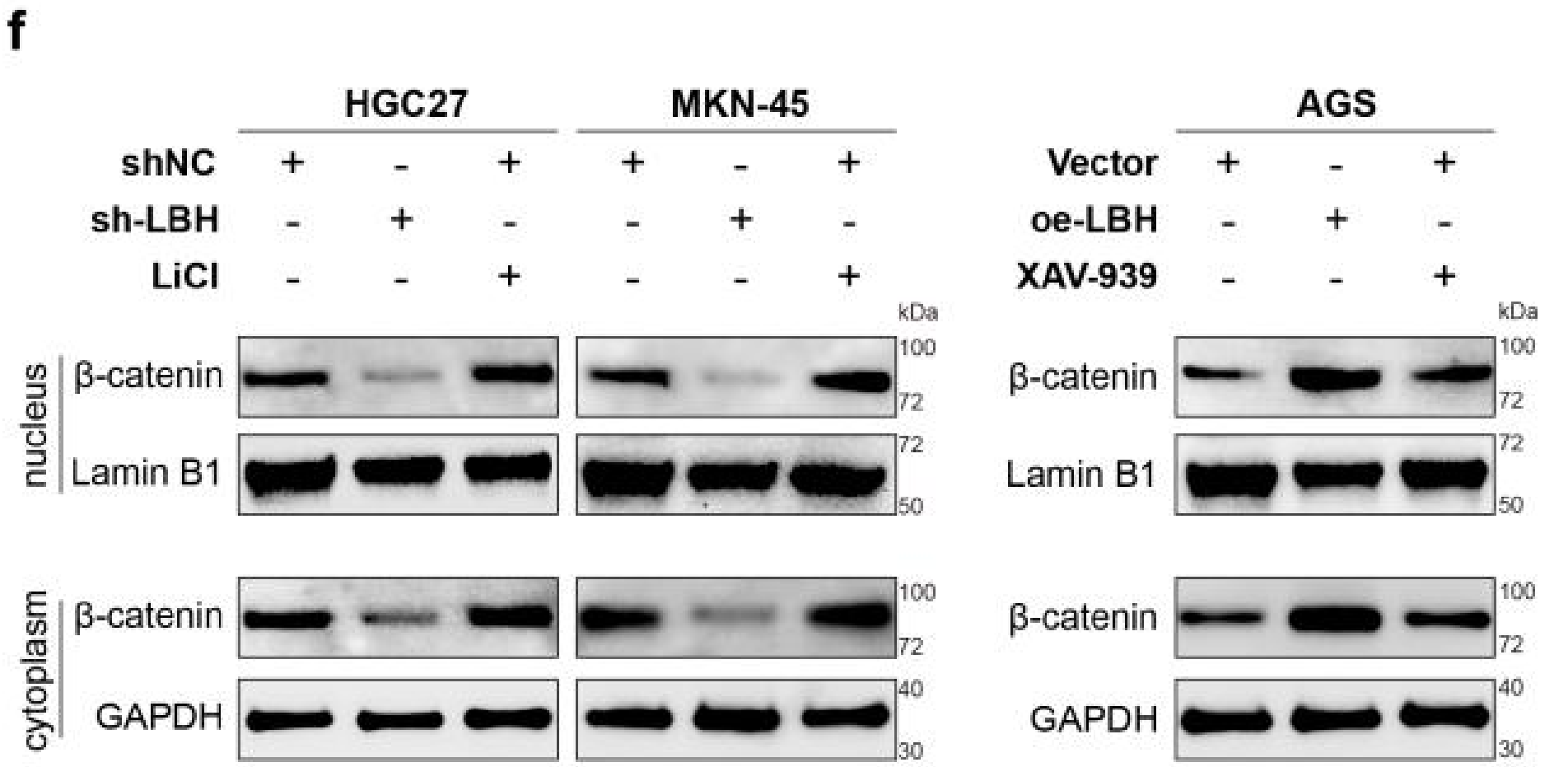
f
HGC27
MKN-45
AGS
shNC
sh-LBH
LiCl
Vector
oe-LBH
XAV-939
kDa
nucleus
β-catenin
Lamin B1
cytoplasm
β-catenin
GAPDH
100
72
50
40
30

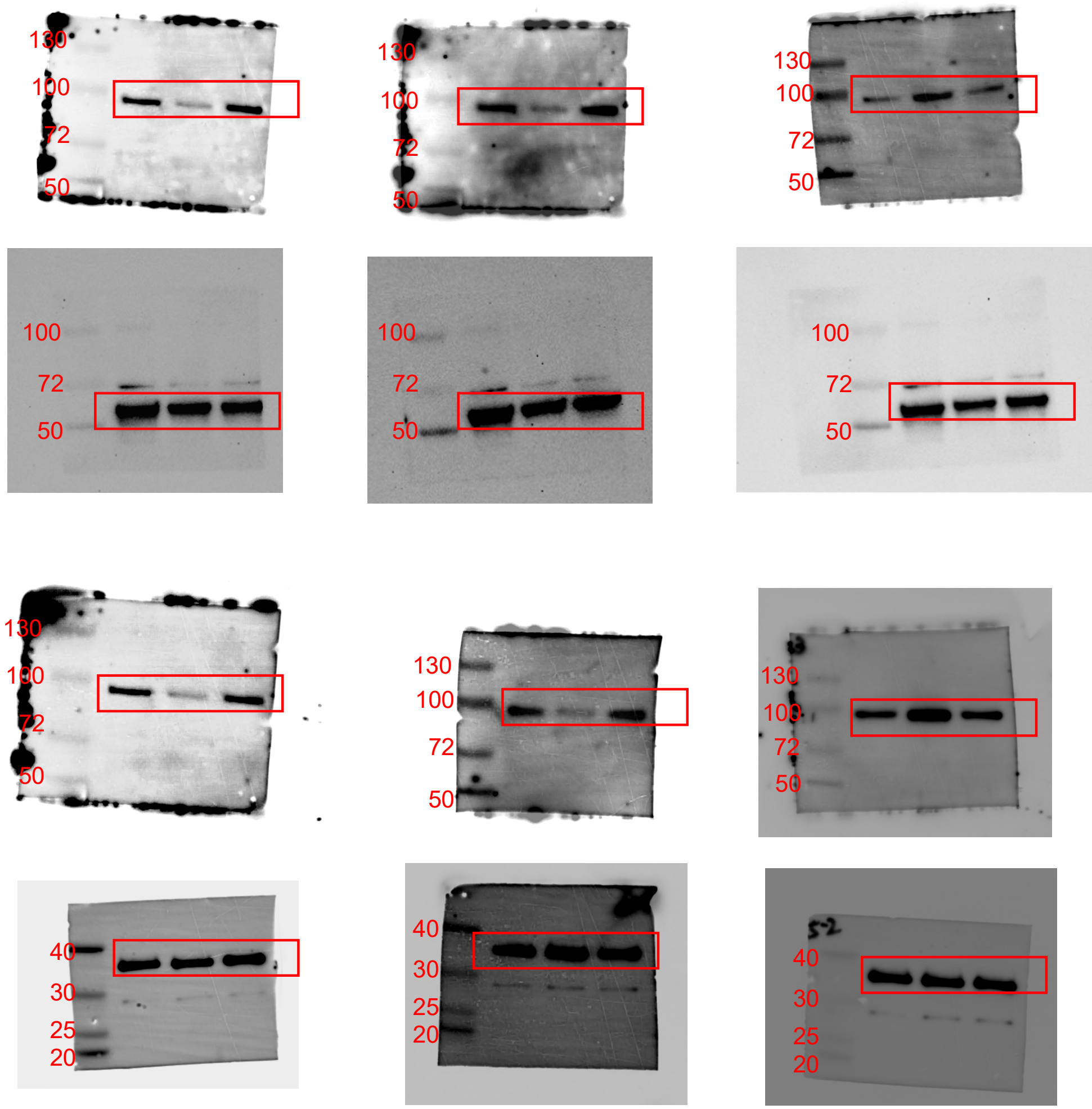
130
100
72
50
40
30
25
20

Figure 6b

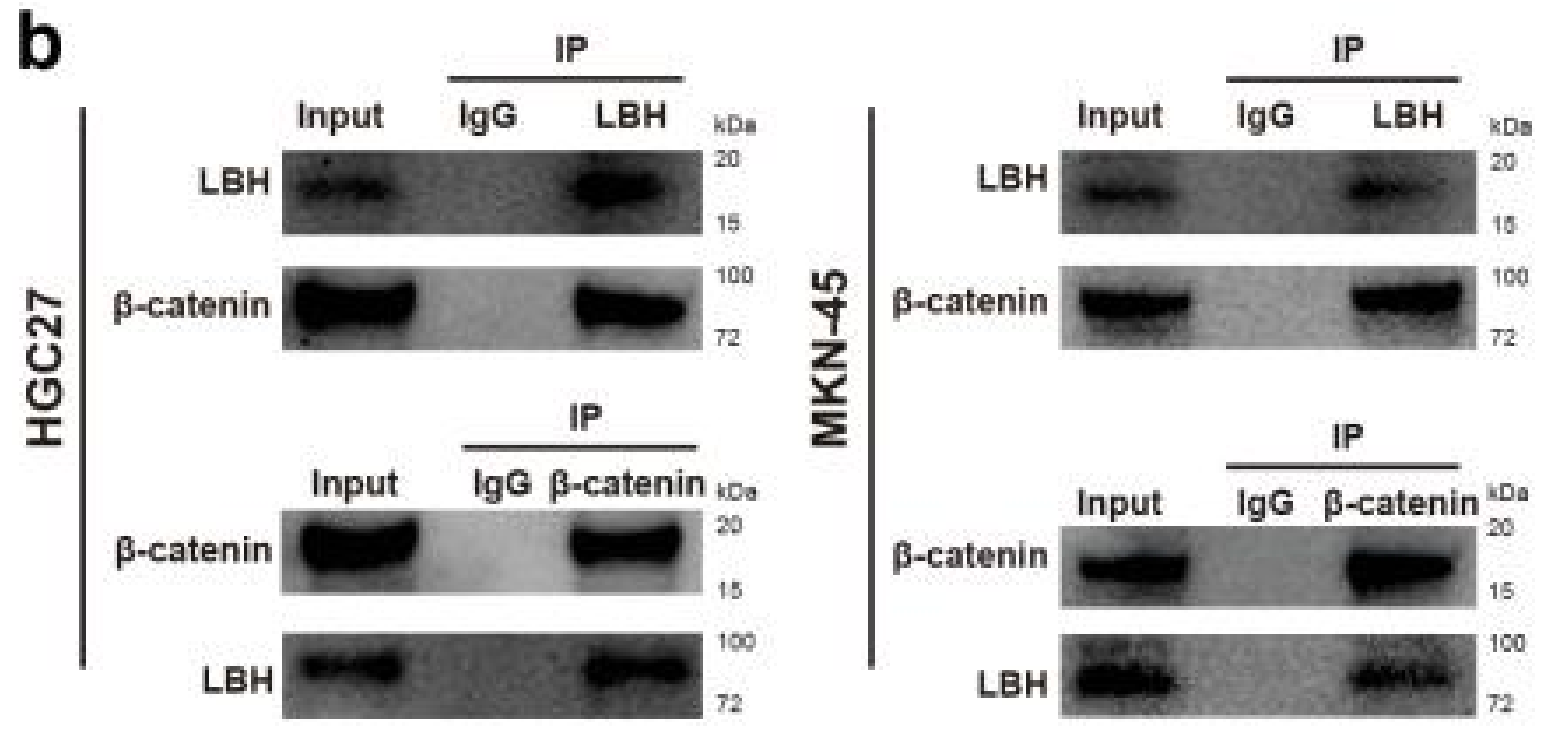
b
IP
Input
IgG
LBH
kDa
LBH
20
15
β-catenin
100
72
HGC27
Input
IgG
β-catenin
β-catenin
LBH
MKN-45

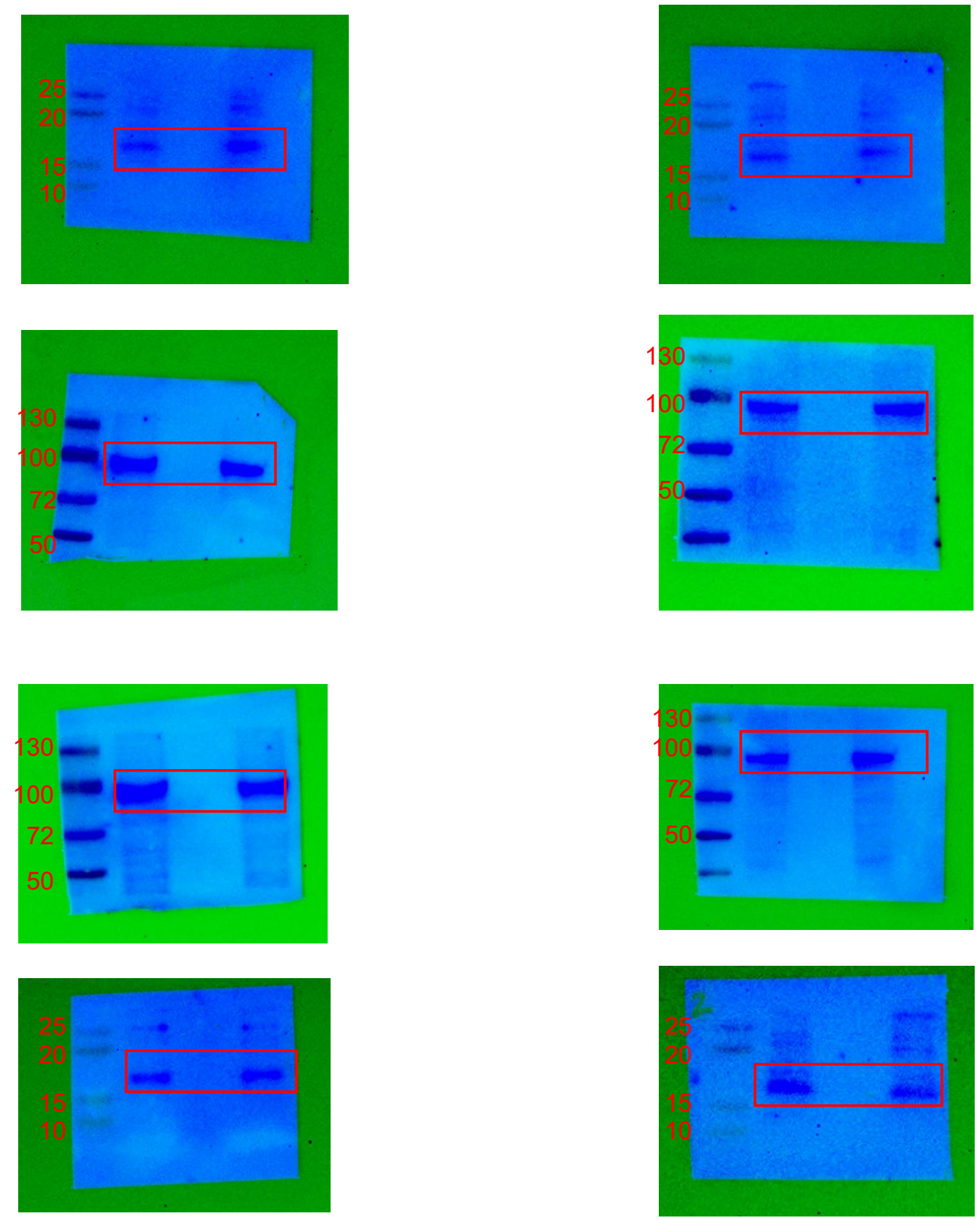
25
20
15
10
130
100
72
50

Figure 6d

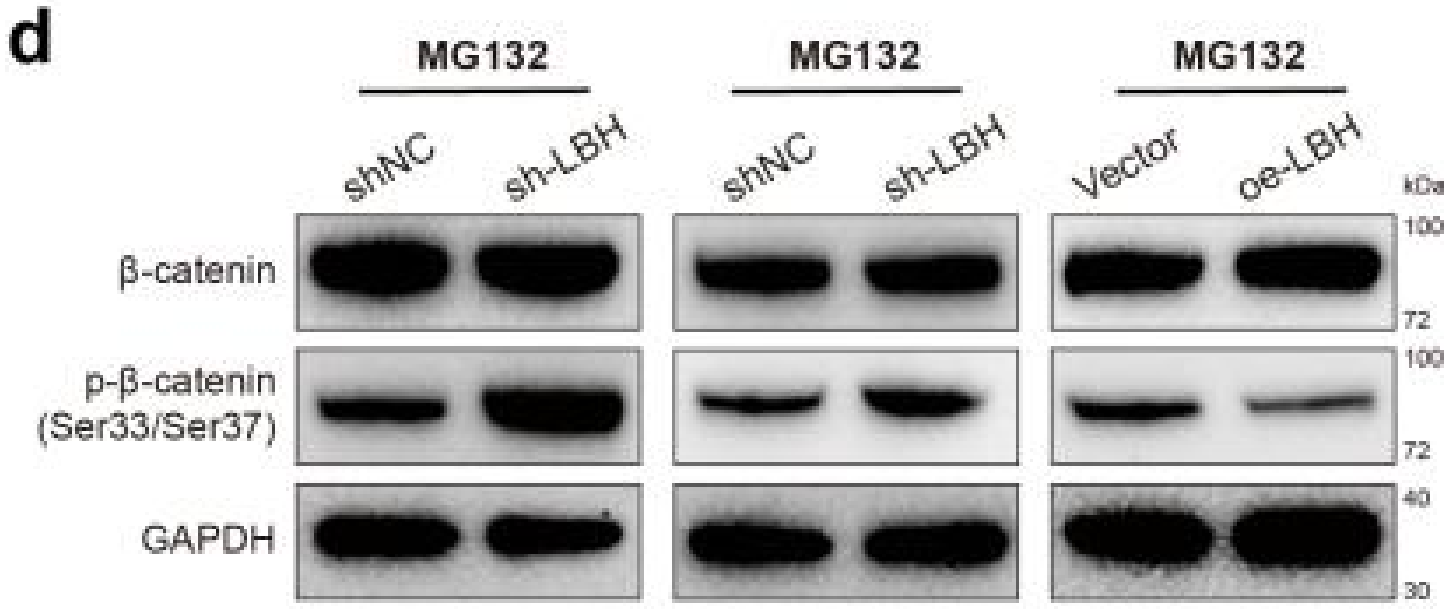
d
MG132
MG132
MG132
shNC
sh-LBH
shNC
sh-LBH
Vector
oe-LBH
kDa
β-catenin
p-β-catenin (Ser33/Ser37)
GAPDH
100
72
100
72
40
30

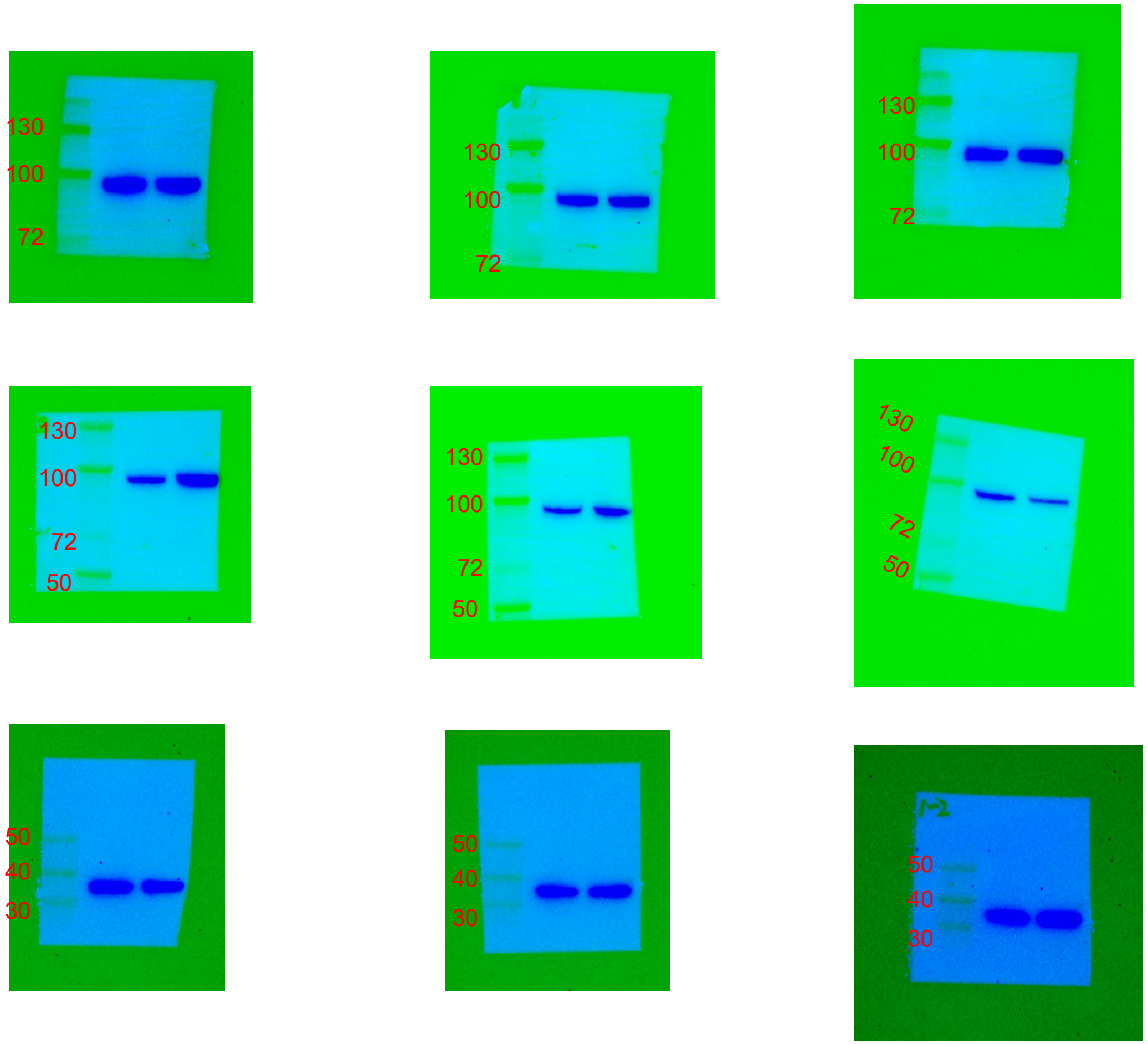
130
100
72
130
100
72
130
100
72
130
100
72
50
130
100
72
50
130
100
72
50
50
40
30
50
40
30
50
40
30

Figure 6e

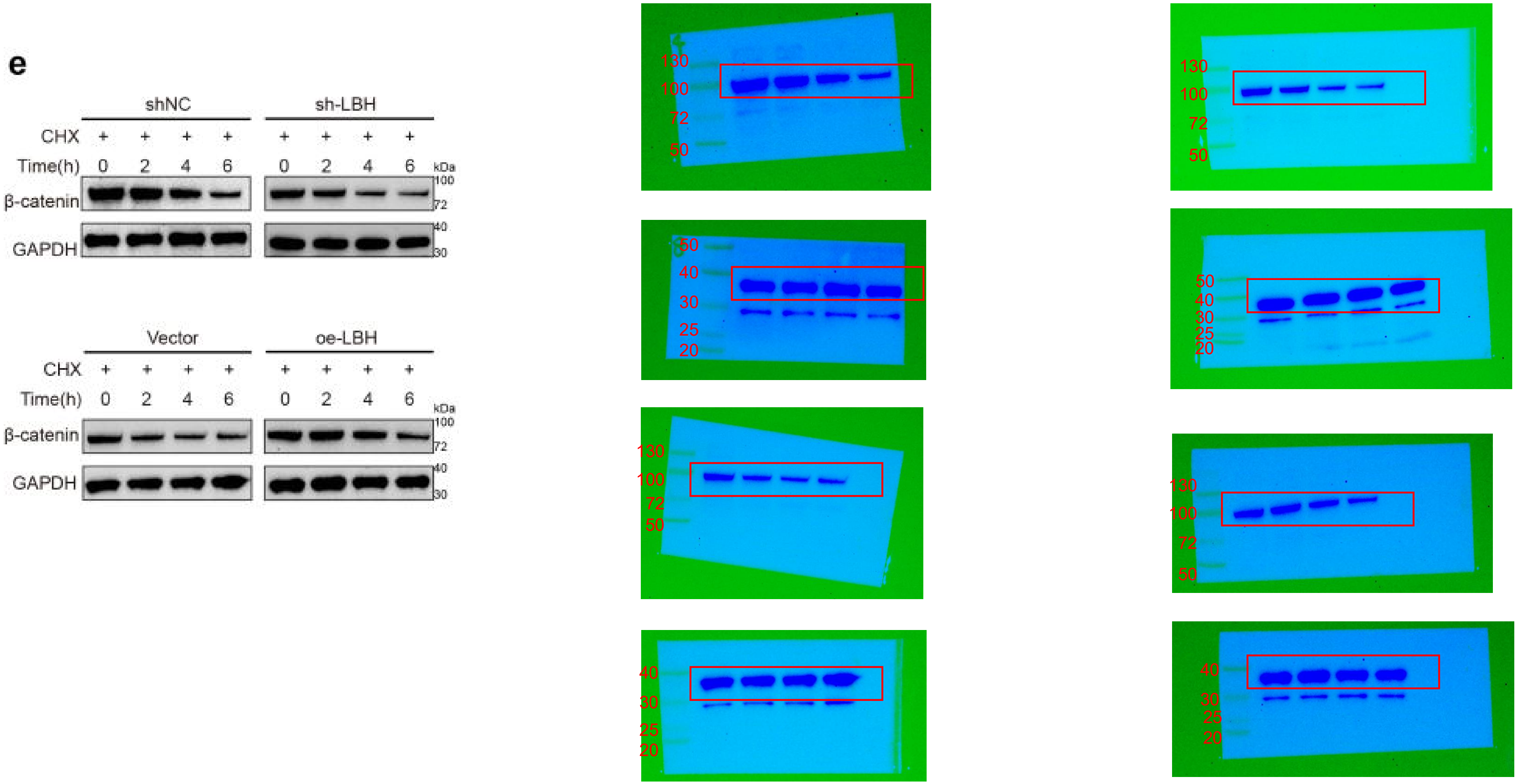
e
shNC
sh-LBH
CHX + + + + + + + +
Time(h) 0 2 4 6 0 2 4 6
kDa
β-catenin
GAPDH
100
72
40
30
Vector
oe-LBH
CHX + + + + + + + +
Time(h) 0 2 4 6 0 2 4 6
kDa
β-catenin
GAPDH
100
72
40
30
130
100
72
50
50
40
30
25
20

Figure 6f

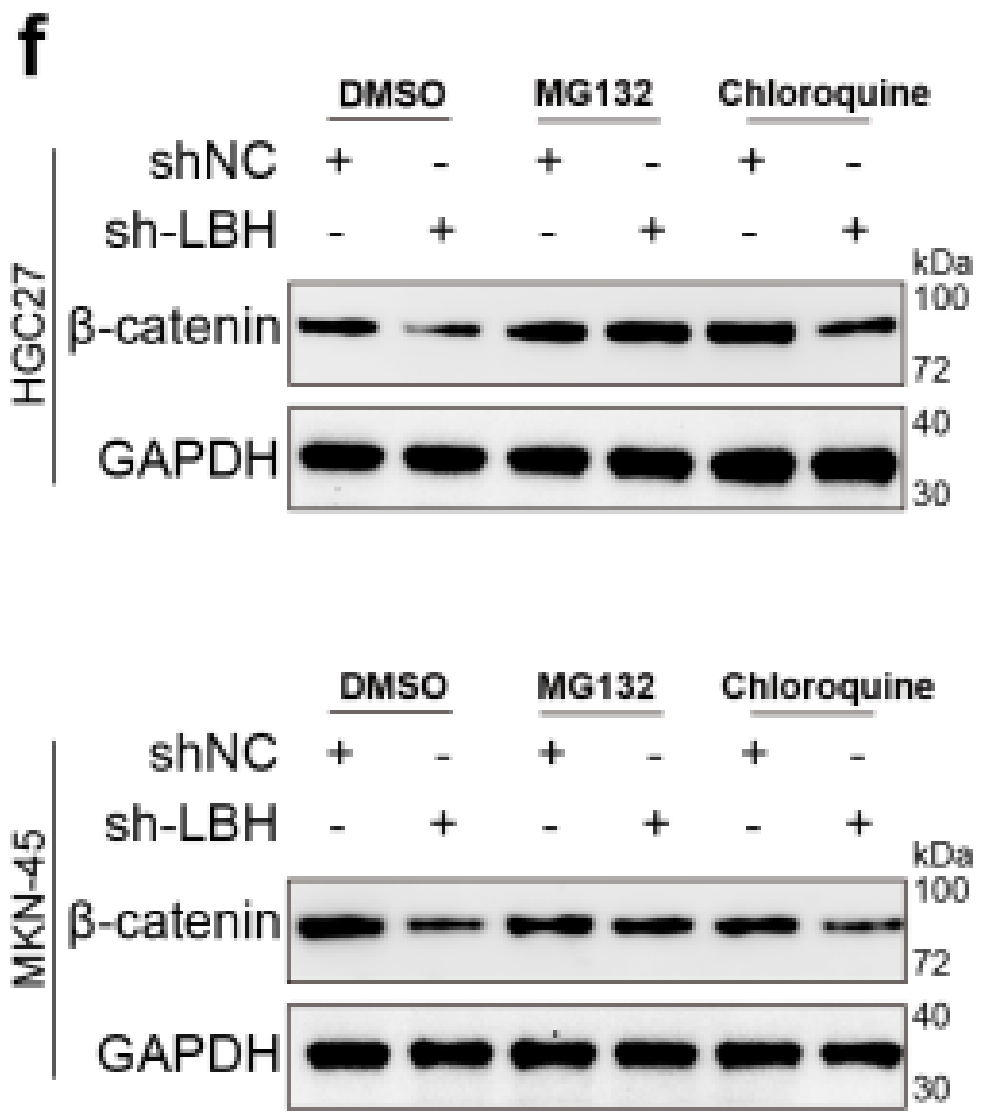

f
DMSO
MG132
Chloroquine
shNC
sh-LBH
HGC27
β-catenin
GAPDH
kDa
100
72
40
30
MKN-45

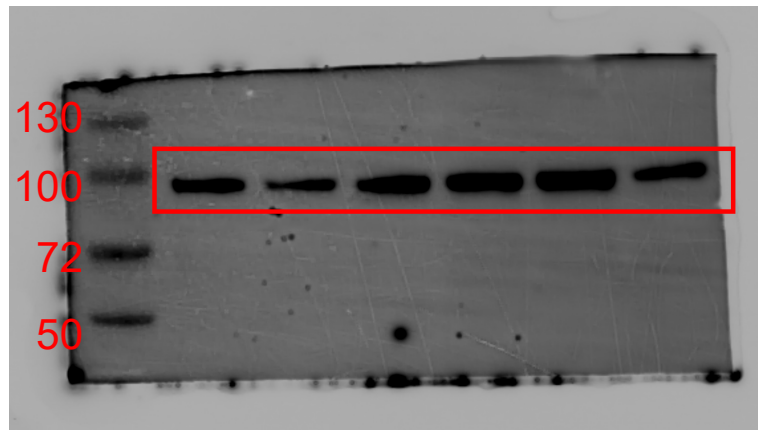

130
100
72
50

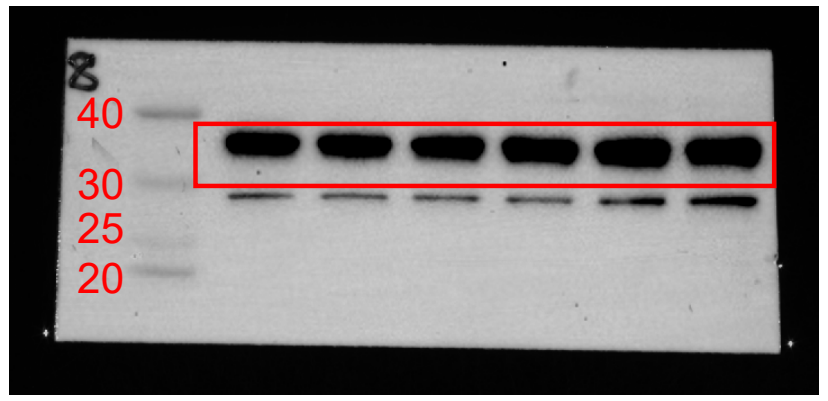

40
30
25
20

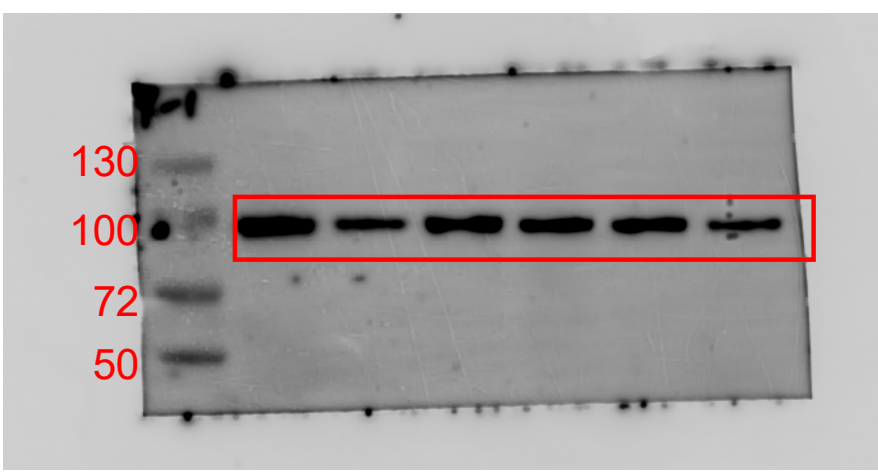

130
100
72
50

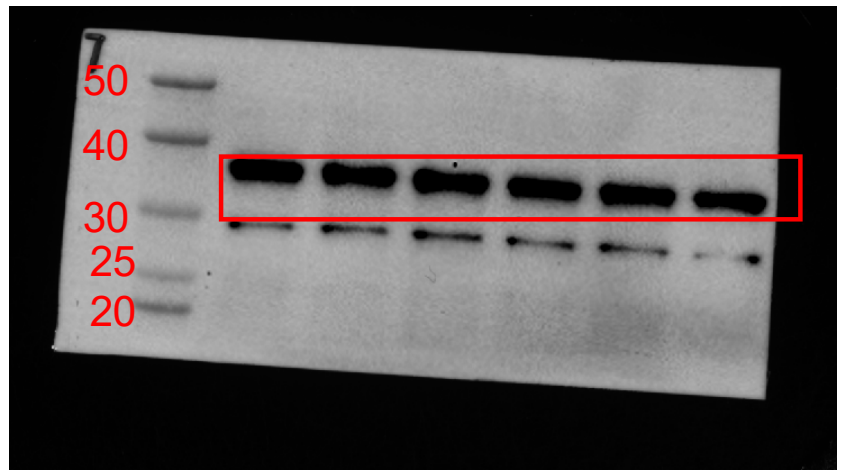

50
40
30
25
20

Figure 6g

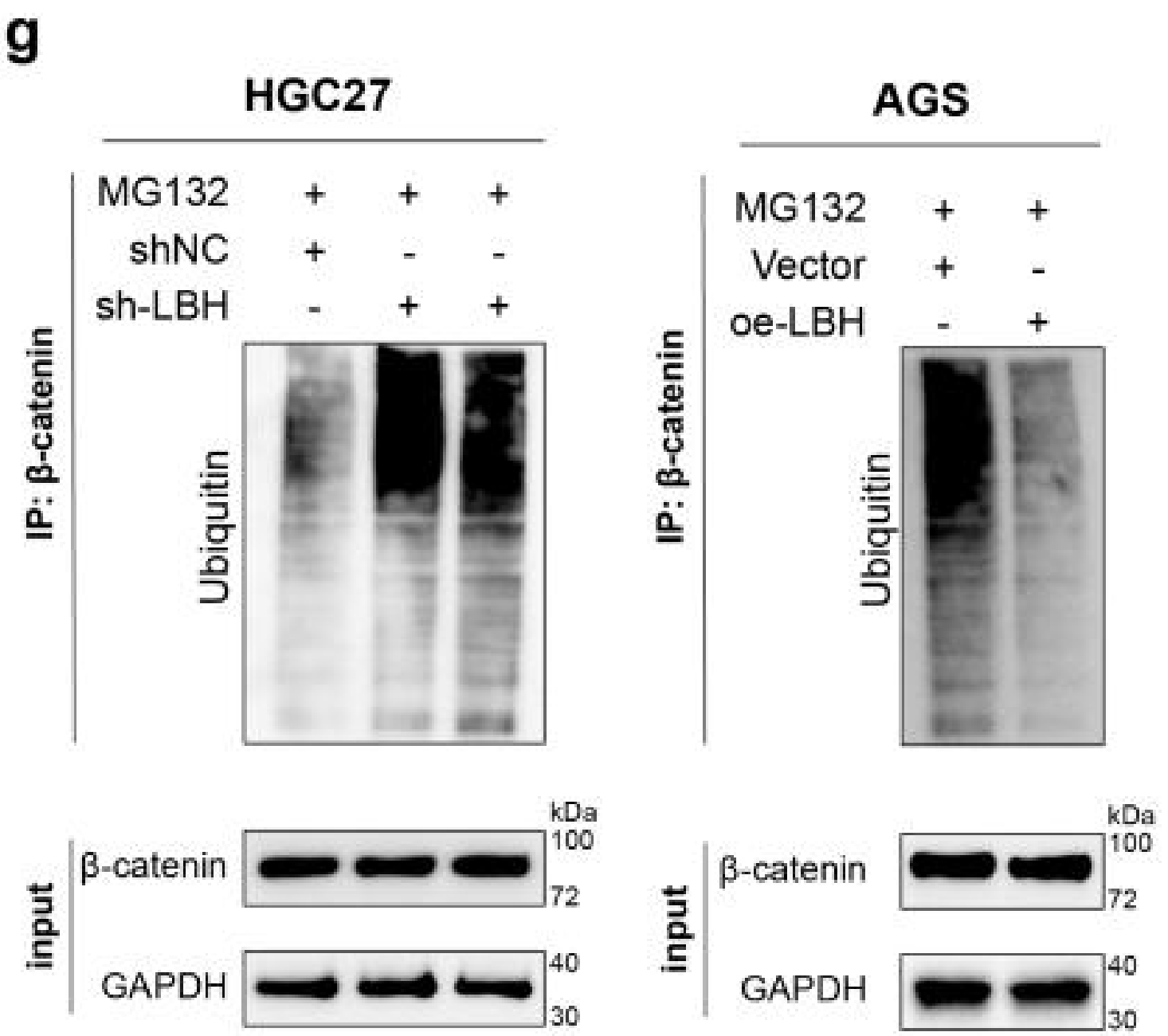
g
HGC27
MG132 + + +
shNC + - -
sh-LBH - + +
IP: β-catenin
Ubiquitin
input
β-catenin
GAPDH
kDa
100
72
40
30
AGS
MG132 + +
Vector + -
oe-LBH - +
IP: β-catenin
Ubiquitin
input
β-catenin
GAPDH
kDa
100
72
40
30

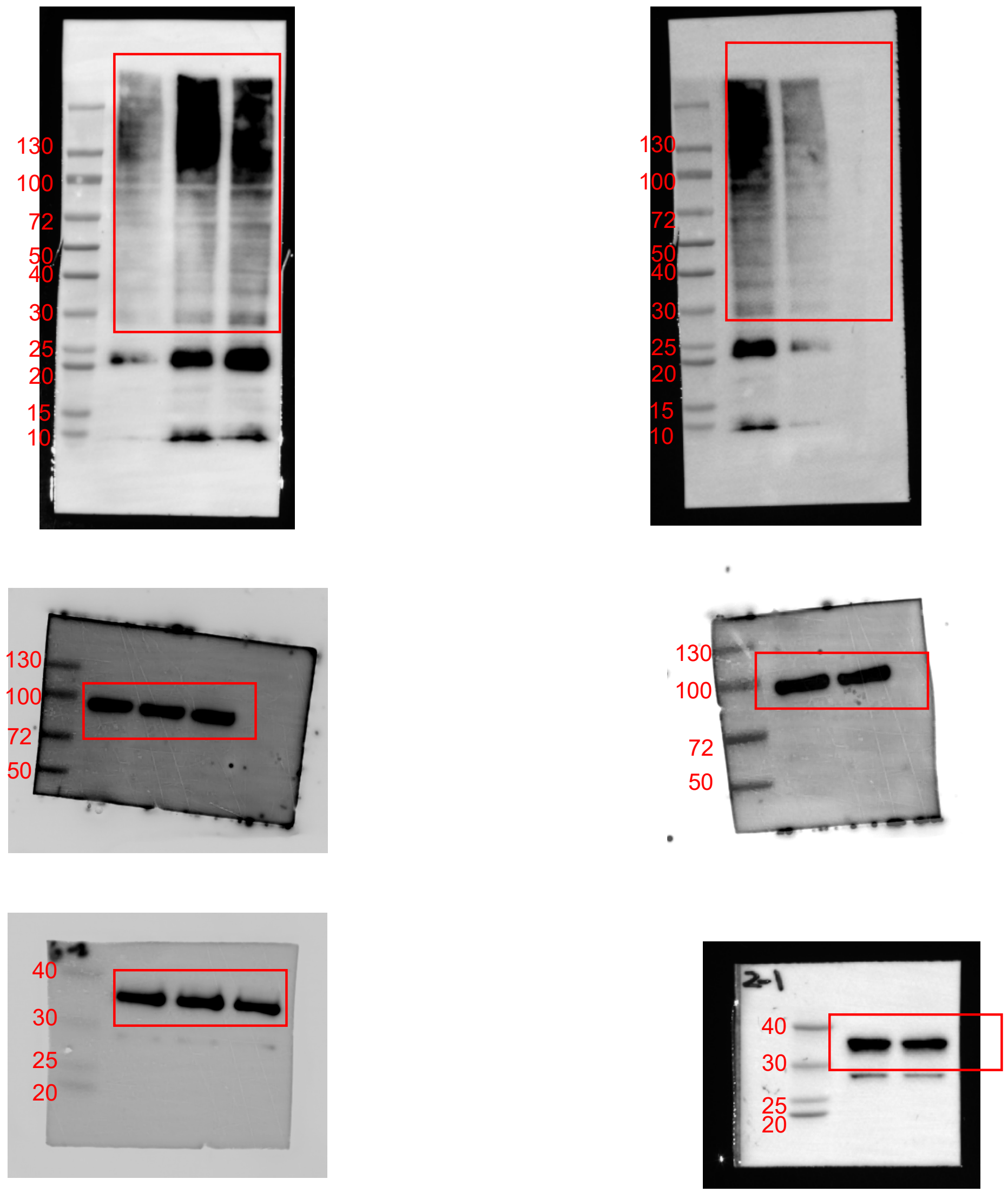
130
100
72
50
40
30
25
20
15
10
130
100
72
50
40
30
25
20
15
10
130
100
72
50
130
100
72
50
40
30
25
20
2-1
40
30
25
20

Figure 7e

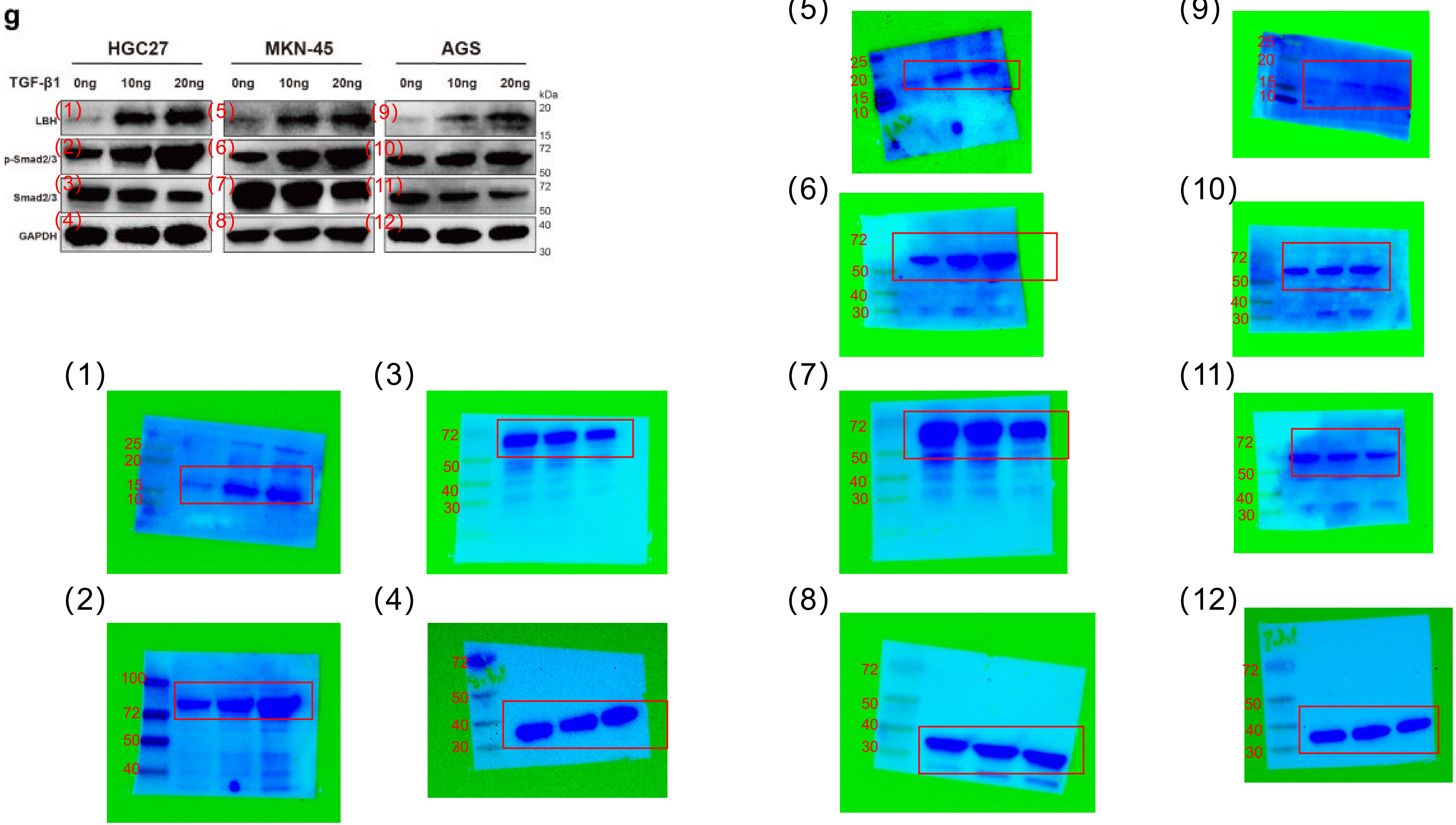
g
HGC27
MKN-45
AGS
TGF-β1
0ng
10ng
20ng
kDa
LBH
p-Smad2/3
Smad2/3
GAPDH
(1)
(2)
(3)
(4)
(5)
(6)
(7)
(8)
(9)
(10)
(11)
(12)

Figrue 8c

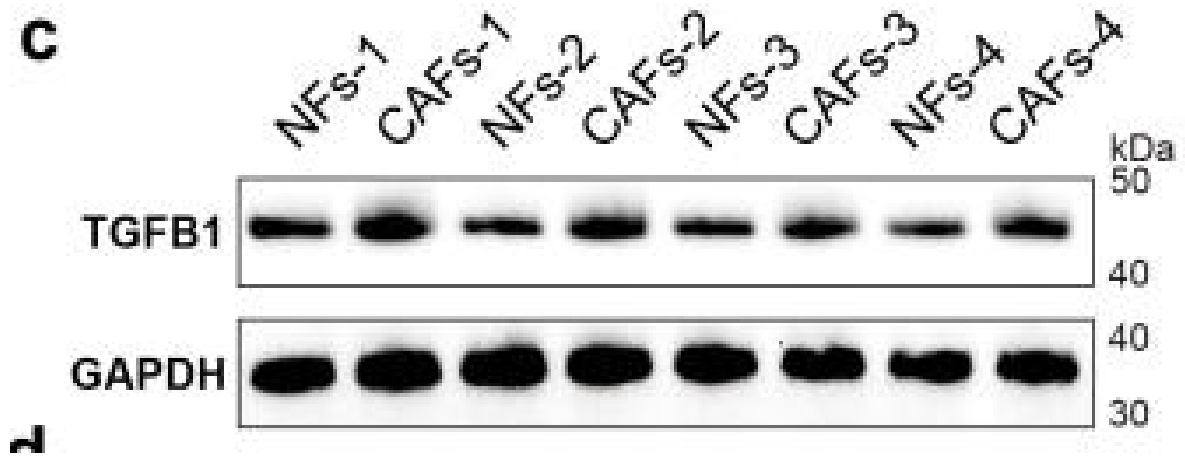
c
NFs-1
CAFs-1
NFs-2
CAFs-2
NFs-3
CAFs-3
NFs-4
CAFs-4
kDa
50
40
TGFB1
40
30
GAPDH

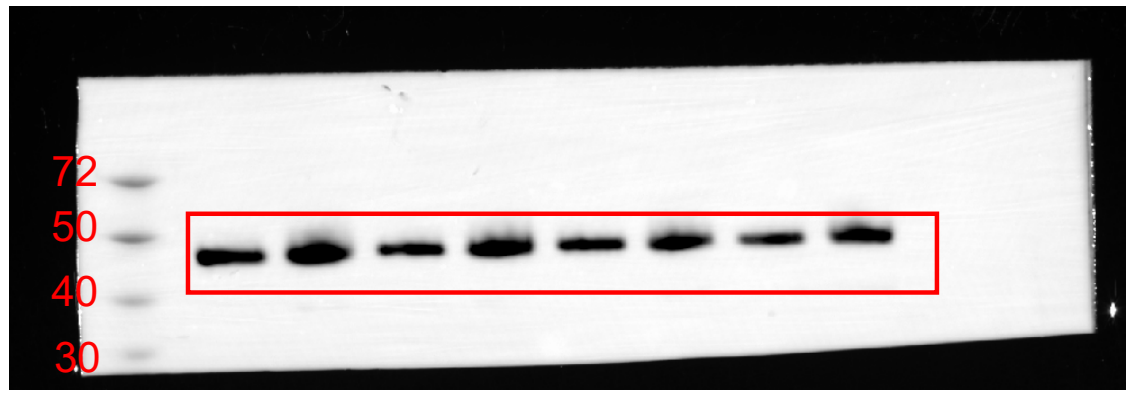
72
50
40
30

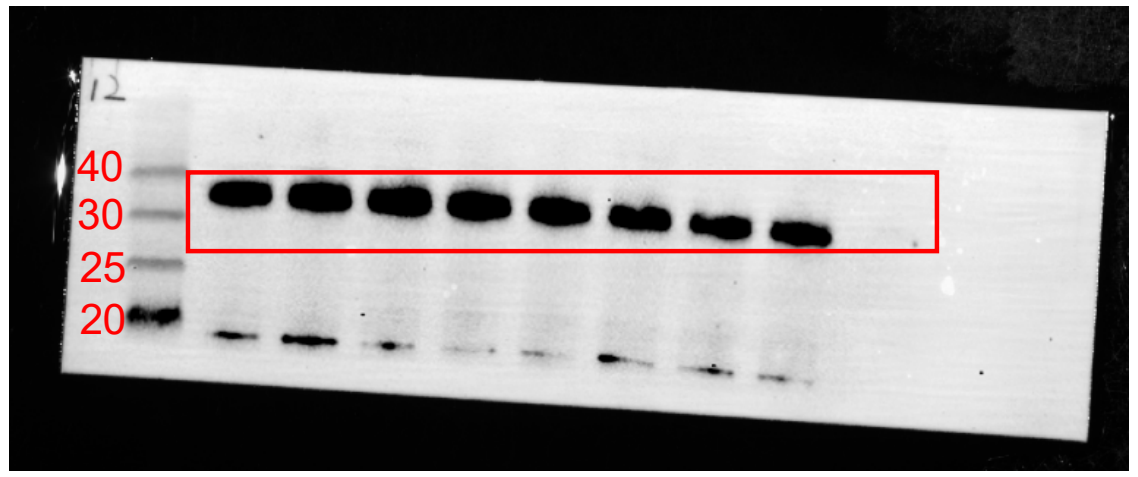
40
30
25
20

Supplementary Figure 4b

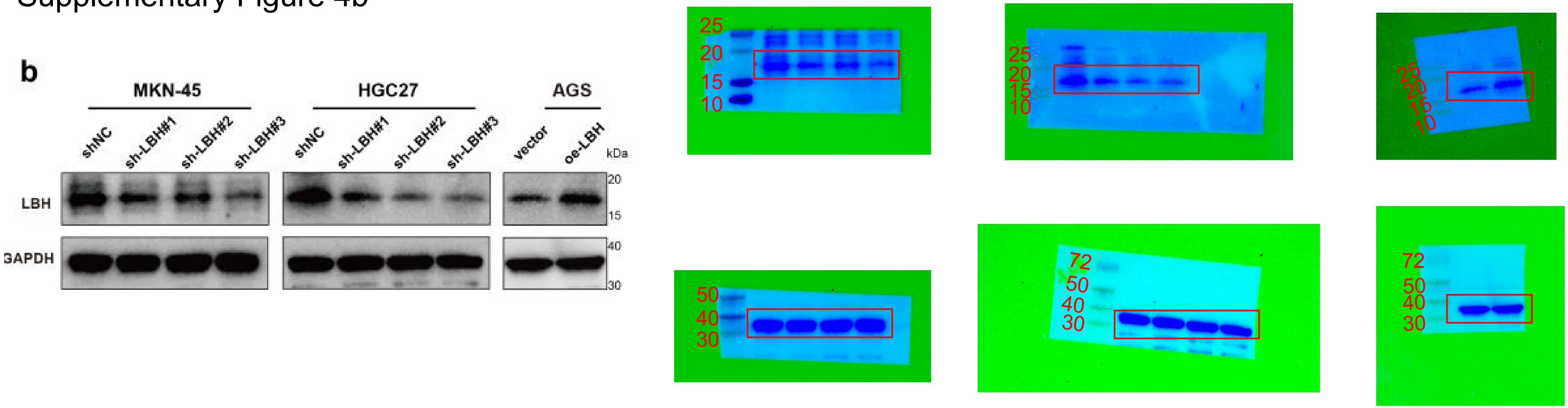

Supplementary Figure 7c

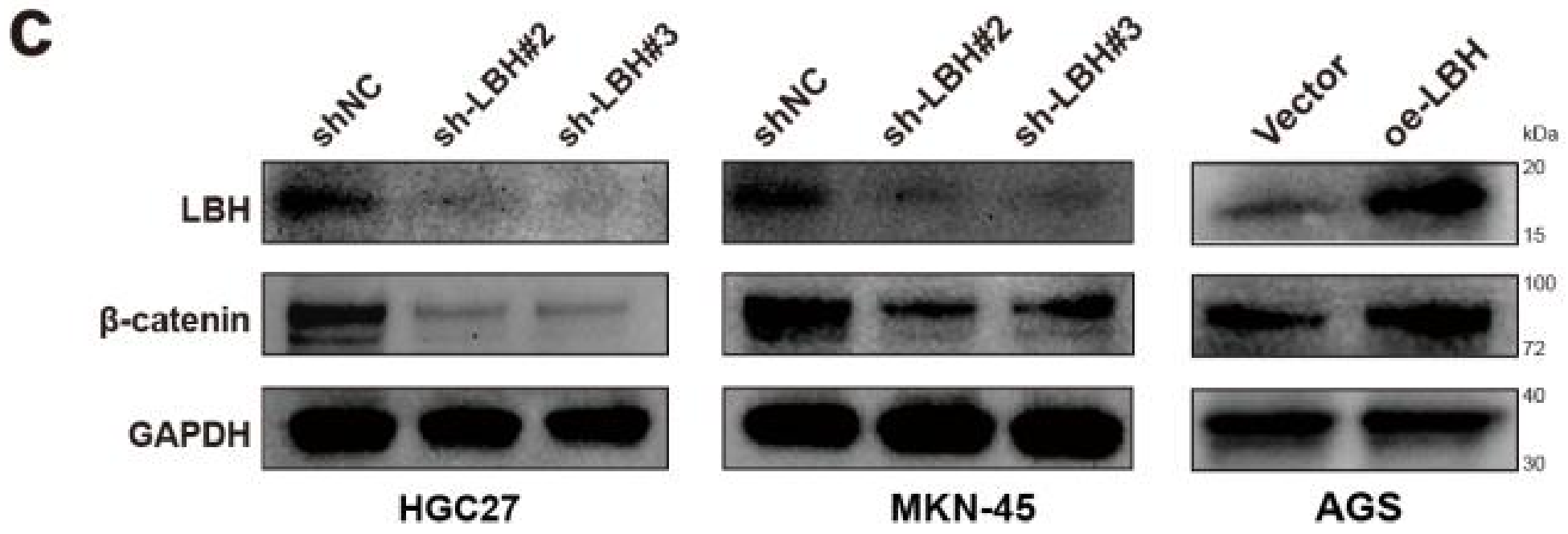

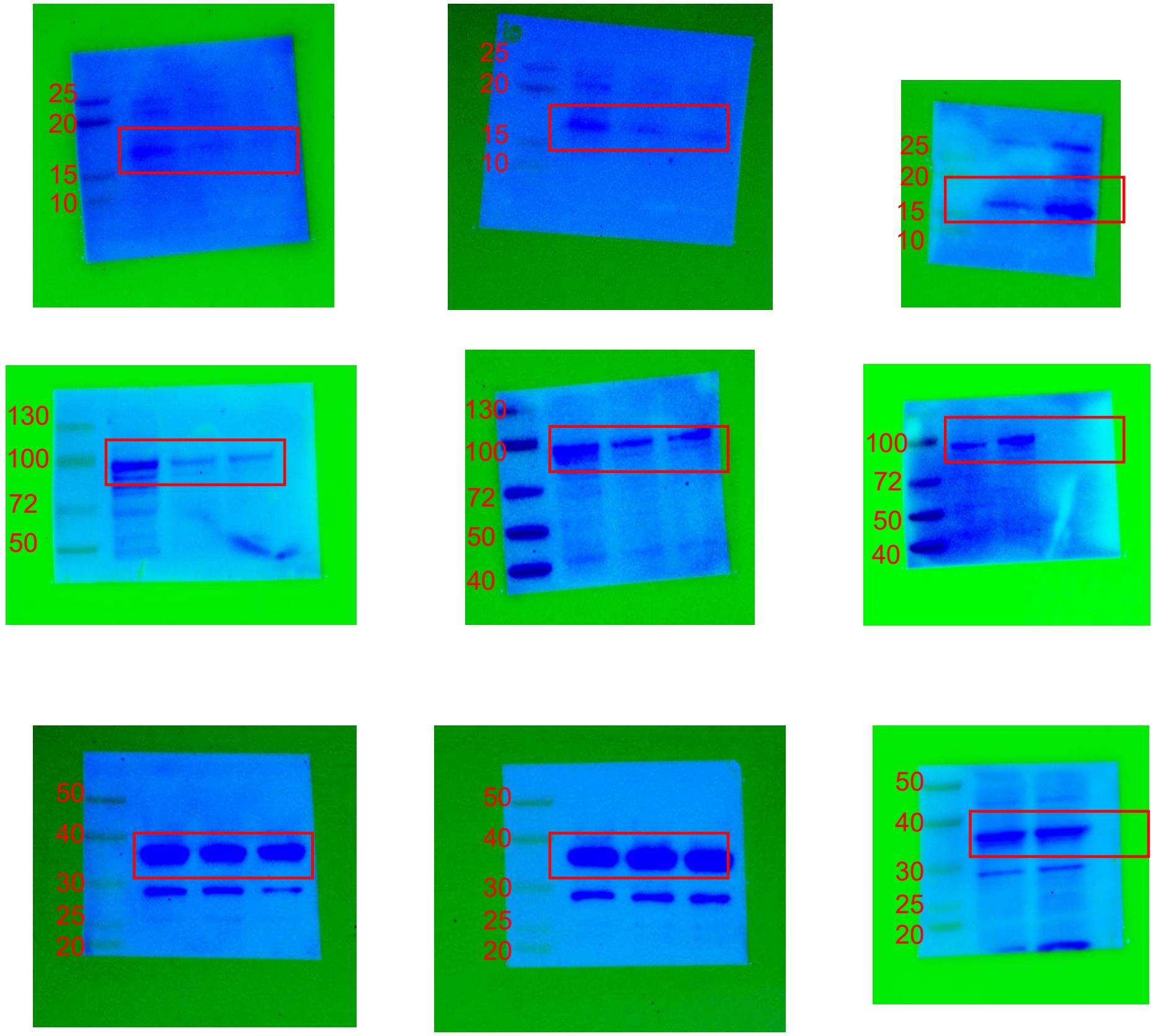

Supplementary Figure 7e

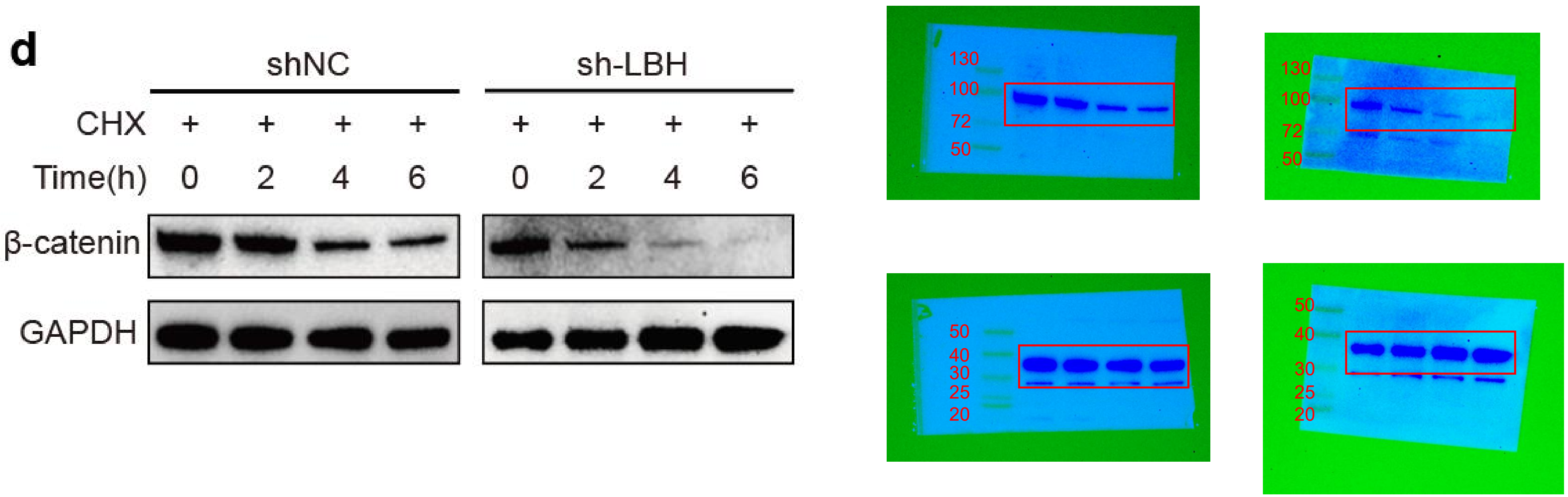

Supplementary Figure 7f

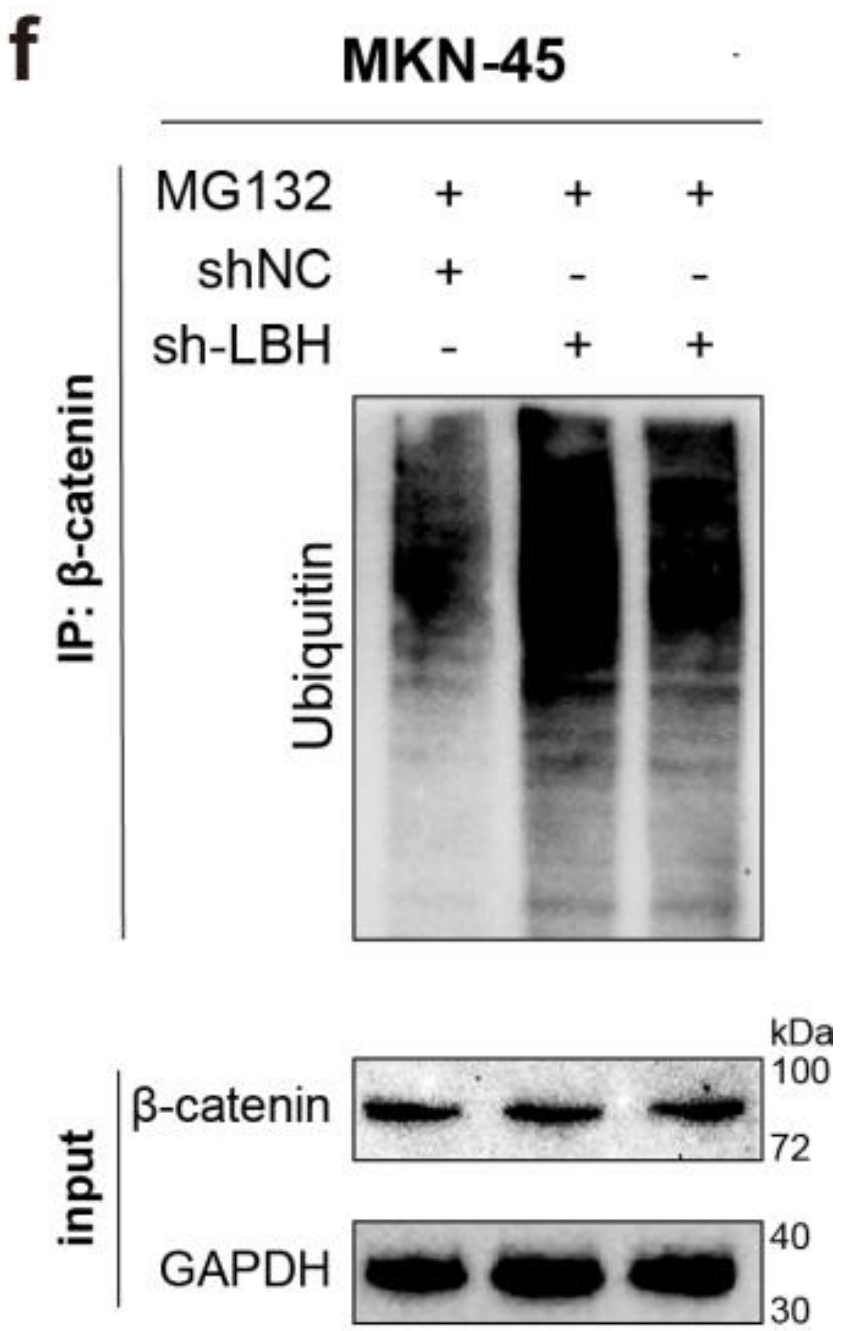

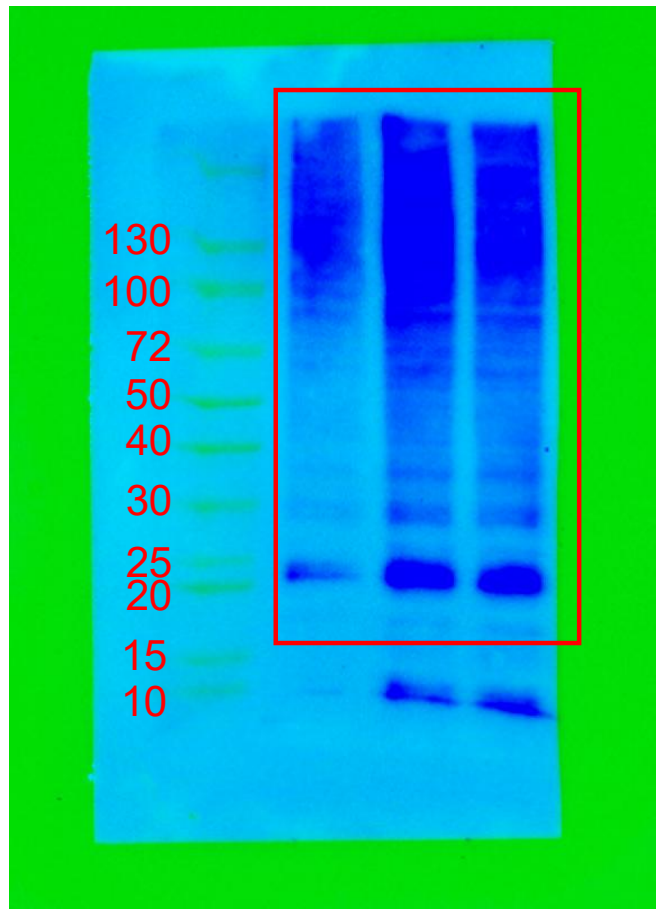

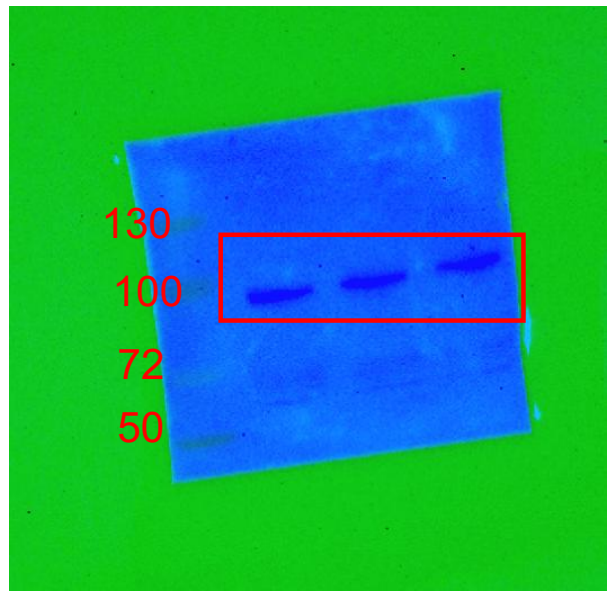

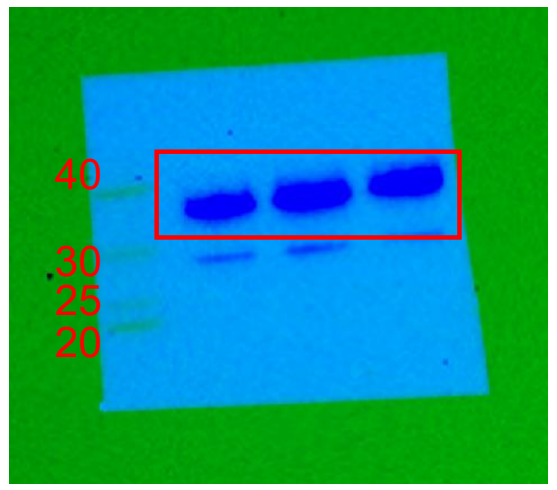

Supplementary Figure 8j

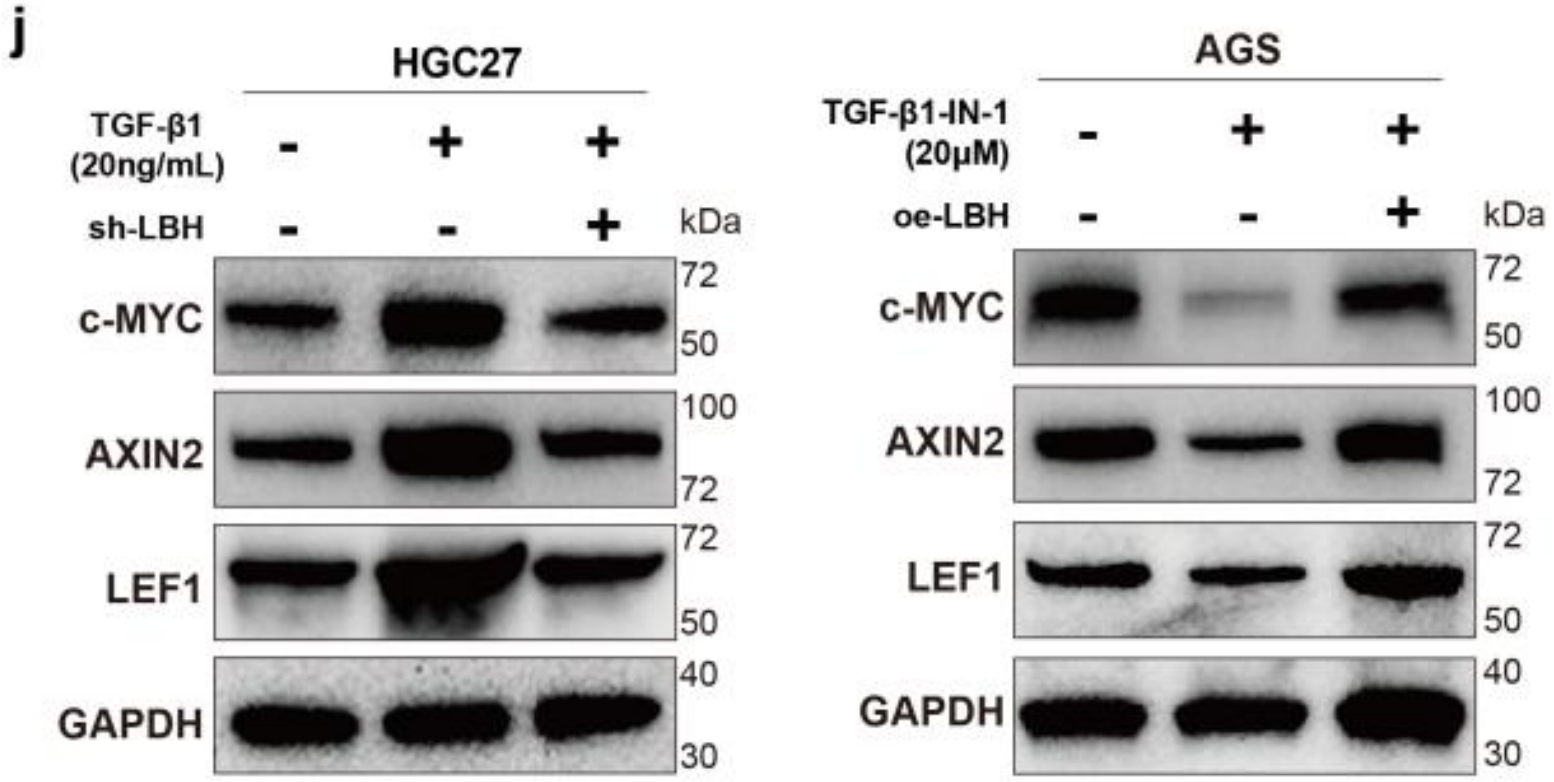

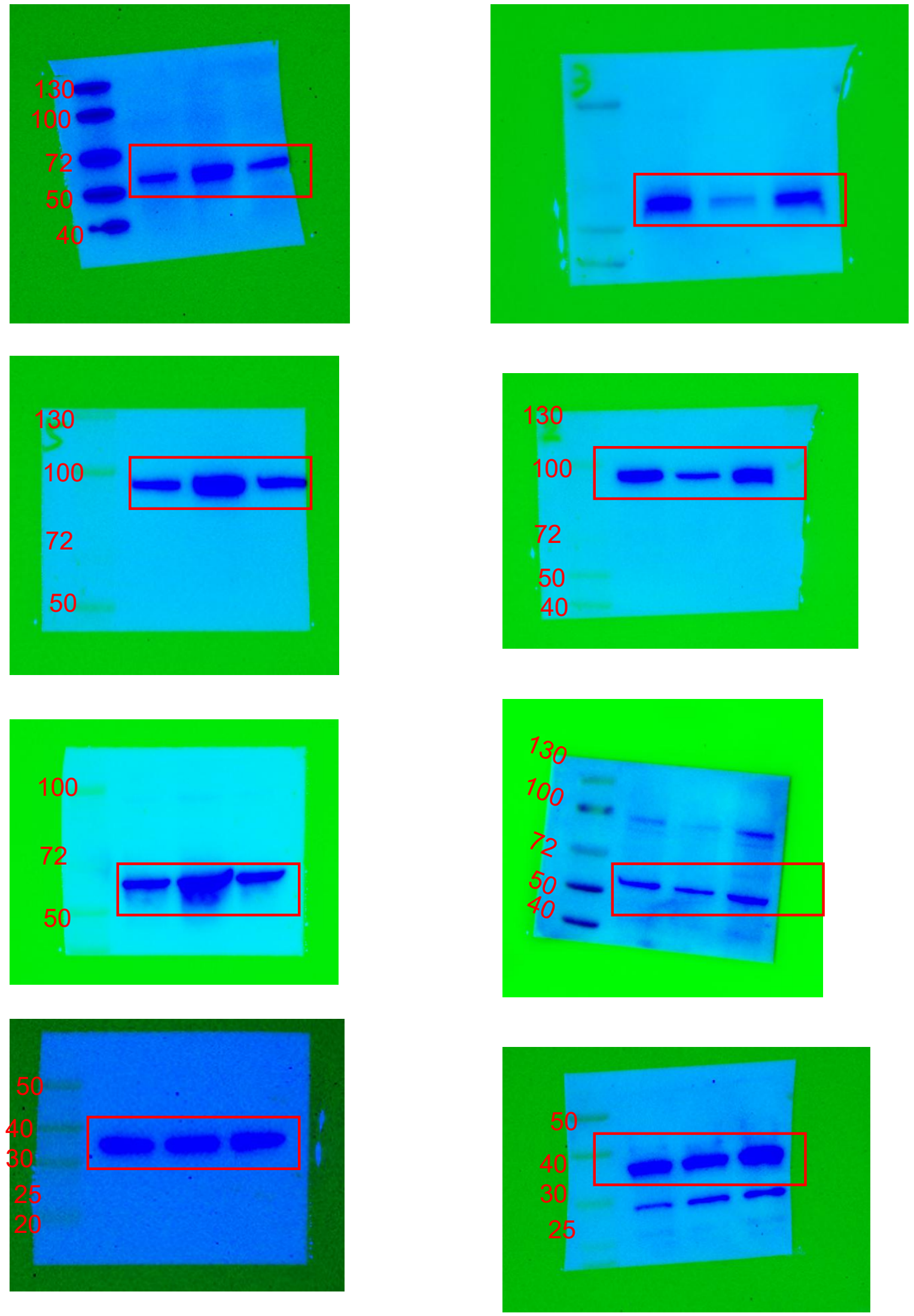

Supplementary Figure 10a

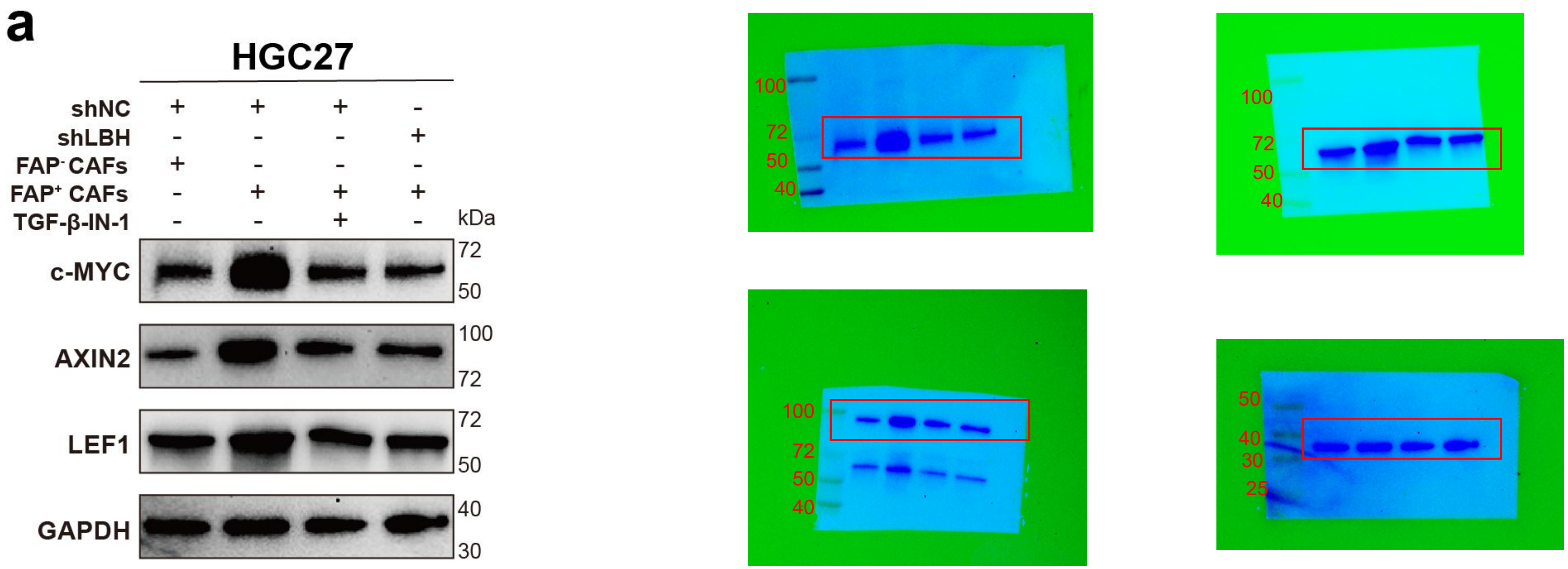

Supplementary Figure 12d

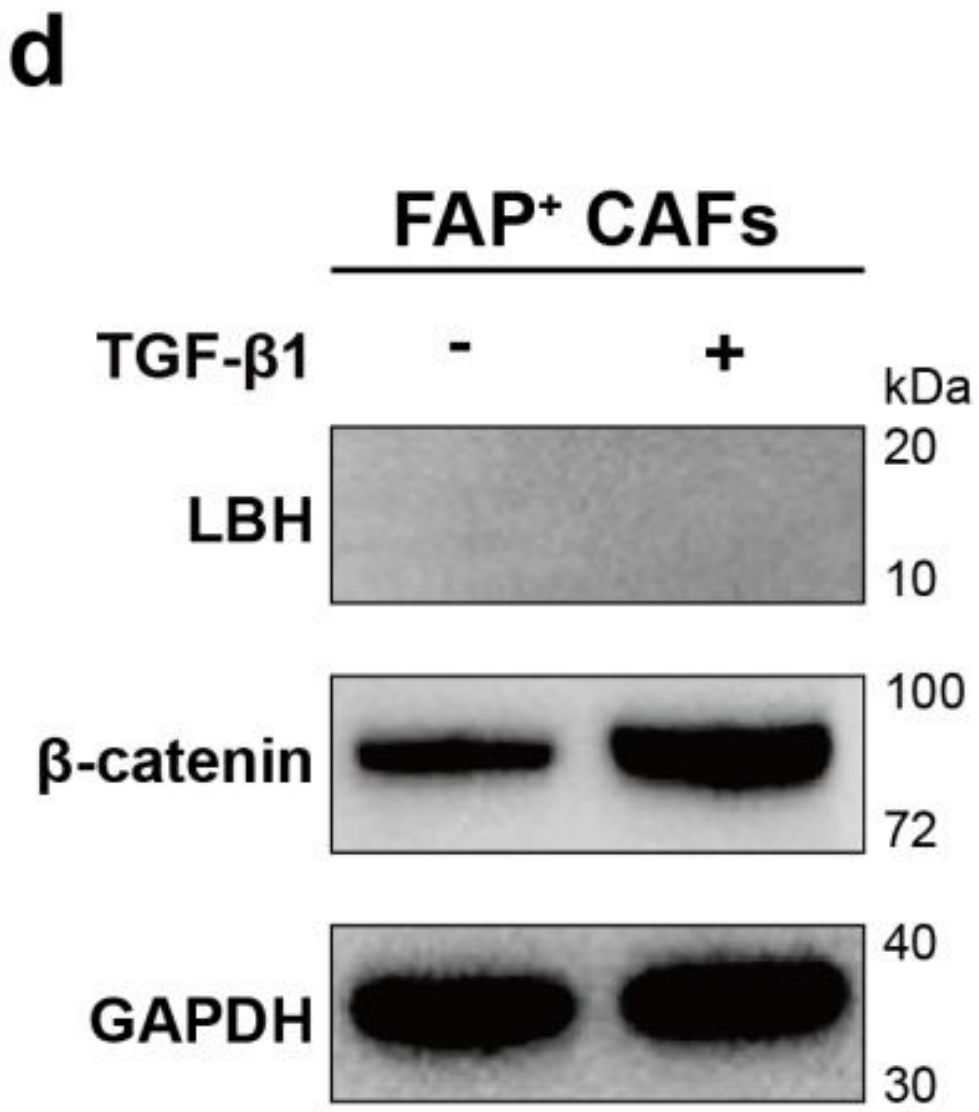

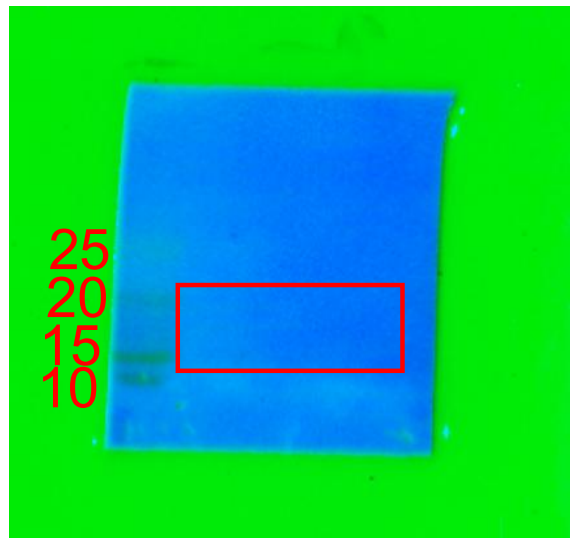

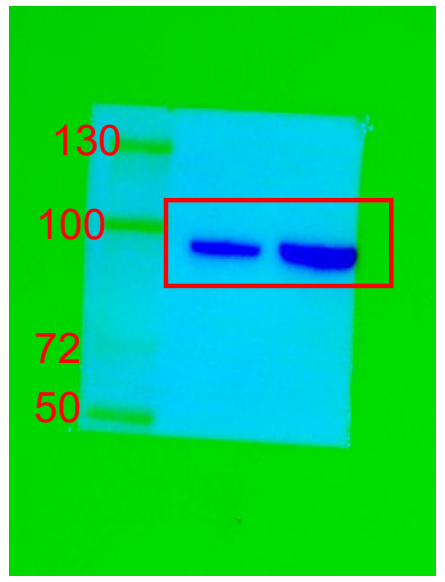

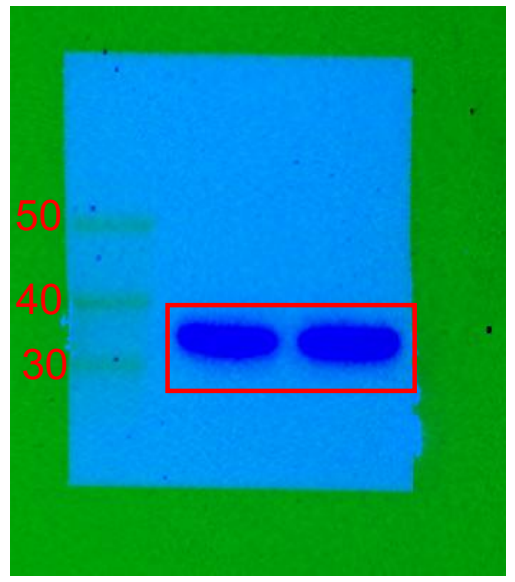

Supplement: Supplementary file 1 [file 41419_2026_9150_MOESM3_ESM.pdf]
