# Supplementary material for: CAF-derived TGF-β1 activates an LBH-mediated β-catenin stabilization program to drive gastric cancer progression
Source: Cell Death Dis. 2026 Aug 6;17(1):935. doi: 10.1038/s41419-026-09150-w (PMC13731647; doi:10.1038/s41419-026-09150-w)
Supplement: Supplementary file 2 [file 41419_2026_9150_MOESM1_ESM.docx]

# *Supplementary Materials*

## Supplementary Tables

Supplementary Table 1. Clinical information of the FUAPH cohorts.

Supplementary Table 2. Clinical characteristics of the single-cell dataset.

Supplementary Table 3. Clinical information of the transcriptomic cohorts.

Supplementary Table 4. Primer sequences used for qPCR in this study.

Supplementary Table 5. Antibody information used in this study.

## Supplementary Figures

**Supplementary Figure 1. Single-cell transcriptomic landscape and cell type identification. (a)** UMAP projection of all sequenced cells, colored by unsupervised Seurat clusters (0-22). **(b)** Dot plot showing the expression of canonical marker genes across major cell lineages. Dot size and color intensity indicate the percentage of cells expressing the gene and average scaled expression level, respectively. **(c)** Large-scale chromosomal copy number variations (CNVs) inferred by *inferCNV*. B cells served as the normal reference (top panel) to evaluate the malignancy of eight epithelial subclusters (Epi_01-08, bottom panel). Red and blue indicate chromosomal amplification and deletion, respectively. **(d)** Pseudotime trajectory analysis of the epithelial subclusters (Epi_01-08) mapped onto the UMAP embedding, revealing their potential developmental state transitions.

**Supplementary Figure 2. Consensus clustering and differential expression analysis. (a)** Delta area plot showing the relative change in the area under the cumulative distribution function (CDF) curve for k = 2 to 9, used to determine the optimal number of clusters for the samples. **(b)** Volcano plot of the differentially expressed genes (DEGs) between the identified sample groups. Red and green triangles represent significantly up-regulated and down-regulated genes, respectively. **(c)** Network visualization of functional enrichment analysis for the DEGs. Central red square nodes represent significantly enriched pathways and terms (e.g., extracellular matrix organization, focal adhesion, and ECM-receptor interaction), while peripheral blue circular nodes represent the associated genes. The size of the square nodes correlates with the number of genes involved. **(d)** Heatmap illustrating the distinct expression profiles of the top DEGs between the two identified sample groups (cluster 1 in blue vs. cluster 2 in red).

**Supplementary Figure 3.** Relative LBH mRNA expression in ges-1 (normal gastric epithelial cells) and gastric cancer cell lines (AGS, N87, SNU1, HGC27, MKN-45) determined by qRT-PCR (2^^-ΔΔCt^ method). Data are shown as mean ± SD (n = 3).

**Supplementary Figure 4. Validation of transfection efficiency and the pro-proliferative effect of LBH overexpression *in vitro*. (a-b)** The transfection efficiency of LBH knockdown (sh-LBH#1-3) in HGC27 and MKN-45 cells, and LBH overexpression (oe-LBH) in AGS cells, was confirmed by RT-qPCR **(a)** and Western blot **(b)**. GAPDH served as the loading control. **(c-f)** The pro-proliferative effects of LBH overexpression in AGS cells were evaluated by CCK-8 assay **(c)**, colony formation assay **(d)**, EdU incorporation assay **(e)**, and 3D tumor spheroid formation assay over 7 days **(f)**. **(g)** Flow cytometry analysis of cell cycle distribution (G1, S, and G2 phases) in AGS cells. **(h-j)** Quantitative protein expression analysis of LBH and cell cycle inhibitors (p16 and p21) following LBH knockdown in HGC27 (h) and MKN-45 **(i)** cells, or LBH overexpression in AGS cells **(j)**. Data are presented as mean ± SD from three independent experiments (n = 3). Statistical significance was analyzed using Student’s t-test or one-way ANOVA. ns, not significant; ***P* < 0.01, ****P* < 0.001. Abbreviations: CCK-8, Cell Counting Kit-8; EdU, 5-ethynyl-2'-deoxyuridine; SD, standard deviation.

**Supplementary Figure 5. LBH overexpression promotes gastric cancer cell migration, invasion, and epithelial-mesenchymal transition *in vitro*.** **(a)** Representative images and quantitative analysis of wound healing assays evaluating the migration capacity of AGS cells following LBH overexpression (oe-LBH) compared to vector control over 72 h. **(b)** Transwell invasion assays and relative cell quantification evaluating the invasive capacity of AGS cells upon LBH overexpression. **(c, d)** RT-qPCR analysis of the relative mRNA expression levels of epithelial-mesenchymal transition (EMT) marker genes (*CDH1, CDH2, SNAI1, SNAI2, VIM*) following LBH knockdown in MKN-45 cells **(c)** and LBH overexpression in AGS cells **(d)**. Data are presented as mean ± SD from three independent experiments (n = 3). Statistical significance was analyzed using Student’s t-test or one-way ANOVA. ****P* < 0.001. Abbreviations: EMT, epithelial-mesenchymal transition; RT-qPCR, reverse transcription-quantitative polymerase chain reaction; SD, standard deviation.

**Supplementary Figure 6. Quantitative analyses of Wnt/β-catenin signaling pathway activation regulated by LBH.** **(a–c)** Quantitative Western blot analysis of the relative protein expression levels of canonical Wnt downstream targets (c-MYC, AXIN2, and LEF1) in LBH-knockdown HGC27 **(a)** and MKN-45 **(b)** cells treated with the Wnt agonist LiCl (10 mM, 24 h), and in LBH-overexpressing AGS cells **(c)** treated with the Wnt antagonist XAV-939 (10 μM, 24 h). **(d)** Quantitative analysis of the nuclear-to-cytoplasmic (N/C) protein ratio of β-catenin in MKN-45 cells following LBH knockdown, with or without LiCl (10 mM, 24 h) treatment. **(e)** TCF/LEF dual-luciferase reporter assays evaluating downstream Wnt transcriptional activity in MKN-45 cells under the indicated genetic and pharmacological interventions. Data are presented as mean ± SD from three independent experiments (n = 3). Statistical significance was evaluated using one-way ANOVA. ***P* < 0.01, ****P* < 0.001. Abbreviations: SD, standard deviation.

**Supplementary Figure 7. LBH modulates β-catenin protein stability without altering its transcriptional expression.** **(a)** RT-qPCR analysis of *CTNNB1* mRNA levels in MKN-45 cells following LBH knockdown. **(b)** Spearman correlation analysis between *LBH* and *CTNNB1* mRNA expression across four public gastric cancer cohorts (TCGA, ACRG, KUCM, and YUSH). **(c)** Western blot analysis and corresponding quantification of LBH and β-catenin protein levels following LBH knockdown (in HGC27 and MKN-45 cells) or LBH overexpression (in AGS cells). GAPDH serves as the internal loading control. **(d)** Representative immunofluorescence images showing the subcellular colocalization of LBH (yellow) and β-catenin (red) in HGC27 cells. Nuclei were counterstained with DAPI (blue). **(e)** Cycloheximide (CHX, 50 μg/mL) chase assay and corresponding quantitative line graph evaluating the protein half-life of β-catenin in MKN-45 cells following LBH knockdown over a 0–6 h time course. **(f)** Ubiquitination assay detecting the polyubiquitination levels of β-catenin in MKN-45 cells with or without LBH knockdown, following pre-treatment with the proteasome inhibitor MG132 (20 μM). Data are presented as mean ± SD from three independent experiments (n = 3). Statistical significance was determined using Student’s t-test or one-way ANOVA. ns, not significant; ****P* < 0.001. Abbreviations: CHX, cycloheximide; SD, standard deviation.

**Supplementary Figure 8. Supplemental data supporting the TGF-β1/SMAD2/3/LBH signaling axis and Wnt pathway activation.** **(a)** Venn diagram illustrating the intersection of six transcription factor prediction databases (KnockTF, JASPAR, ChIP_Atlas, ENCODE, CHEA, and GTRD), identifying SMAD2 and SMAD3 as potential upstream regulators of LBH. **(b)** Dual-luciferase reporter assay assessing the transcriptional activity of wild-type (WT) or mutant (mut) LBH promoters in AGS cells co-transfected with SMAD2/3 overexpression plasmids. **(c)** Spearman correlation analysis between *TGFB1* and *LBH* mRNA expression in the TCGA and ACRG cohorts. **(d)** Cell viability evaluated by CCK-8 assays in HGC27 cells treated with recombinant TGF-β1 (20 ng/mL) with or without LBH knockdown (sh-LBH), and in AGS cells treated with the TGF-β receptor inhibitor TGF-β1-IN-1 (20 μM) with or without LBH overexpression (oe-LBH). **(e–h)** Functional rescue assays including EdU incorporation **(e)**, colony formation **(f)**, Transwell invasion **(g)**, and wound healing **(h)**, evaluating the malignant phenotypes of AGS cells treated with TGF-β1-IN-1 (20 μM) with or without ectopic LBH overexpression. **(i)** TCF/LEF dual-luciferase reporter assay evaluating downstream Wnt transcriptional activity in AGS cells under the indicated pharmacological (TGF-β1-IN-1, 20 μM) and genetic (oe-LBH) interventions. **(j)** Western blot analysis and corresponding quantitative analysis of canonical Wnt target proteins (c-MYC, AXIN2, and LEF1). Left panel: HGC27 cells treated with TGF-β1 (20 ng/mL) ± sh-LBH. Right panel: AGS cells treated with TGF-β1-IN-1 (20 μM) ± oe-LBH. GAPDH serves as the internal loading control. Data are presented as mean ± SD from three independent experiments (n = 3). Statistical significance was analyzed using Student’s t-test or one-way ANOVA. ****P* < 0.001. Abbreviations: CCK-8, Cell Counting Kit-8; EdU, 5-ethynyl-2'-deoxyuridine; SD, standard deviation.

**Supplementary Figure 9. FAP^+^ CAFs interact with gastric cancer epithelial cells predominantly via the TGF-β signaling pathway. (a)** Dot plot showing the expression of specific marker genes across distinct fibroblast subclusters. **(b-c)** UMAP feature plots **(b)** and Spearman correlation analysis **(c)** demonstrating the co-expression and significant positive correlation between *FAP* and *TGFB1* mRNA levels in the fibroblast populations. **(d-f)** *CellChat* analysis inferring intercellular communications. Scatter plot of overall incoming and outgoing interaction strengths **(d)**, network visualization of the TGF-β signaling pathway between CAF subtypes and epithelial subclusters **(e)**, and detailed ligand-receptor pair probabilities **(f)**. **(g)** Flow cytometric verification of the purity of FAP⁻ and FAP⁺ CAFs isolated via magnetic-activated cell sorting (MACS), based on APC-FAP fluorescence intensity.

**Supplementary Figure 10. FAP⁺ CAFs activate canonical Wnt target genes in gastric cancer cells via the paracrine TGF-β1/LBH axis. (a)** Representative Western blot analysis of canonical Wnt target proteins (c-MYC, AXIN2, and LEF1) in HGC27 cells. The HGC27 cells (stably transfected with shNC or sh-LBH) were co-cultured with either primary FAP⁻ CAFs or FAP⁺ CAFs. Where indicated, the TGF-β receptor inhibitor (TGF-β-IN-1) was added to the FAP⁺ CAFs co-culture system to block paracrine signaling. GAPDH served as the internal loading control. **(b)** Densitometric quantification of the relative protein expression levels corresponding to the Western blot in **(a)**. Data are presented as mean ± SD from three independent biological replicates (n = 3). Statistical significance was determined using one-way ANOVA followed by Tukey’s multiple comparisons test. *** *P* < 0.001.

**Supplementary Figure 11. FAP⁺ CAFs promote tumor proliferation, EMT, and Wnt/β-catenin signaling in vivo via the TGF-β1/LBH axis.** **(a–d)** Representative immunohistochemistry (IHC) images and corresponding H-score quantification of Ki67 **(a)**, E-cadherin **(b)**, N-cadherin **(c)**, and β-catenin **(d)** in the xenograft tumor tissues from the indicated treatment groups (shNC + FAP⁻ CAFs, shNC + FAP⁺ CAFs, combined with the TGF-β receptor inhibitor TGF-β1-IN-1 or LBH knockdown). Data are presented as mean ± SD from five mice per group (n = 5). Statistical significance was analyzed using one-way ANOVA. ****P* < 0.001. Abbreviations: IHC, immunohistochemistry; EMT, epithelial-mesenchymal transition; SD, standard deviation.

**Supplementary Figure 12. LBH is minimally expressed in CAFs and is not induced by TGF-β1 in an autocrine manner. (a)** Dot plot showing the percentage of expressing cells and average expression levels of *FAP*, *LBH*, and *CTNNB1* across the identified fibroblast sub-clusters based on scRNA-seq data. **(b)** Spearman correlation analysis demonstrating the lack of significant correlation between *FAP* and *LBH* expression levels (*R* = 0.026, *P* = 0.098). **(c)** RT-qPCR analysis evaluating *LBH* mRNA expression in FAP⁺ CAFs treated with or without recombinant TGF-β1. **(d)** Western blot analysis and corresponding quantification of LBH and β-catenin protein levels in FAP⁺ CAFs treated with or without TGF-β1. GAPDH serves as the internal loading control. Data are presented as mean ± SD from three independent experiments (n = 3). Statistical significance was analyzed using Student’s t-test. ns, not significant; ****P* < 0.001. Abbreviations: scRNA-seq, single-cell RNA sequencing; SD, standard deviation.
